# HDAC1 in the Ovarian Granulosa Cells of Tan Sheep Improves Cumulus Cell Expansion and Oocyte Maturation Independently of the EGF-like Growth Factors

**DOI:** 10.3390/biology11101464

**Published:** 2022-10-06

**Authors:** Yaxiu Xu, Shanshan Fan, Yujun Liu, Jiaqi Shi, Xianguo Xie, Xiangyan Wang, Chao Wang, Xinfeng Liu, Guoliang Xia

**Affiliations:** 1 Key Laboratory of Conservation and Utilization of Characteristic Biological Resources in Western China, Ministry of Education, Ningxia University, Yinchuan 750021, China; 2 College of Life Sciences, Ningxia University, Yinchuan 750021, China; 3 State Key Laboratory of Agricultural Biotechnology, College of Biology, China Agricultural University, Beijing 100193, China

**Keywords:** HDAC1, EGF-like growth factor, granulosa cell, oocyte, sheep

## Abstract

**Simple Summary:**

This study aimed to explore the possible role of histone deacetylase 1 (HDAC1), an epigenetic modifying enzyme, in the improvement of oocyte maturation recovered from the Tan sheep of China. We supplemented a specific inhibitor of HDAC1 to in vitro-cultured cumulus oocyte complexes (COCs). The results showed that the cumulus cell expansion and oocyte maturation ratio were impaired, while the production of the epidermal growth factor (EGF) was only slightly changed, despite the fact that the pan-acetylation levels of the histones were significantly elevated. This study implies that HDAC1 may participate in the maturation of oocytes in Tan sheep independently of the classic gonadotrophin-induction pathway.

**Abstract:**

Previous studies have shown that some of the histone deacetylases (HDACs) play diverse roles in the regulation of ovarian somatic cell development, oocyte maturation and early embryonic development in different species including sheep. This study aimed to clarify whether HDAC1 also played pivotal roles in regulating oocyte maturation in Tan sheep. The results showed that HDAC1 was expressed in the nuclei of both the granulosa cells and oocytes of the growing follicles in the Tan sheep’s ovaries. However, the level of HDAC1 was unaffected by luteinizing hormone (LH) induction in cultured granulosa cells. Meanwhile, the specific inhibition of HDAC1 using pyroxamide did not induce significant changes in the expression levels of EGF-like growth factors in vitro, whereas both the cumulus expansion and oocyte maturation of the cultured cumulus oocyte complexes (COCs) were significantly inhibited by pyroxamide. Additionally, the numbers of histone acetylation sites (H4K5, H4K12, H3K14 and H3K9) in ovarian granulosa cells were significantly increased. In conclusion, a constant expression of HDAC1 in the growing follicles of Tan sheep may be pivotal for supporting oocyte growth and maturation, although its action may not be closely correlated with LH induction, nor does it directly affect the expression of the EGF-like factors. Our study implies that there may exist diverse functions of the respective HDACs in modulating female reproduction in sheep.

## 1. Introduction

As the gonadal organ of female mammals, the ovary has important functions of storing and supporting the development of oocytes and secreting various hormones to maintain female endocrine functions [1]. Granulosa cells, which act as supporting cells, are needed for both the development and the maturation of the oocytes [2,3,4]. The role of EGF-like growth factors secreted by follicular granulosa cells in promoting oocyte maturation has been widely confirmed in recent years. After LH binds to its specific receptors on the membranes of the granulosa cells, the production of the natriuretic peptide and natriuretic peptide receptor is inhibited, but the secretion of the EGF-like growth factors is activated, which promotes the expansion of cumulus cells and oocyte maturation [5,6,7]. Our most recent study found that, in preovulation follicles, the highly expressed histone deacetyl transferase (HDAC3) inhibits the expression of the EGF-like factors, thus inhibiting oocyte maturation in mice. The increase in LH promotes oocyte maturation and cumulus cells’ expansion by downregulating HDAC3, and therefore upregulates the expression of EGF-like factors, especially amphiregulin (AREG), in the mouse [8]. The findings draw great interest in exploring if the rest of the HDACs are all active in improving female fertility. 

HDACs are epigenetic regulators that, together with histone acetyl transferases (HATs), regulate the histone acetylation balance in vivo through acetylation and deacetylation [9]. To date, 18 mammalian HDAC family members have been found. HDACs are divided into four groups according to the structural similarity with homologous proteins in yeast [10,11]. The enzyme activities of classes I (HDAC1, 2, 3 and 8), IIa (HDAC4, 5, 7 and 9), IIb (HDAC6 and 10) and IV (HDAC11) depend on Zn^2+^, while class III HDACs (also known as sirtuins) require NAD^+^ to exert their activity [12,13,14]. In our most recent studies, we showed that, in Tan sheep, HDAC6 within the ovarian granulosa cells was responsive to LH induction and induced the production of EGF-like growth factors and oocyte maturation [15]. Being among the first HDAC family members with HDAC3, HDAC1 can regulate gene transcription and cell apoptosis during mouse oocyte development, and plays an important regulatory role in early embryonic development after fertilization [16,17,18,19]. We therefore speculated that HDAC1 and HDAC3 may play similar roles in regulating the function of mammalian ovarian granulosa cells.

This study focused on one of the epigenetic modifying proteins, HDAC1, to investigate its effect on the secretion of EGF-like growth factors by ovarian granulosa cells and on oocyte maturation in Tan sheep, aiming to provide important theoretical support for the in vitro maturation of Tan sheep oocytes. 

## 2. Materials and Methods

### 2.1. Experimental Materials

The ovaries of Tan sheep were collected from Hongxiang slaughterhouse in Yongning County, Yinchuan City, Ningxia Hui Autonomous Region, China. The collected ovaries were quickly put into normal saline containing 1% penicillin and streptomycin at 37 °C and transported back to the laboratory within 2 h.

### 2.2. Methods

#### 2.2.1. Immunohistochemistry

Ovaries were fixed in cold 4% paraformaldehyde (Solarbio Company, Beijing, China, P1110) for 72 h. Then, the samples were dehydrated in ethanol and toluene, embedded in paraffin, and sectioned at a 6 μm thickness. The ovaries and follicles were transferred to 3-aminopropyl-triethoxysilane-treated microscope slides (Zhongshan Company, Beijing, China, ZLI-9001) for immunohistochemical staining. In brief, sections were deparaffinized and rehydrated, and antigen retrieval was performed by microwaving for 15 min in 0.01% sodium citrate buffer. The sections were then subjected to immunohistochemistry according to the instructions of the IHC Kit (Zhongshan Company, Beijing, China, PV-9001). The antibody complex was detected using DAB reagent according to the manufacturer’s instructions [20].

#### 2.2.2. Immunofluorescence

For immunofluorescence analysis, sections were blocked with 10% normal donkey (Zhongshan Company, Beijing, China, ZLI-9022) serum and incubated overnight at 4 °C with primary antibodies against HDAC1 before incubation with Alexa Fluor 488-conjugated secondary antibodies (1:100, Yeasen, Shanghai, China, 34206ES60) for 1 h at 37 °C and Hoechst 33,342 (Zhongshan Company, Beijing, China, C1028), as a nuclear counterstain. The samples were observed under a microscope.

#### 2.2.3. Follicle Dissection and Follicular Fluid Collection

The collected healthy ovine ovaries were put into 38 °C normal saline containing streptomycin and brought back to the laboratory within 2 h. The collected ovaries were washed once with 75% alcohol and then washed twice with PBS supplemented with penicptomycin (Cytiva, Logan, UT, USA, AH25722957). The excess ovarian tissue was removed with sterilized tweezers and scissors, and the follicles were separated from the ovaries using syringes. The follicles were divided into three categories: small (diameter, ≤2 mm), medium (diameter, 2–6 mm) and large (diameter, ≥6 mm). They were rinsed twice with PBS. A portion of the follicular fluid was stored at −80 °C for subsequent protein extraction. The remaining portion of the follicular fluid was extracted from the follicles using a syringe, and the collected follicular fluid was stored at −20 °C for subsequent experiments.

#### 2.2.4. Extraction and Culture of Primary Granulosa Cells

The granulosa cells were collected by piercing the follicle and centrifuging at 1000 r/min for 5 min. After centrifugation, the supernatant was discarded, and the cell pellet was resuspended by adding PBS, and washed repeatedly 3 times. DMEM/F12 (Cytiva, Logan, UT, USA, AG29855619) medium with 10% FBS (Gibco, CA, USA, 2045686CP) was added to the cell pellet, and the cells were then cultured in a cell incubator with 5% CO_2_ at 38.5 °C and saturated humidity. The growth of granulosa cells was observed under a microscope, and non-adherent cells and platelets were removed. Granulosa cells were cultured with different concentrations of pyroxamide (MCE, Shanghai, China, HY-13216), one of the specific inhibitors of HDAC1, and the appropriate and optimal concentrations of the inhibitor for subsequent experiments were determined.

#### 2.2.5. Oocyte Collection and Culture

Oocytes were obtained by the aspiration of follicles 2–5 mm in diameter. Oocytes with uniform cytoplasmic granules and coated with more than three layers of cumulus cells were co-cultured with granulosa cells for in vitro maturation (IVM). The basal medium was M199 (Gibco, CA, USA, 2239794) supplemented with 10% FBS and 1% penicillin–streptomycin (Gibco, CA, USA, 149237). They were divided into four groups according to the experimental design: basal medium + 2.5 IU of FSH (Sansheng Pharmaceutical Co., Ltd., Ningbo, China, 110254629) (negative control), basal medium + 2.5 IU of FSH + 5 IU of LH (Sansheng Pharmaceutical Co., Ltd., Ningbo, China, 110254634) (positive control), basal medium + 2.5 IU of FSH + 2.5 μM pyroxamide (experimental group 1) and basal medium + 2.5 IU of FSH + 5 IU of LH + 2.5 μM pyroxamide (experimental group 2). The results were observed after 24 h of incubation at 38.5 °C in an incubator with 5% CO_2_ and saturated humidity.

#### 2.2.6. Western Blotting

The granulosa cell precipitate was collected and centrifuged at 1000 r/min for 5 min; then, the supernatant was discarded, 50 μL of lysate (Kgi Technology Biology Co., LTD, Nanjing, China, KGP250) was added, the cells were lysed on ice for 30 min, and they were then centrifuged at 10,000 r/min for 10 min at 4 °C. After the lysate was added to the collected follicles, the follicles were broken using liquid nitrogen crushing apparatus and centrifuged at 10,000 r/min for 10 min at 4 °C [21]. The supernatant was collected, and the protein concentration was determined using a BCA Protein Quantitation Assay kit (Kgi Technology Biology Co., LTD, Nanjing, China, KGPBCA). The amount of protein to load was adjusted with 6× Protein Loading Buffer (TRAN, Beijing, China, P51114); then, the protein was boiled in water for 10 min and stored at −20 °C. An SDS-PAGE (Shanghai Yase Biotechnology Co., LTD, Shanghai, China, PG113) gel at an appropriate concentration was prepared, the samples were loaded and electrophoresed, and the proteins were then transferred to a PVDF membrane. After the proteins had been transferred to the PVDF membrane (Millipore, MA, USA, R1JB38278), the membrane was placed in a blocking solution containing 5% skim milk (BD, NJ, USA, 1053907) for 1 h at room temperature. The primary antibodies (HDAC1: Affinity, AF0178; AREG: ABmart, PU595967S; EREG:Abclonal, A16372; BTC:Affinity, DF7038; PCNA: Proteintech, 10205-2-AP; H3K9:abcam, ab32129; H3K14:abcam, ab52946; H4K12:abcam, ab177793; H4K6: abcam, ab109463; H4K5: abcam, ab51997; GAPDH: Proteintech, 10494-1-AP; Acetylated-Lysin: Cell Signaling, 9814) were incubated with the membrane at 4°C overnight. Then, it was washed in PBST (Tween 20: Solarbio, T8220, PBS: Servicebio, G0002) 3 times, for 5 min each time. The membranes were incubated with horseradish peroxidase-conjugated secondary antibodies (Proteintech, Chicago, USA, SA00001-1/SA00001-2); then, they were washed in PBST 3 times, for 5 min each time. The ECL (Thermo Fisher Scientific, CA, USA, XA338899) chemiluminescence reagent was prepared and developed. The ImageJ 1.8.0 software was used to analyze the gray values of the Western blotting bands.

#### 2.2.7. The Criteria for Expansion of Cumulus Cells

The expansion of cumulus cells was evaluated under a light microscope after 24 h of COC culture in vitro. The evaluation was performed according to objective criteria: the presence of cumulus cells with no expansion was defined as grade 0; slight expansion of the outermost layer was grade 1; the spread of only the outermost cumulus cells was defined as grade 2; the expansion of all cells except the corona radiata was grade 3; the presence of expansion in all the cumulus cell layers was defined as grade 4 [22].

#### 2.2.8. Enzyme-Linked Immunosorbent Assay (ELISA)

EGF-like growth factors in the follicular fluid and granulosa cell secretory fluid were detected using an ELISA kit (Shanghai Enzyme-Linked Biotechnology Co., LTD, Shanghai, China, YJ112581) [20]. The detection was performed according to the kit’s instructions. In brief, the samples were added to the bottom of plate wells and incubated at 37 °C for 30 min. Each well was filled with washing solution, the plates were stood for 30 s, and then, the washing solution was discarded; this was repeated 5 times. HRP-conjugated reagent (50 μL) was added to each well except the blank well, and the plates were incubated at 37 °C for 30 min. Each well was filled with washing solution, and the plates were stood for 30 s; then, the solution was discarded; this was repeated 5 times. Chromogenic agent A (50 μL) was added to each well; then, chromogenic agent B (50 μL) was added. The mixture was gently shook and mixed at 37 °C, avoiding light, for 10 min. The reaction was terminated by adding 50 μL of stop solution. The blank wells were zeroed, and the absorbance of each well was measured sequentially at a 450 nm wavelength.

#### 2.2.9. Cellular Immunofluorescence

Granulosa cells were cultured for 24 h, the culture medium was aspirated, and the cells were washed with PBS 3 times, for 3 min each time. A 4% fixative solution was added for fixation at room temperature for 20 min, and the cells were washed with PBS 3 times, for 3 min each time. After adding 0.5% TritonX-100 (Sigma-Aldrich, St. Louis, MO, USA, SLBV4122) and permeating the cells at room temperature for 20 min, the cells were washed with PBS 3 times, for 3 min each time. A 10% donkey serum blocking solution was added for blocking for 30 min at room temperature. The blocking solution was removed by suction, and the primary antibody was similarly incubated with the cells, followed by washing with PBS 3 times, for 3 min each time. Fluorescent secondary antibody was added, and the samples were incubated at 37 °C for 1 h. The samples were washed with PBS 3 times, for 3 min each time. A laser confocal microscope was used to observe and photograph the film after sealing.

#### 2.2.10. Statistical Analysis

Statistical analyses were performed using the GraphPad Prism 6.0 software. SPSS Statistics 22.0 was used for Pearson coefficient analysis. The experimental results were obtained from at least three biological repeats. Comparisons between experimental groups were conducted using a one-way analysis of variance with Tukey–Kramer multiple-comparison tests. The experimental results are expressed as the mean ± standard error of the mean (SEM, GraphPad Software, La Jolla, CA, USA). Statistical significance was defined as a *p* value < 0.05. 

## 3. Results

### 3.1. HDAC1 Expression in the Growing Follicles Resided in the Nuclei of Both Germ Cells and Ovarian Somatic Cells in the Tan Sheep

**Figure 1:**
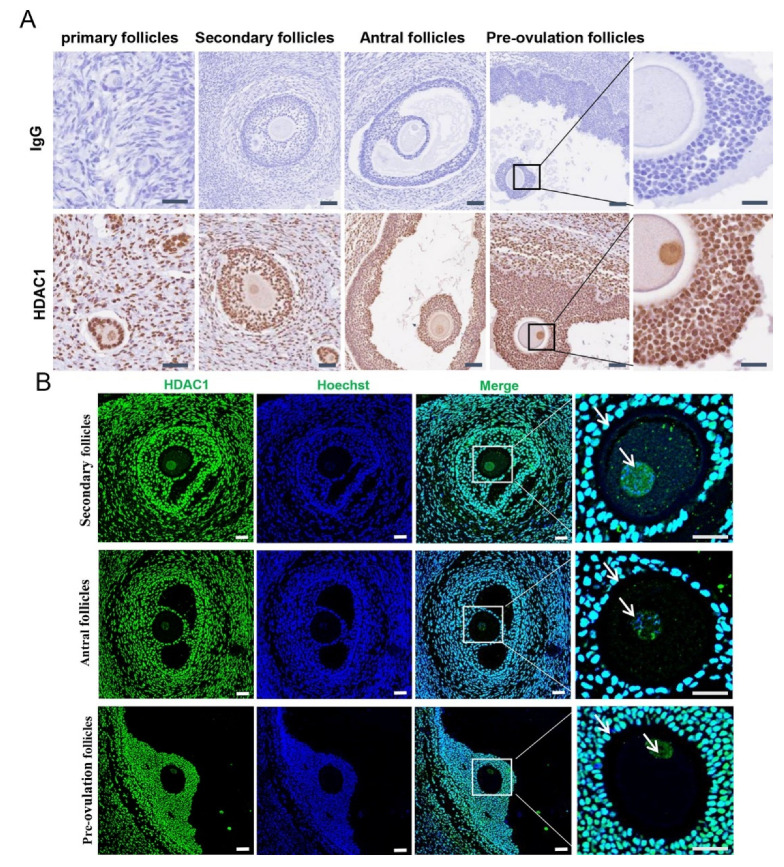
Localization and expression pattern of HDAC1 in the ovary of Tan sheep. (**A**) Immunohistochemistry. The scale was 200 μm. The image in the far-right column is a magnified view of the pre-ovulation follicle. (**B**) Immunofluorescence. Note: Green fluorescence indicates HDAC1, and blue fluorescence indicates Hoechst staining to mark the nucleus. The scale was 200 μm; the rightmost column contains an enlarged view of the merged picture.

To identify the location of HDAC1 in the ovaries of Tan sheep, immunohistochemistry and immunofluorescence assays were applied. The results showed that HDAC1 was expressed in the nuclei of both the granulosa and the oocytes in growing follicles including primary follicles, secondary follicles, antral follicles and pre-ovulatory follicles (Figure 1A,B). 

### 3.2. The HDAC1 Expression Was Dependent on Follicular Growth in Freshly Collected Follicles of the Tan Sheep

**Figure 2:**
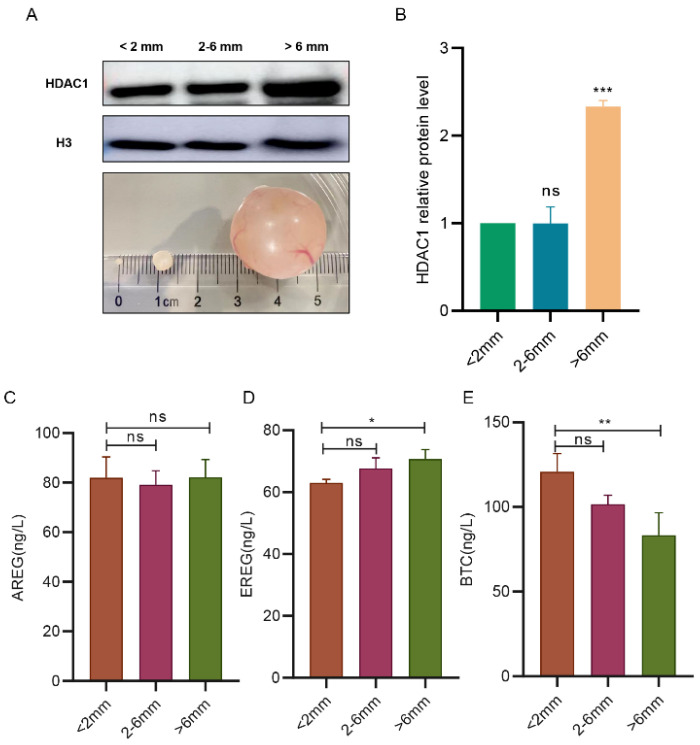
The expression level of HDAC1 and the secretion levels of EGF-like growth factors, including AREG, EREG and BTC, in the follicles at different developmental stages. (**A**,**B**): The expression levels of the HDAC1 protein in the follicles were analyzed by Western blotting. Stripped follicles, from left to right: small antral follicle (diameter, <2 mm), medium antral follicle (diameter, 2–6 mm) and large antral follicle (diameter, >6 mm). (**C**–**E**): The levels of AREG, EREG and BTC in the follicles at different developmental stages were examined through ELISA. * p < 0.05; ** *p* < 0.01; *** *p* < 0.001; ns: Indicate no significant differences, *n* = 3.

In order to explore the expression pattern and dynamic characteristics of HDAC1 in the growing follicles of Tan sheep, we isolated and divided the follicles into three groups: small antral follicles, smaller than 2 mm; medium antral follicles, 2–6 mm; and large antral follicles, larger than 6 mm. Then, Western blotting was used to detect the expression of the HDAC1 protein in these follicles. The results showed that the expression of HDAC1 was significantly upregulated when the follicle was larger than 6 mm (Figure 2A,B) (*p* < 0.001). According to the statistical results, we found that the maximum expression level was 2.3 times the minimum expression level. 

To examine the concentrations of the respective EGF-like growth factors such as AREG, epithelial regulatory protein (EREG) and betacellulin (BTC) within the follicular fluid of developmental follicles, fluids from the three groups of follicles that had been classified were collected (the follicle classification is shown in Figure 2A), and then examined by the ELISA. The results showed that the levels of the three factors were inconsistent with each other. Briefly, the level of EREG was upregulated, while the level of BTC was downregulated, with follicles < 2 mm developing to those > 6 mm, respectively (Figure 2C–E) (*p* < 0.05, *p* < 0.01). We did not observe significant changes in the level of AREG.

### 3.3. Inhibition of HDAC1 in Granulosa Cells Had Mild Influence on Production of EGF-like Growth Factors 

**Figure 3:**
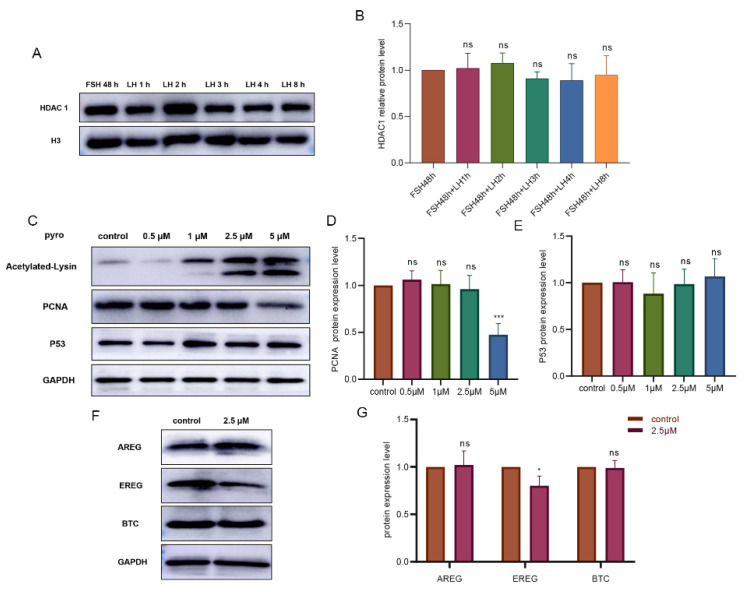

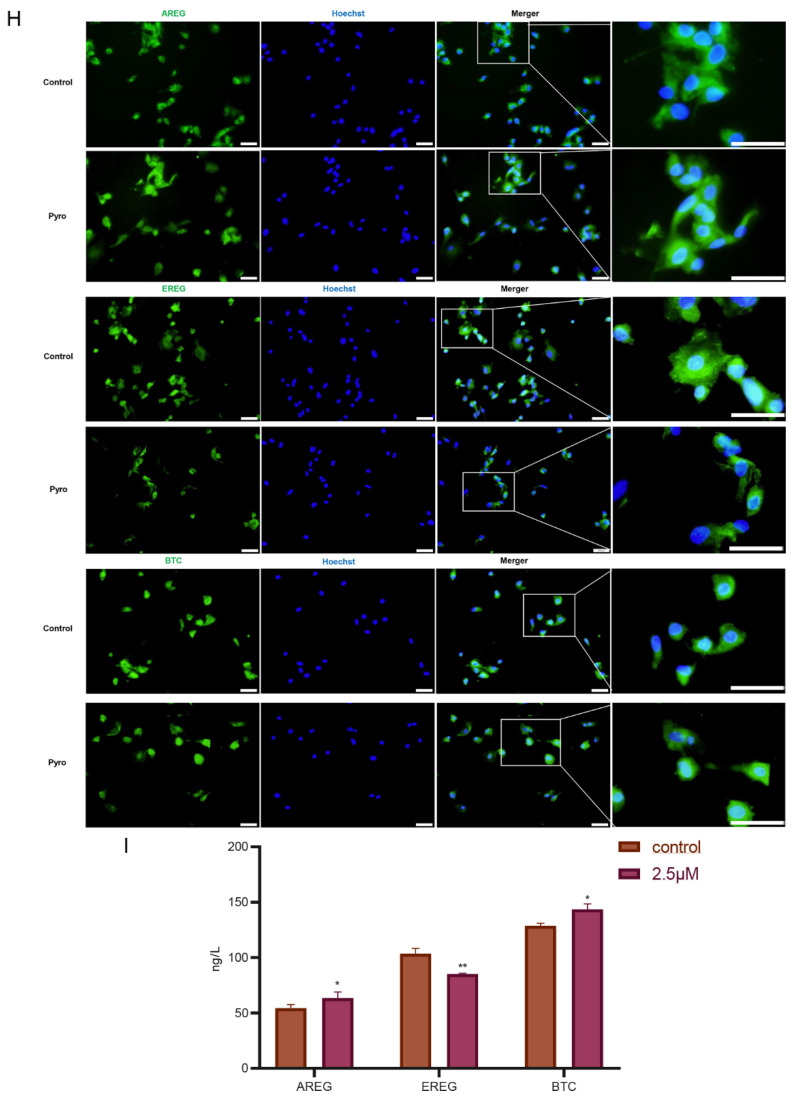
The effect of HDAC1 inhibition on the expression and secretion of EGF-like growth factors in cultured granulosa cells from Tan sheep. (**A**,**B**): Analysis of the effect of the LH time on HDAC1 protein expression. (**C**–**E**): Screening the inhibitory efficiency of pyroxamide, a specific inhibitor of HDAC1, regarding the levels of proteins, as indicated by acetylated lysin. (**F**,**G**): The expression levels of the EGF-like growth factors in response to pyroxamide treatment. (**H**): The expression levels of the respective EGF-like growth factors detected by immunofluorescence. The green fluorescence indicated AREG, EREG and BTC, and the blue fluorescence indicated Hoechst staining to mark the nucleus. The scale was 100 μm; the rightmost column contains an enlarged view of the merged figure. (**I**): The respective levels of the secreted AREG, EREG and BTC in the cultured granulosa cells of the Tan sheep. * p < 0.05; ** p < 0.01; *** p < 0.001; ns: Indicate no significant differences, *n* = 3.

Primary granulosa cells cultured in vitro were treated with follicle-stimulating hormone (FSH) for 48 h and then treated with LH for 1, 2, 3 and 4 h, respectively. The cultured granulosa cells were collected and tested by Western blotting. We found that there was no significant change in HDAC1 with an increase in LH action time (Figure 3A,B). To clarify the role of HDAC1 in the improvement of oocyte maturation, pyroxamide (hereinafter referred to as pyro), a specific inhibitor of HDAC1, was used in our granulosa cell in vitro culture model. Firstly, the optimal concentration of the inhibitor was determined. The appropriate concentration of the HDAC1 inhibitor was 2.5 μM, which was confirmed by detecting the levels of pan-acetyl within the examined granulosa cells, the proliferating cell nuclear antigen (PCNA), and the tumor protein p53 (P53) (Figure 3C–E).

The changes in EGF-like growth factors in response to pyro were examined using cultured granulosa cells. It was found that the expression of the EREG protein was downregulated by pyro, while the levels of both the AREG and BTC proteins were unchanged (Figure 3F,G) (*p* < 0.05). The immunofluorescence results also showed that the fluorescence expression intensity of EREG was significantly lower than that of the control group, while AREG and BTC were unaffected by the pyro (Figure 3H). Subsequently, we collected the supernatant of the cultured granulosa cells and detected the levels of the respective proteins in the media, which showed that the inhibition of HDAC1 reduced the level of the secreted EREG in the supernatant, whereas the levels of both AREG and BTC were slightly increased (Figure 3I) (*p*< 0.01).

### 3.4. HDAC1 Was Indispensable for Cumulus Cell Expansion and Oocyte Maturation for the Tan Sheep In Vitro

To investigate whether HDAC1 regulated oocyte maturation by mediating granulosa cells’ secretion of EGF-like factors, the effect of granulosa cells co-cultured with COCs in vitro was established according to published protocols [15,23]. The study included the following groups: FSH (negative control), FSH+LH (positive control), FSH + 2.5 μM Pyro (experimental group 1) and FSH + LH + 2.5 μM Pyro (experimental group 2).

**Figure 4:**
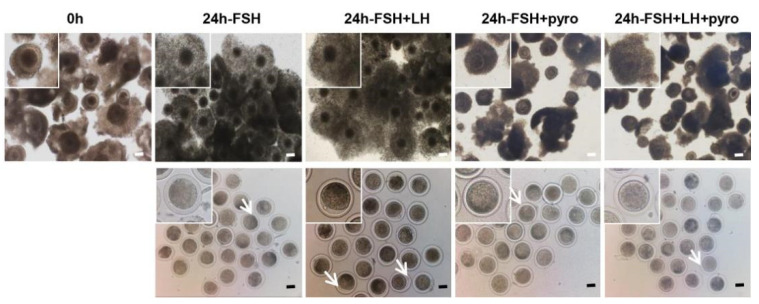
Inhibition of HDAC1 by pyroxamide affected the oocyte maturation in the in vitro-cultured model of COCs of Tan sheep. Schematic diagram of cumulus expansion and the first polar body. Note: Pyroxamide: HDAC1-specific inhibitor; 200 μm scale. White arrows indicate the extrusion of the first polar body in the respective groups. *n* = 3.

In order to evaluate the effect of oocyte maturation, both the cumulus cells’ expansion and the oocyte extrusion of the first polar body (PB1) were explored. The results showed that the cumulus expansion of COCs in both the experimental groups (FSH + Pyro and FSH + LH + Pyro) was significantly inhibited (Figure 4). To further analyze the effect, the cumulus expansion was judged according to the reported standard, which divides the expansion into five grades: 0, 1, 2, 3 and 4. The cumulus expansion rate of the COCs in the FSH + Pyro group was 7.63%, and that in the FSH + LH + Pyro group was 6.25%; both were significantly lower than those in the negative-control group (94.07%) and the positive-control group (94.43%) (*p* < 0.001) (Table 1). The results imply that the inhibition of HDAC1 in vitro prevents the expansion of cultured COCs from Tan sheep.

Furthermore, the maturation rate of the cultured COCs was calculated by quantifying the extrusion of the first polar body. The results showed that the respective oocyte maturation rate of the FSH group was 29.4%, that of the FSH + LH group was 56.1%, that of the FSH + 2.5 μM Pyro group was 29.25% and that of the FSH + LH + 2.5 μM Pyro group was 31.6%. Statistical analysis showed that there was no significant difference in the oocyte maturation rate between the experimental groups (FSH + 2.5 μM Pyro and FSH + LH + 2.5 μM Pyro) and the negative-control group (FSH). However, significant differences between the two experimental groups and the positive-control group (FSH + LH) were observed (*p* < 0.001) (Table 2). These results indicate that the inhibition of HDAC1 in vitro not only inhibits the expansion of cumulus cells of COCs, but also inhibits the oocyte maturation of COCs.

### 3.5. Inhibition of HDAC1 Affects Multiple Acetylation Sites in the Granulosa Cells

**Figure 5:**
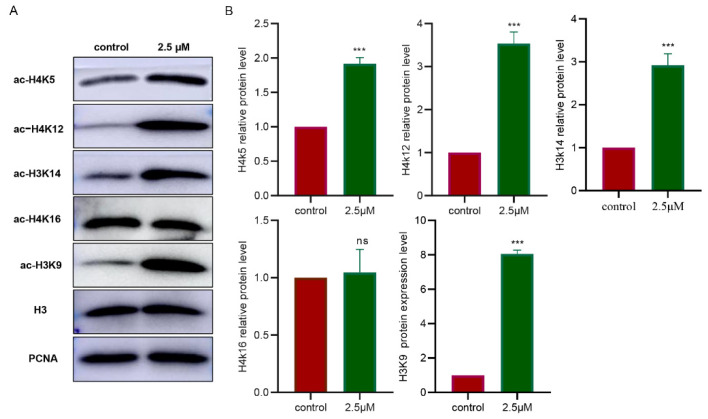
Inhibition of HDAC1 affected the expression of acetylated histones in the cultured granulosa cells. (**A**,**B**): Changes in acetylation sites of different histones detected by Western blotting assays. *** p < 0.001. Pyroxamide: specific inhibitor of HDAC1. *n* = 3. ns: Indicate no significant differences.

To clarify which acetylation sites in the granulosa cells of the Tan sheep HDAC1 affected, and which acetylation sites were involved in the secretion of EGF-like growth factors, the acetylation data for the sites including H4K5, H4K12, H3K14 H3K9 and H4K16 were examined. The results showed that the levels of the acetylation of the histones in the sites of H4K5, H4K12, H3K14 and H3K9, except for H3K16, were significantly upregulated (*p* < 0.001) (Figure 5A,B). According to the Pearson correlation analysis, H4K5 and H4K12 were strongly positively correlated with AREG, while H3K14, H3K9 and H4K16 were not correlated with AREG (Table S1). Additionally, H4K5, H4K12, H3K14 and H3K9 were extremely strongly negatively correlated with EREG (Table S2). It also showed that H4K5, H4K12, H3K14 and H3K9 were extremely strongly positively correlated with BTC, but H4K16 was not correlated with EREG and BTC (Table S3).

## 4. Discussion

The importance of HDACs acting as epigenetic modifiers in biological processes, including the regulation of transcription and translation, protein stability and protein localization, has been proven [24,25,26]. Among the HDACs, HDAC1 is widely distributed in various tissues, mainly localized in the nucleus, functioning in suppressing gene transcription [27,28]. Studies have shown that HDACs play an important role in oocyte development as epigenetic modifying proteins [29,30,31]. Interestingly, we proved here that HDAC1 is indispensable for supporting cumulus expansion and oocyte meiotic maturation in vitro in Tan sheep through regulating multiple acetylation sites of the histones. However, unlike HDAC3 in mice [8] and HDAC6 in sheep, HDAC1 is possibly not one of the classic LH signal response molecules in the granulosa cells and may exert its action independently of the EGF-like factors in Tan sheep [15].

In this study, HDAC1 was found to be specifically localized in the parietal granulosa nuclei, cumulus nuclei and oocyte nuclei of growing follicles, indicating that HDAC1 may be involved in the development of follicles in the ovaries of Tan sheep. According to a previous study, HDAC1 was expressed in both oocytes and in vitro-fertilized embryos, and a decrease in the expression of HDAC1 in oocytes and embryos may have a negative impact on embryo development [32]. However, in this study, the continuously increased level of HDAC1 in these growing follicles implies that HDAC1 may be pivotal for the growth of the follicles. Additionally, the levels of multiple histone acetylation sites were affected by HDAC1 inhibition, implying that systemic gene expression may be dependent on the continuously expressed HDAC1 under physiological conditions.

Unlike other HDACs—such as HDAC6, which is a downstream signal that triggers the expression of EGF-like growth factors in Tan sheep in response to LH induction—HDAC1 seems to be unique in that it may contribute to oocyte maturation and cumulus cell expansion independently of EGF-like growth factors [33,34,35,36]. In a physiological situation, HDAC6 is expressed in both the granulosa cells and the oocytes of the ovarian follicles of Tan sheep. HDAC6 decreases time dependently with follicular development [15]. In mice, the secretion of AREG, EREG and BTC is controlled by HDAC3. With a surge in LH, the decrease in HDAC3 in granulosa cells enables histone H3K14 acetylation and the binding of the SP1 transcription factor to the AREG promoter, initiating AREG transcription and oocyte maturation [8]. In our study, the secretion of EGF-like growth factors (EREG and BTC) was found to change under physiological conditions, indicating that the EGF-like growth factors of EREG and BTC play important roles in the maturation of Tan sheep oocytes. However, EGF-like growth factors seem to not be the key factors affected by HDAC1 in the ovarian granulosa cells of the Tan sheep. On the one hand, the levels of these growth factors in the cultured granulosa cells are mildly affected after HDAC1 is inhibited. On the other hand, LH induction did not affect the level of the HDAC1 protein in the cultured granulosa cells. Therefore, we speculated that HDAC1 may regulate cumulus expansion and oocyte maturation not through the classical LH–EGF-like growth factor signaling pathway but through other signaling pathways. Interestingly, the inhibition of HDAC1 significantly reduced the promoting effect of LH on cumulus expansion and oocyte nucleic maturation. Therefore, a constant level of HDAC1 in the preovulatory follicle may be pivotal for supporting the oocyte maturation process independently of the EGF-like factors. Additionally, HDAC1 is not responsive to LH induction. Further studies using oocyte and granulosa cells separately are needed to fully address this point.

The acetylation of the histones in the germ cells changed dynamically in the sheep. In sheep oocytes, for example, H3K9 and H4K12 were acetylated from the germinal vesicle (GV) to the GV breakdown stage, and then deacetylated at the metaphase I (MI) stage; reacetylation was observed from the anaphase I (AI) to metaphase II (MII) stage [37,38]. Additionally, the acetylation of H4K5 first appeared in MI sheep oocytes and became intensive at the AI stage [37,38]. These observations are different from those for the mice. When HDAC1 was knocked down in the oocytes, it resulted in the hyperacetylation of the histone H4 [39], which did not affect the maturation rate or cleavage of parthenogenetic embryos. However, it led to a decrease in the blastocyst rate. Furthermore, the acetylation of the histone H3K14 in parthenogenetic embryos increased after HDAC1 knockdown [40]. After systematically analyzing the changes in the acetylation sites controlled by HDAC1 in sheep granulosa cells, we found that the expression of H4K5, H4K12, H3K14 and H3K9 was upregulated, but that of H4K16 was not. Our correlation analysis showed that parts of the acetylation sites are positively correlated with EGF-like factors, implying that HDAC1 may be indirectly related to the physiological function of EGF-like growth factors. The complex relationship between them requires substantial research.

## 5. Conclusions

This study provides evidence that the expression and localization of HDAC1 in ovarian granulosa cells are constant and dependent on the follicle developmental stage in Tan sheep. Based on our in vitro inhibition models with granulosa culture and COC culture, HDAC1 was proven to be essential for cumulus expansion and oocyte maturation, but the action of HDAC1 is independent of EGF-like growth factors, unlike the actions of other HDACs that have been studied in sheep. In conclusion, new light has been shed on the understanding of epigenetic influences on follicle development and gonadotrophin-induced oocyte maturation in Tan sheep. 

## Figures and Tables

**Table 1:** Effect of the HDAC1 inhibitor on the cumulus expansion of oocytes (*n* = 3).

Group	FSH	FSH + LH	FSH + Pyroxamide	FSH + LH + Pyroxamide
0–2 level (%)	5.93±2.53	5.24±2.01 ^ns^	92.36±2.23 ***	93.75±2.41 ***
3–4 level (%)	94.07±2.6	94.43±2.37 ^ns^	7.636±2.24 ***	6.25±2.41 ***

*** *p* < 0.001 vs. FSH group, ns: Indicate no significant differences vs. FSH group.

**Table 2:** Effect of HDAC1 inhibitor on oocyte maturation (*n* = 4).

Group	Number of First Polar Body Emission (Pieces)	Total Number of Oocytes (Pieces)	First Polar Body Discharge Rate (%)
FSH	43	143	29.4 ± 6.3
FSH + LH	84	148	56.1 ± 4.8 ***
FSH + pyroxamide	38	129	29.25 ± 6.65 ^ns^
FSH + LH + pyroxamide	41	130	31.6 ± 5 ^ns^

***p<0.001 vs. FSH group, ns: Indicate no significant differences vs. FSH group.

## Data Availability

The data presented in this study are available on request from the corresponding author.

## Supplementary Materials

The following supporting information can be downloaded at: https://www.mdpi.com/article/10.3390/biology11101464/s1. Table S1: Analysis of the correlation between acetylation sites and AREG; Table S2: Analysis of the correlation between acetylation sites and EREG; Table S3: Analysis of the correlation between acetylation sites and BTC.

## Abbreviation

English Abbreviation	Full English Name
HDACs	Histone deacetylases
HDAC1	Histone deacetylase 1
HAT	Histone acetyltransferase
EGF	Epidermal growth factor
AREG	Amphiregulin
EREG	Epithelial regulatory protein
BTC	Betacellulin
FSH	Follicle-stimulating hormone
LH	Luteinizing hormone
COCs	Cumulus oocyte complexes
PBS	Phosphate-buffered saline
FBS	Fetal bovine serum
PB1	First polar body
ELISA	Enzyme-linked immunosorbent assay
PCNA	Proliferating cell nuclear antigen
P53	Tumor protein p53
PVDF	Polyvinylidene fluoride
GV	Germinal vesicle
AI	Anaphase I
MI	Metaphase I
MII	Metaphase II
IVM	In vitro maturation
