# Supplementary material for: HDAC1 in the Ovarian Granulosa Cells of Tan Sheep Improves Cumulus Cell Expansion and Oocyte Maturation Independently of the EGF-like Growth Factors
Source: Biology (Basel). 2022 Oct 6;11(10):1464. doi: 10.3390/biology11101464 (PMC9598242; doi:10.3390/biology11101464)
Supplement: Supplementary file 1 [file biology-11-01464-s001.zip › Supplementary Materials Table S1-S3.pdf]

Table S1. Correlation analysis between acetylation sites and AREG.

Acetylation sites	Pearson correlation coefficient（r）	The related degree
H4K5	0.824	Strong positive correlation
H4K12	0.833	Strong positive correlation
H3K14	0.785	No correlation
H3K9	0.810	No correlation
H4K16	0.303	No correlation

Table S2. Correlation analysis between acetylation sites and EREG.

Acetylation sites	Pearson correlation coefficient（r）	The related degree
H4K5	-0.983	Extremely strong negatively correlation
H4K12	-0.969	Extremely strong negatively correlation
H3K14	-0.984	Extremely strong negatively correlation
H3K9	-0.991	Extremely strong negatively correlation
H4K16	-0.338	No correlation

Table S3. Correlation analysis between acetylation sites and BTC.

Acetylation sites	Pearson correlation coefficient（r）	The related degree
H4K5	0.948	Extremely strong positive correlation
H4K12	0.954	Extremely strong positive correlation
H3K14	0.940	Extremely strong positive correlation
H3K9	0.963	Extremely strong positive correlation
H4K16	0.491	No correlation
