# Supplementary figures and images for: HDAC1 in the Ovarian Granulosa Cells of Tan Sheep Improves Cumulus Cell Expansion and Oocyte Maturation Independently of the EGF-like Growth Factors
Source: Biology (Basel). 2022 Oct 6;11(10):1464. doi: 10.3390/biology11101464 (PMC9598242; doi:10.3390/biology11101464)

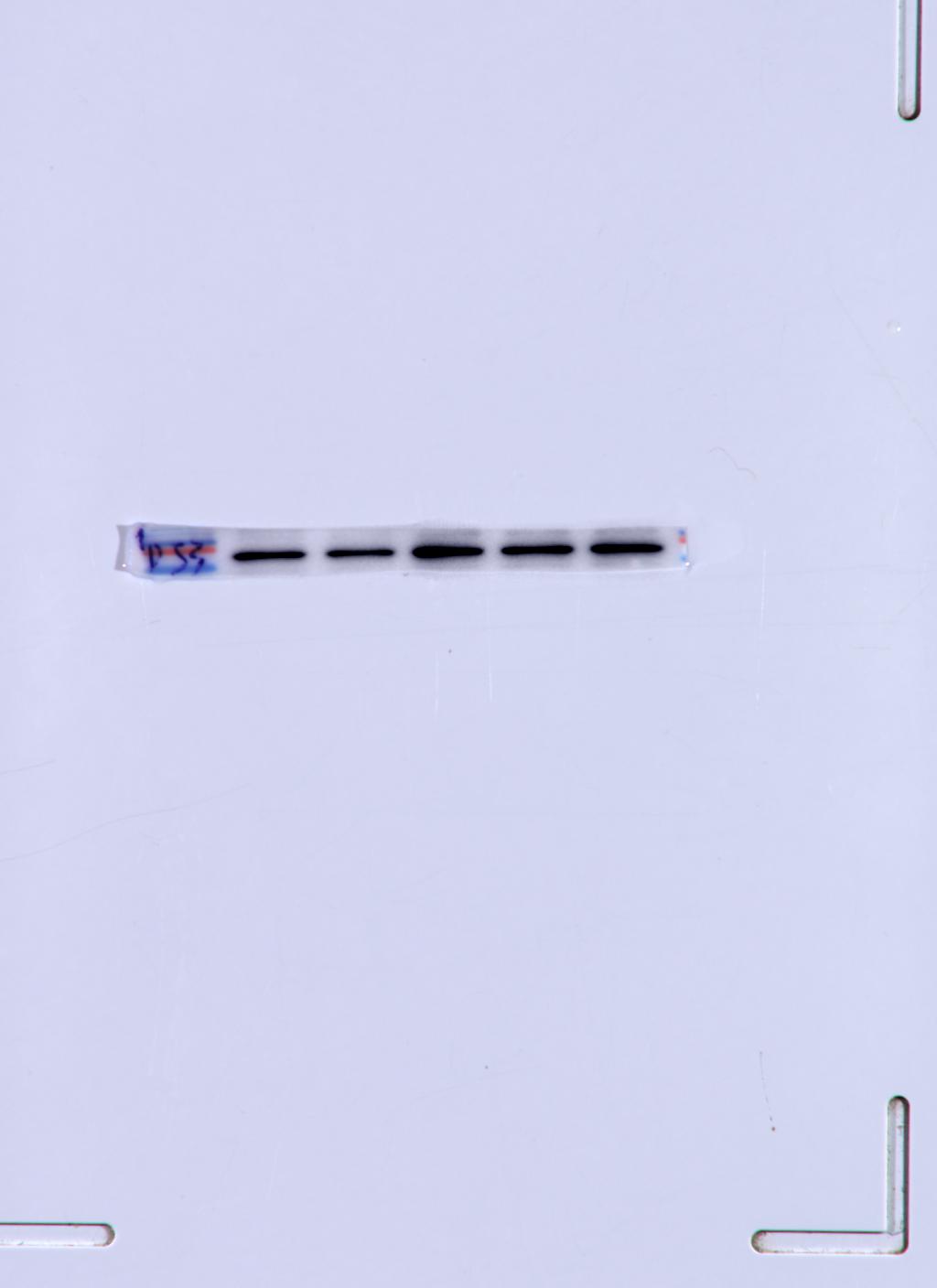

Supplement: Supplementary file 1 [file biology-11-01464-s001.zip › WB FIG/AC/h1-53 2022.05.10_12.07.20_Ch+Marker.jpg]

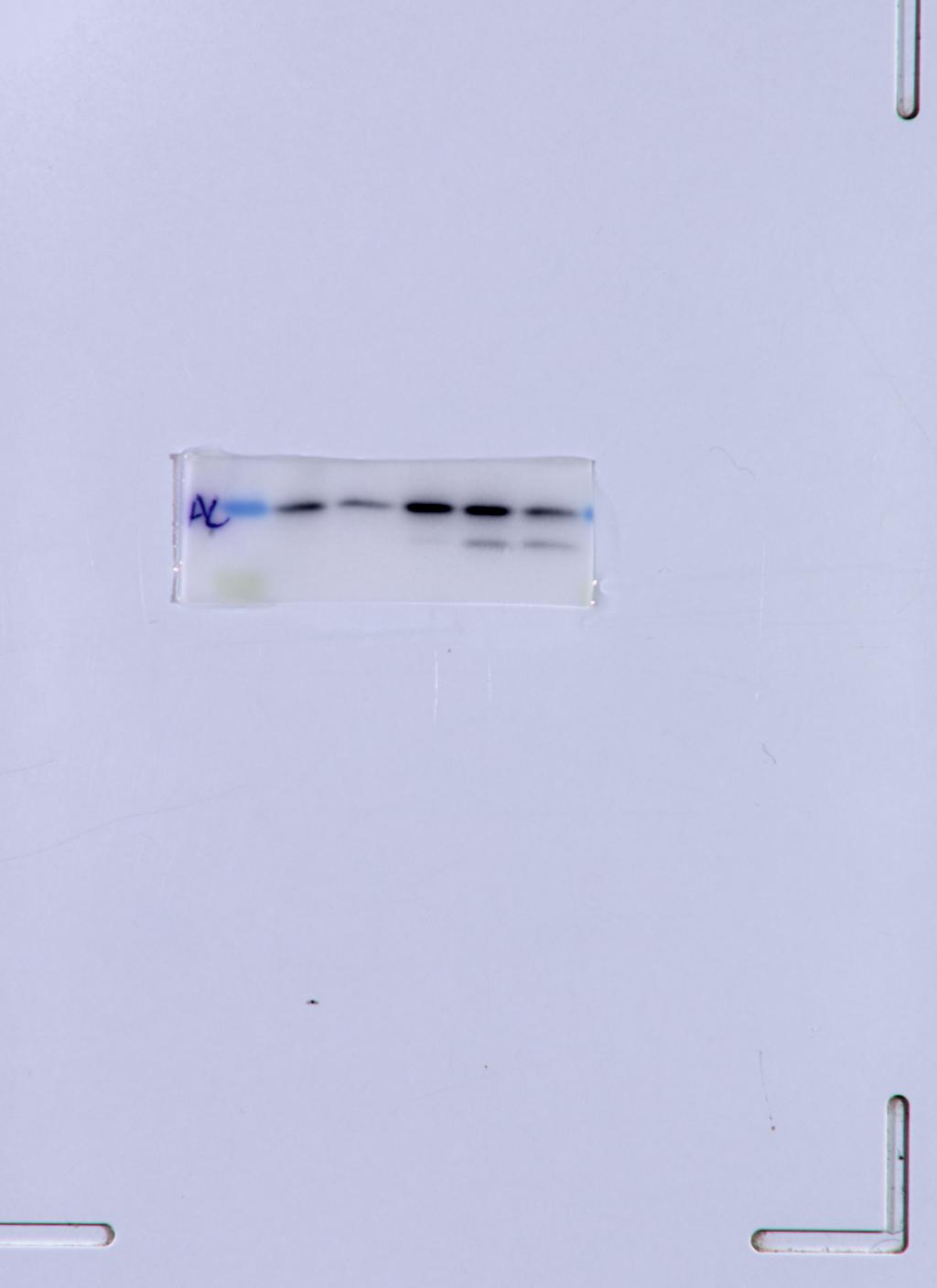

Supplement: Supplementary file 1 [file biology-11-01464-s001.zip › WB FIG/AC/h1-fanyx 2022.05.05_16.19.07_Ch+Marker.jpg]

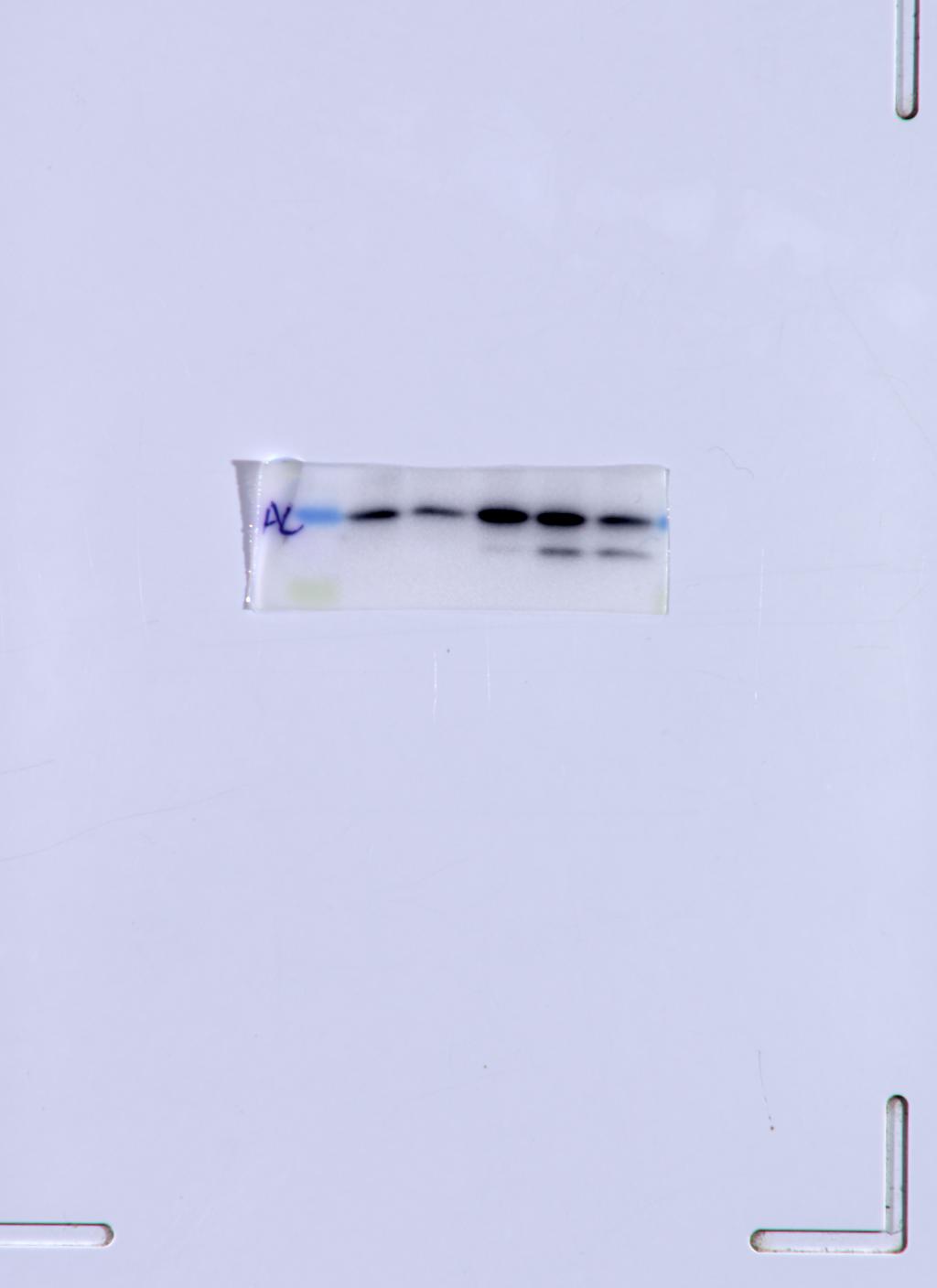

Supplement: Supplementary file 1 [file biology-11-01464-s001.zip › WB FIG/AC/h1-fanyx 2022.05.05_16.41.18_Ch+Marker.jpg]

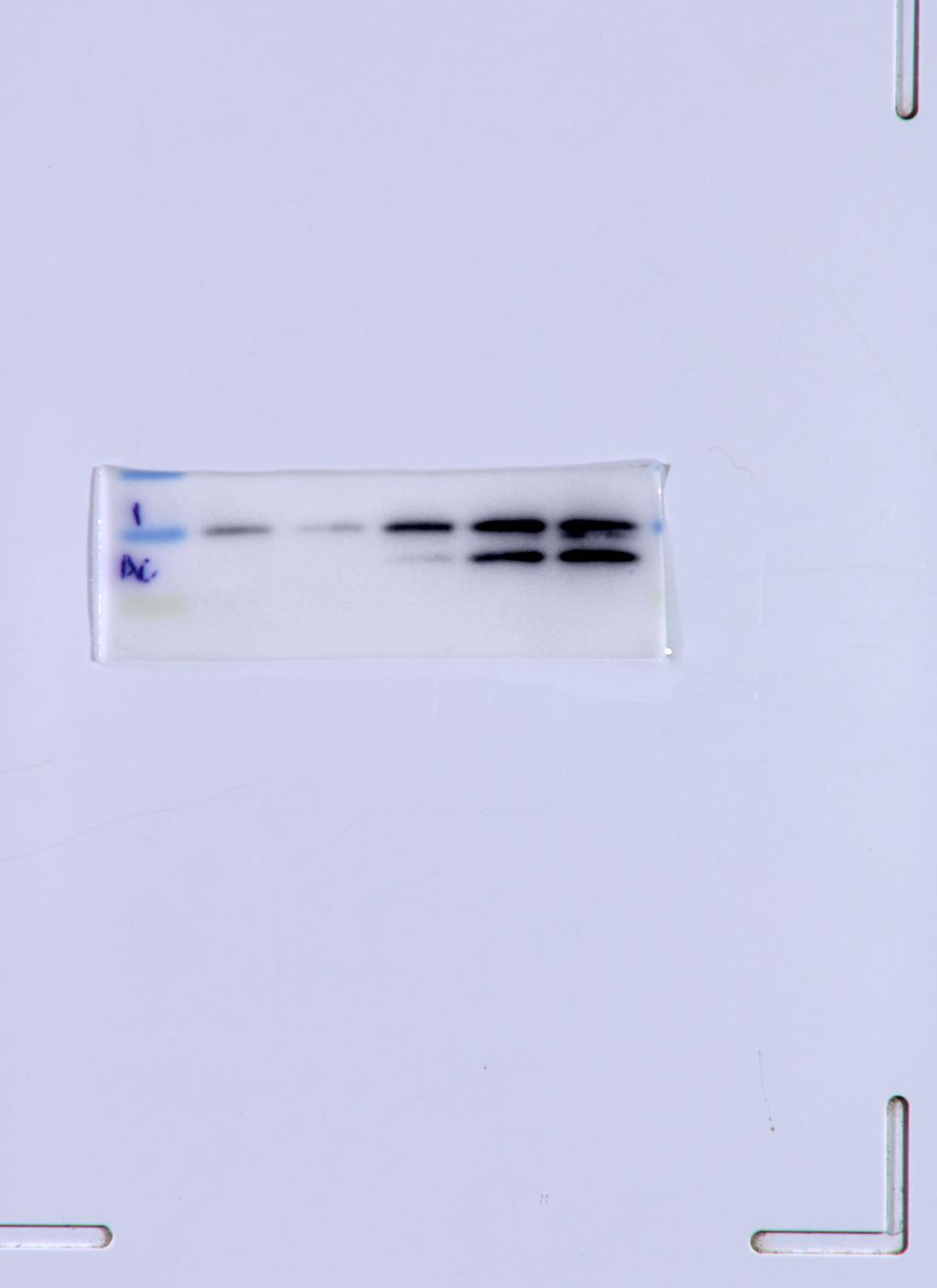

Supplement: Supplementary file 1 [file biology-11-01464-s001.zip › WB FIG/AC/h1-fanyx 2022.05.10_12.17.50_Ch+Marker.jpg]

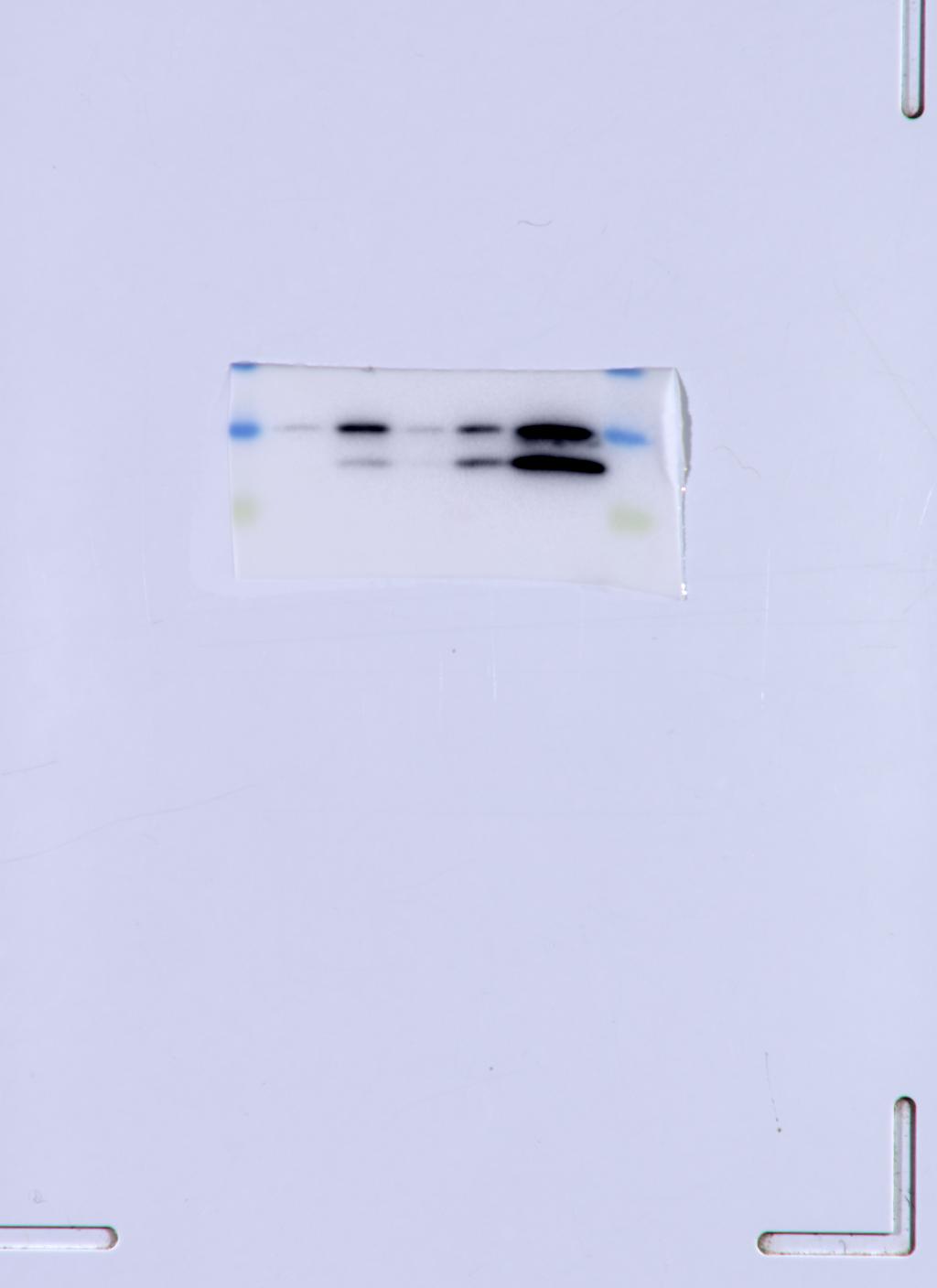

Supplement: Supplementary file 1 [file biology-11-01464-s001.zip › WB FIG/AC/h1-fanyx 2022.05.12_18.49.24_Ch+Marker.jpg]

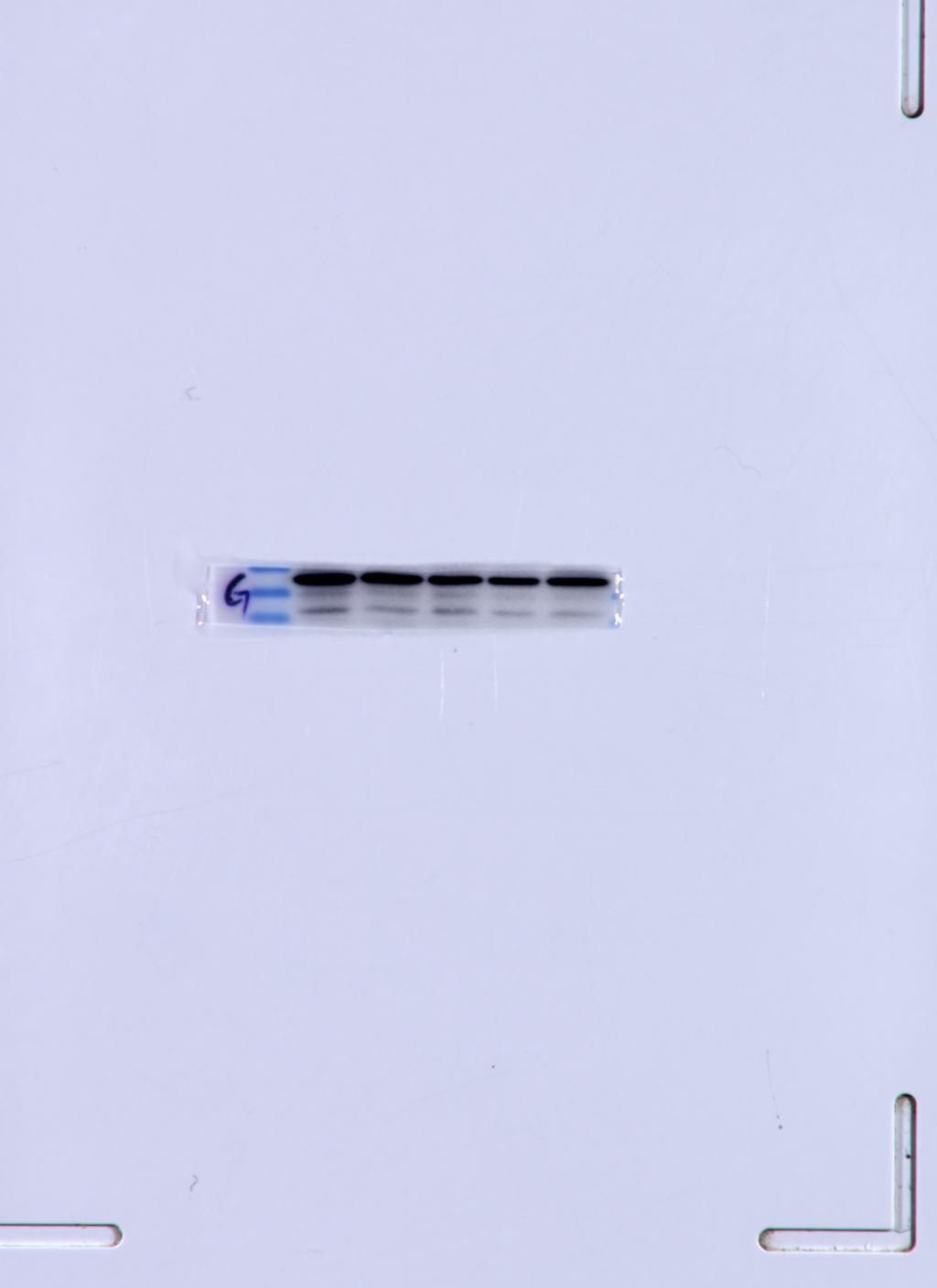

Supplement: Supplementary file 1 [file biology-11-01464-s001.zip › WB FIG/AC/h1-gap 2022.05.05_16.13.36_Ch+Marker.jpg]

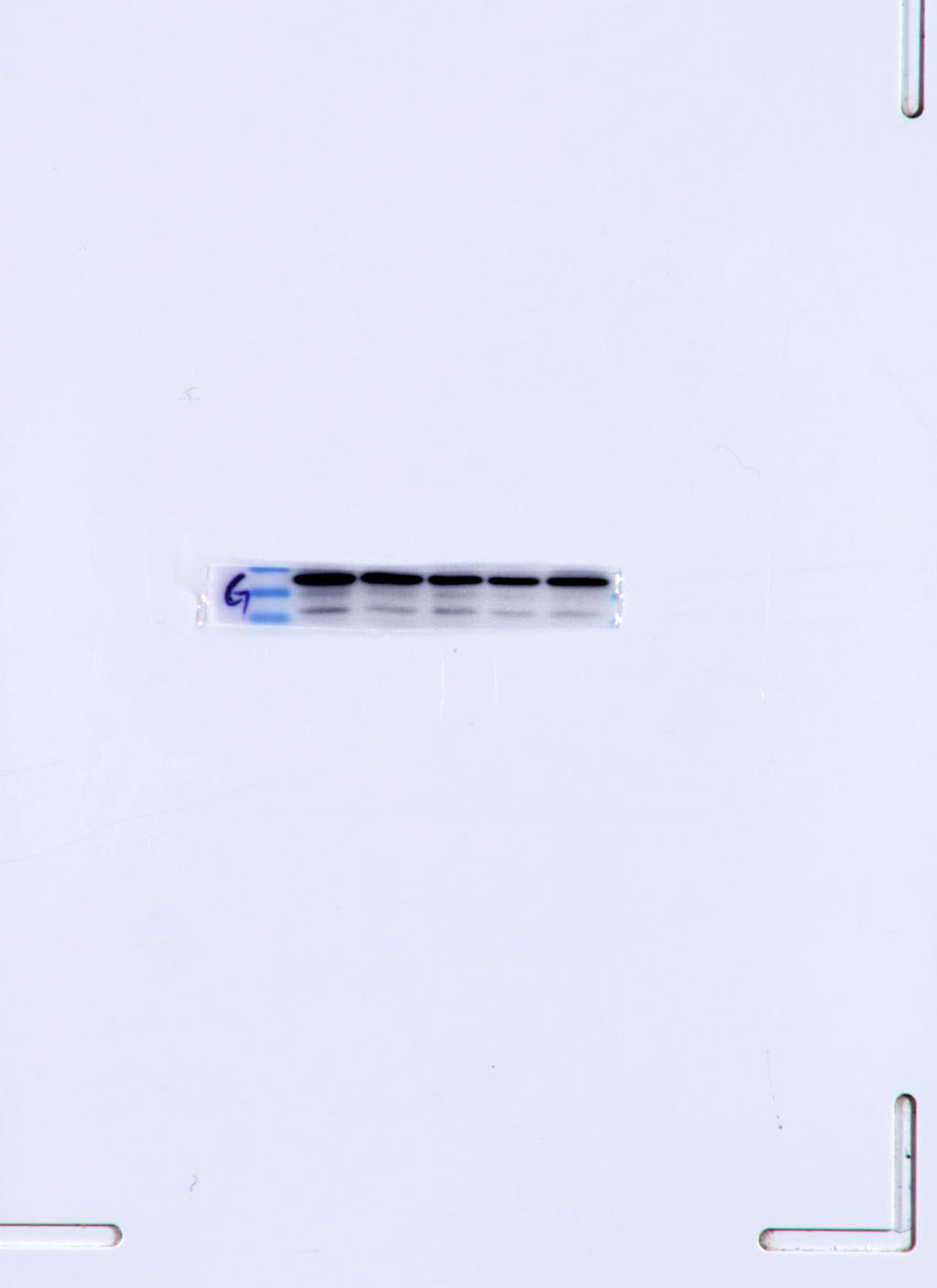

Supplement: Supplementary file 1 [file biology-11-01464-s001.zip › WB FIG/AC/h1-gap 2022.05.05_p.jpg]

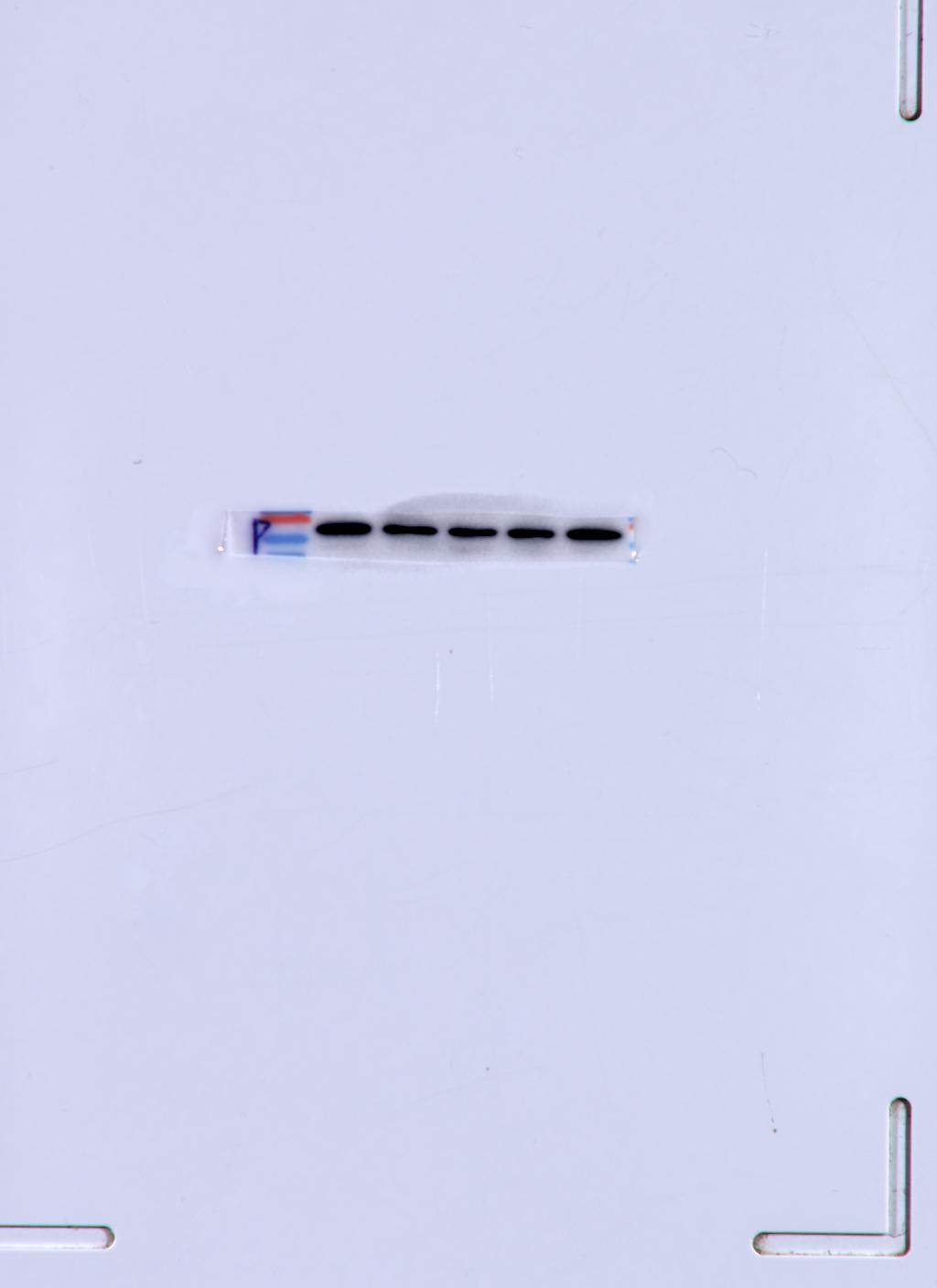

Supplement: Supplementary file 1 [file biology-11-01464-s001.zip › WB FIG/AC/h1-p53 2022.05.05_16.25.01_Ch+Marker.jpg]

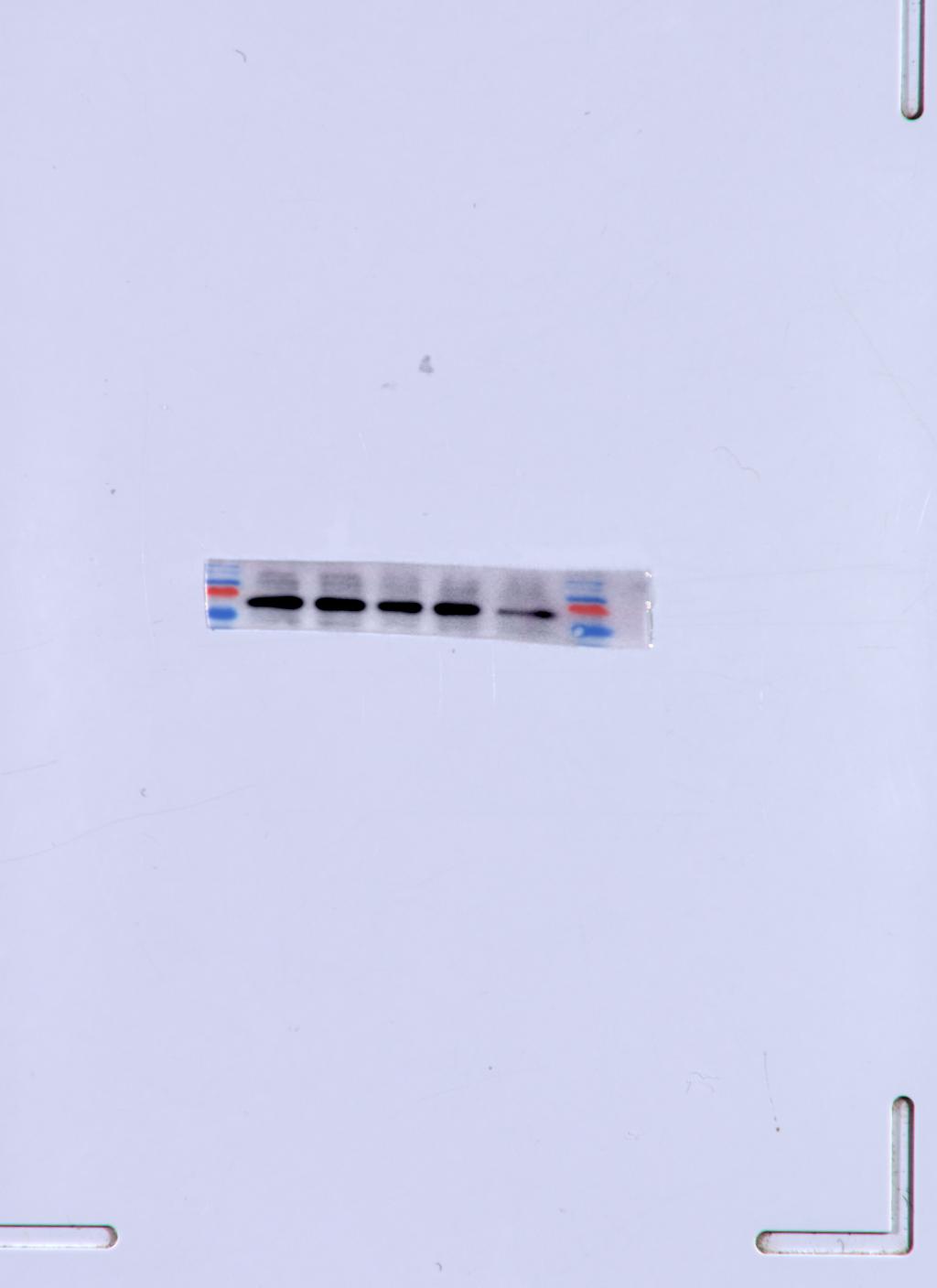

Supplement: Supplementary file 1 [file biology-11-01464-s001.zip › WB FIG/AC/h1-p53 2022.05.12_19.18.24_Ch+Marker.jpg]

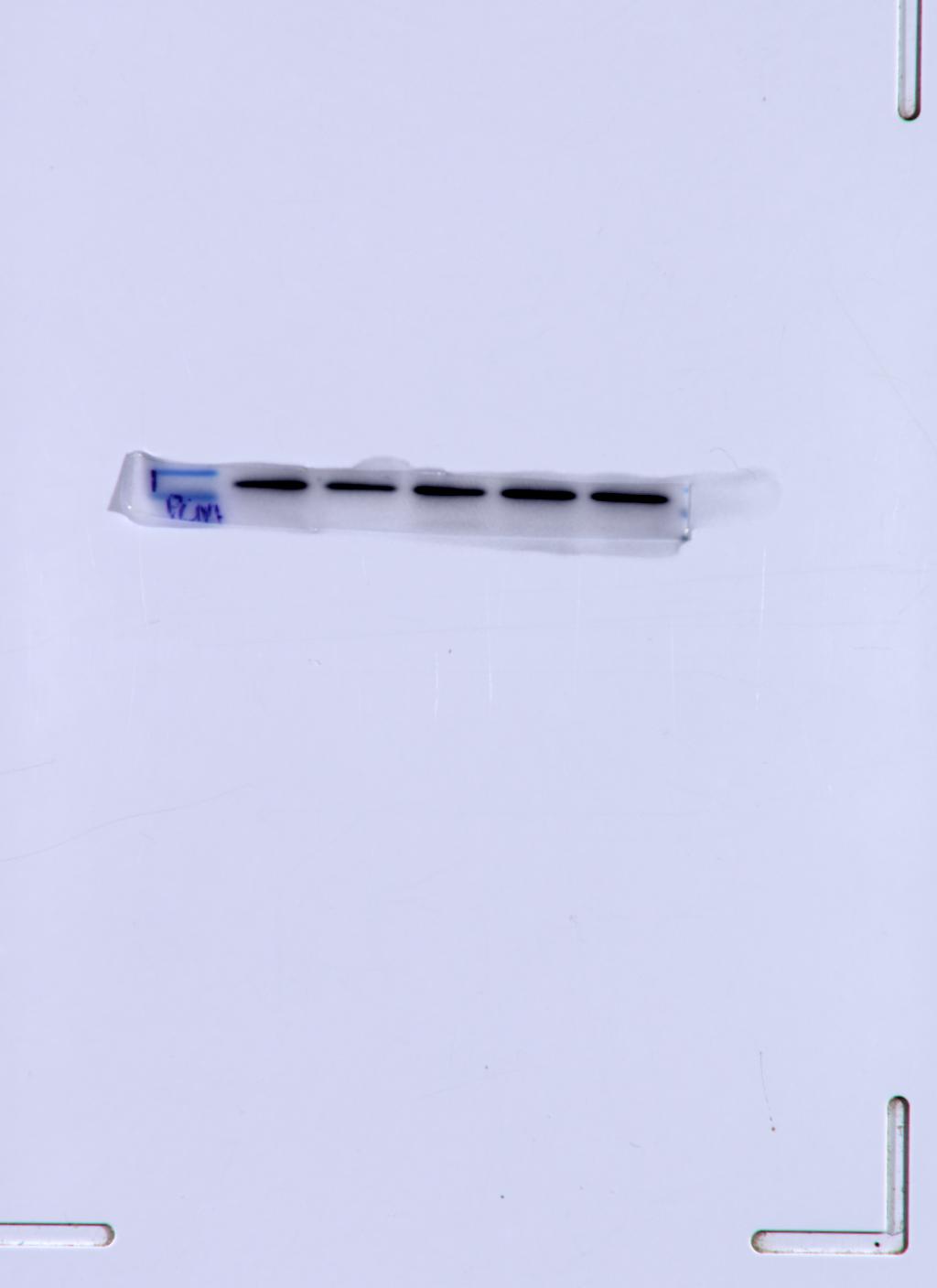

Supplement: Supplementary file 1 [file biology-11-01464-s001.zip › WB FIG/AC/h1-pcna 2022.05.10_12.09.12_Ch+Marker.jpg]

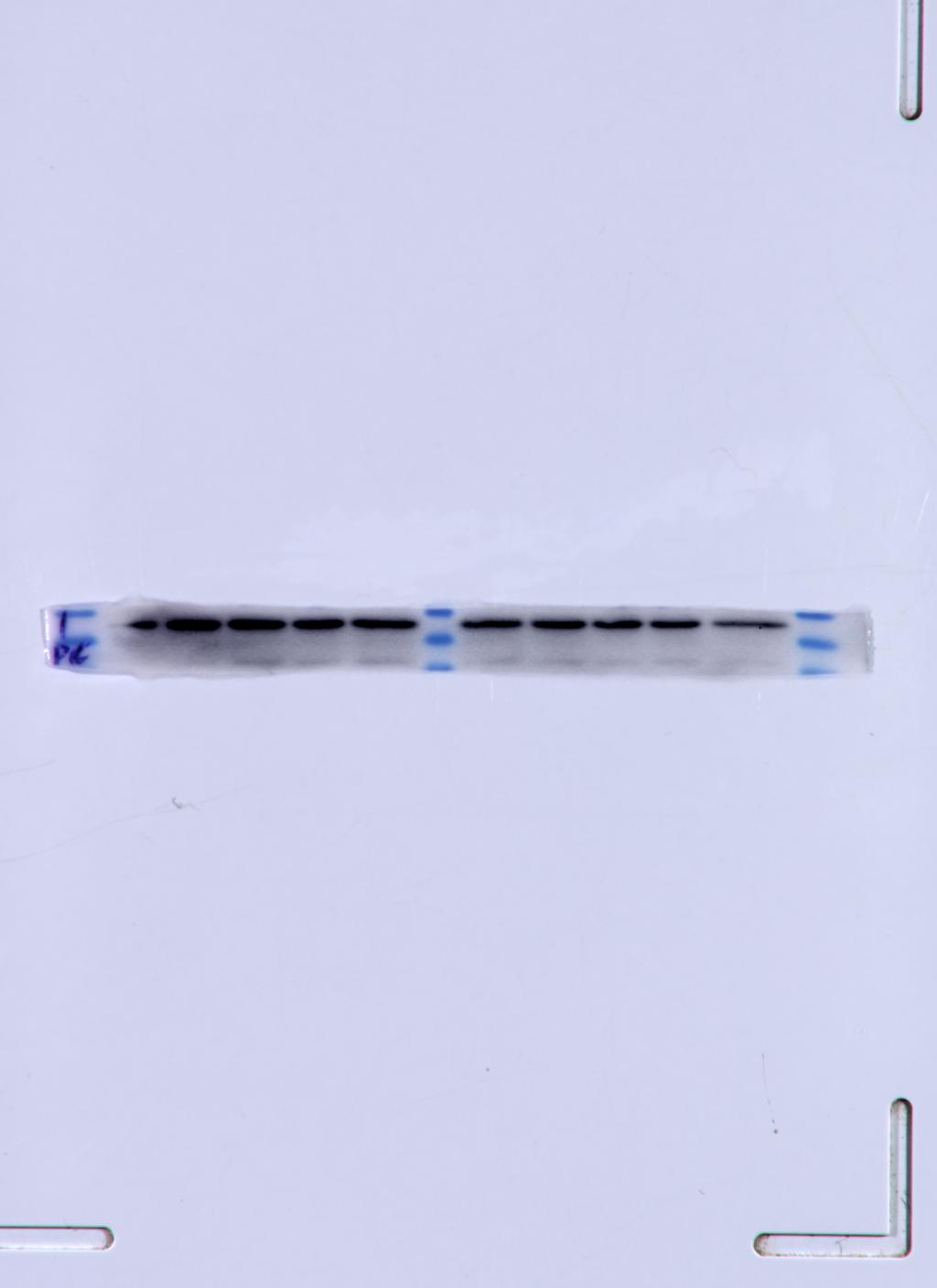

Supplement: Supplementary file 1 [file biology-11-01464-s001.zip › WB FIG/AC/h1-pcna 2022.05.12_19.01.52_Ch+Marker.jpg]

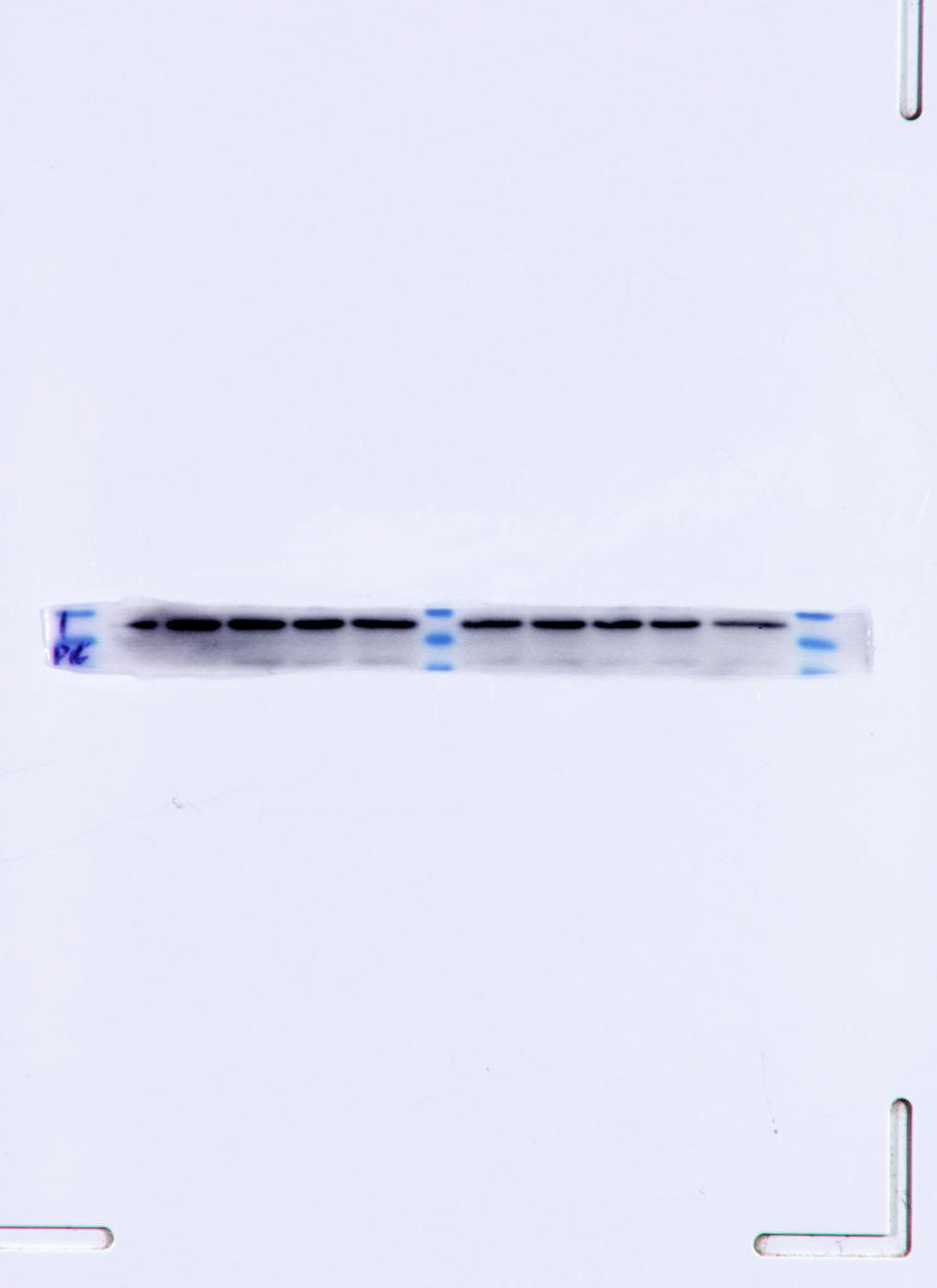

Supplement: Supplementary file 1 [file biology-11-01464-s001.zip › WB FIG/AC/h1-pcna 2022.05.12_PS.jpg]

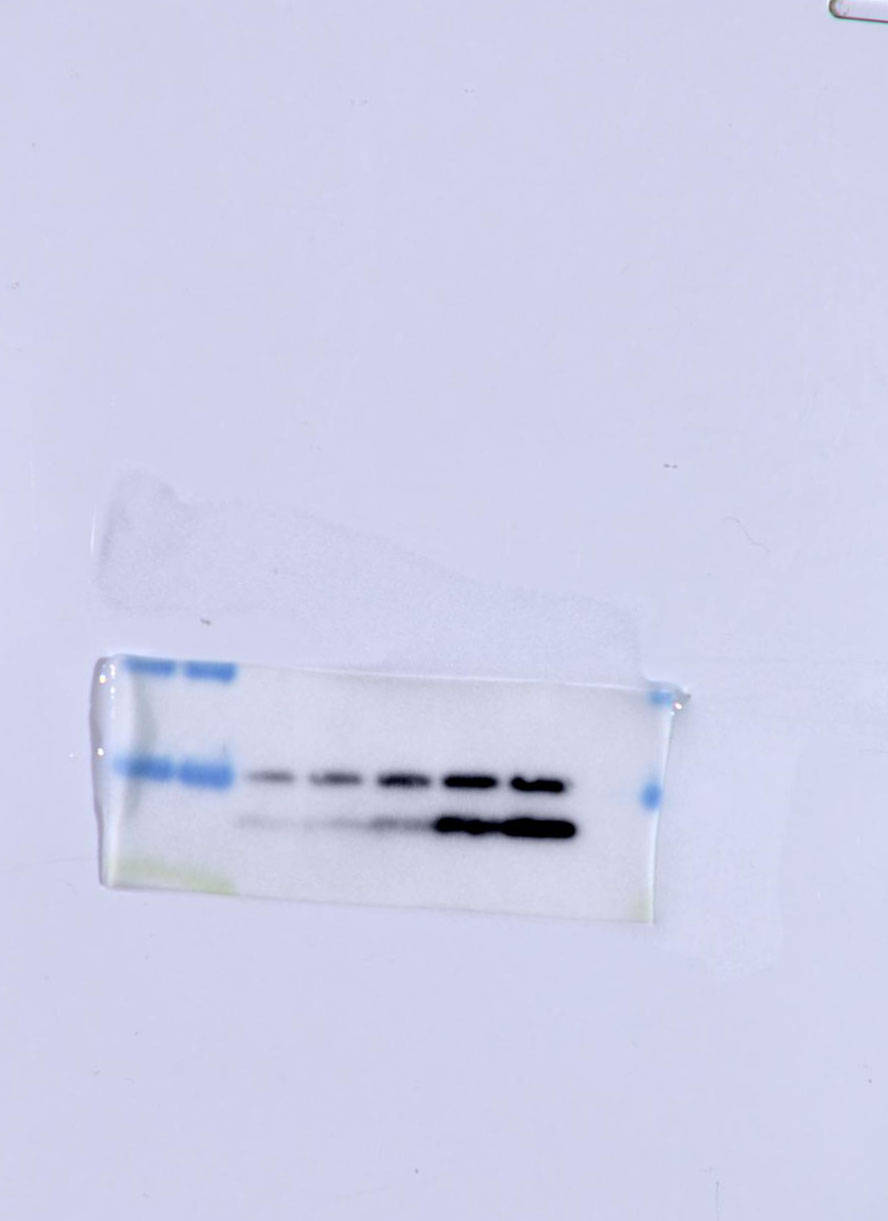

Supplement: Supplementary file 1 [file biology-11-01464-s001.zip › WB FIG/AC/H1yizhi-fanyi 2021.12.13_.jpg]

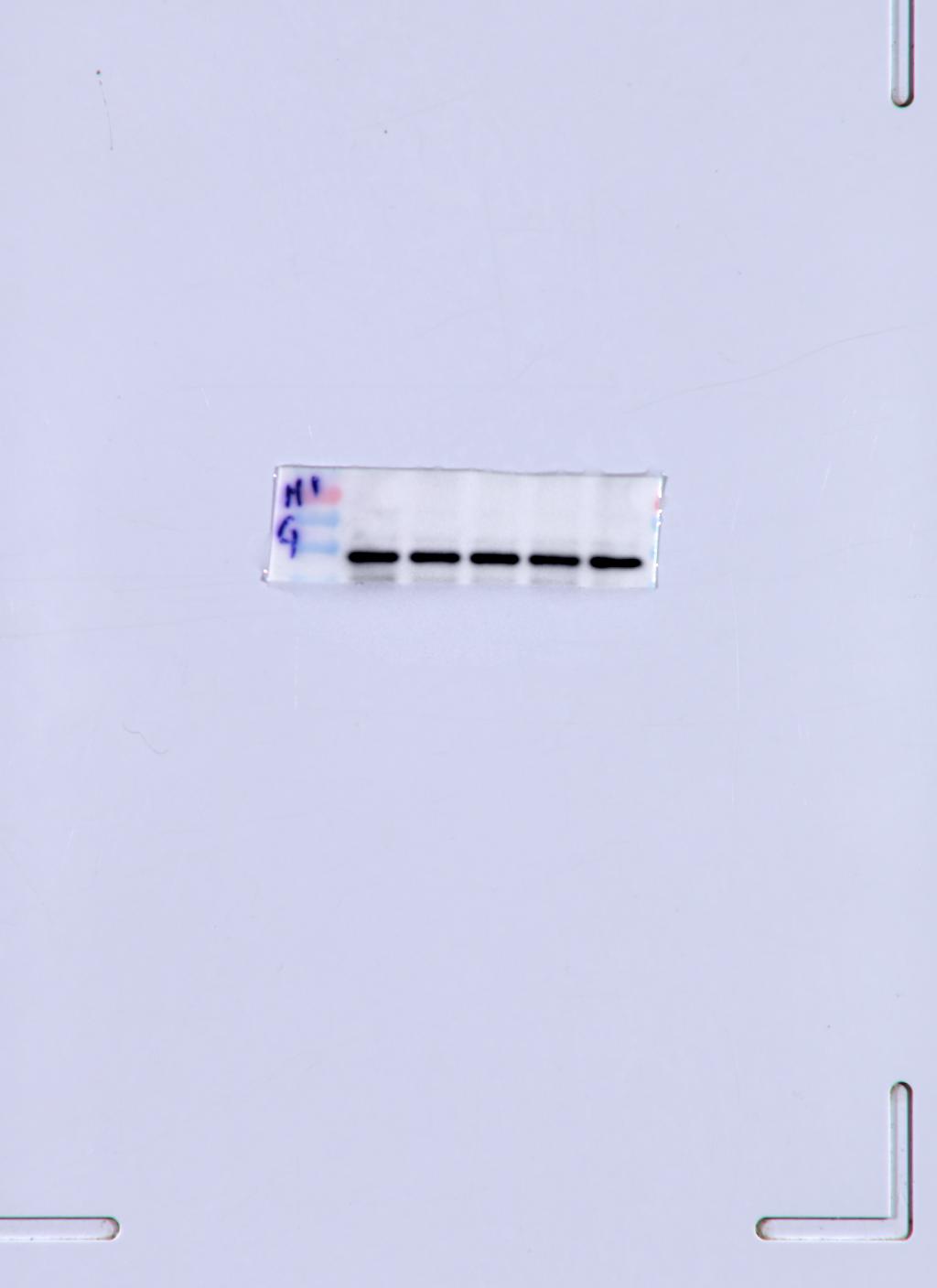

Supplement: Supplementary file 1 [file biology-11-01464-s001.zip › WB FIG/AC/hdac1-gap 2021.12.31_18.23.23_Ch+Marker.jpg]

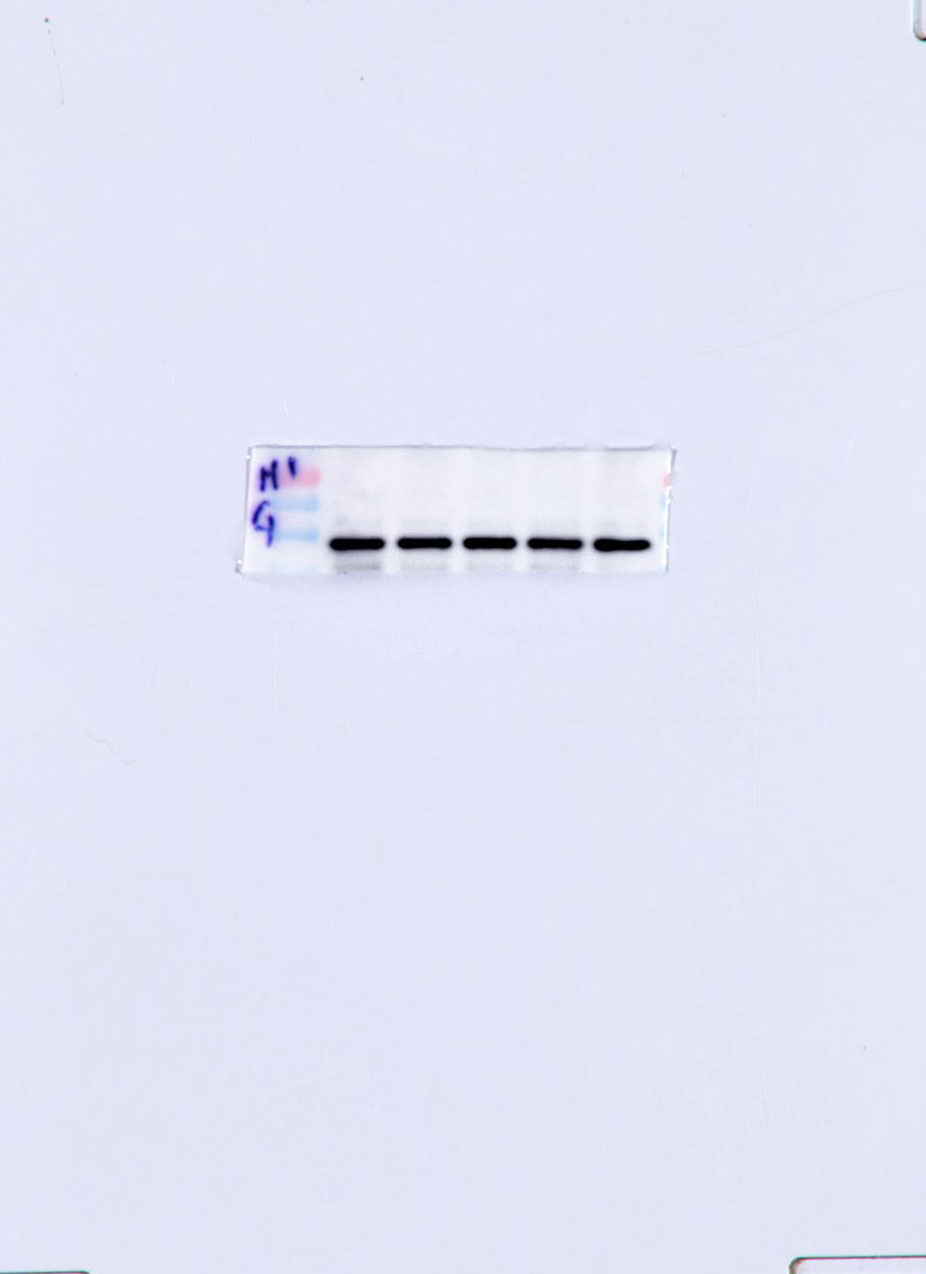

Supplement: Supplementary file 1 [file biology-11-01464-s001.zip › WB FIG/AC/hdac1-gap 2021.12.31_PS.jpg]

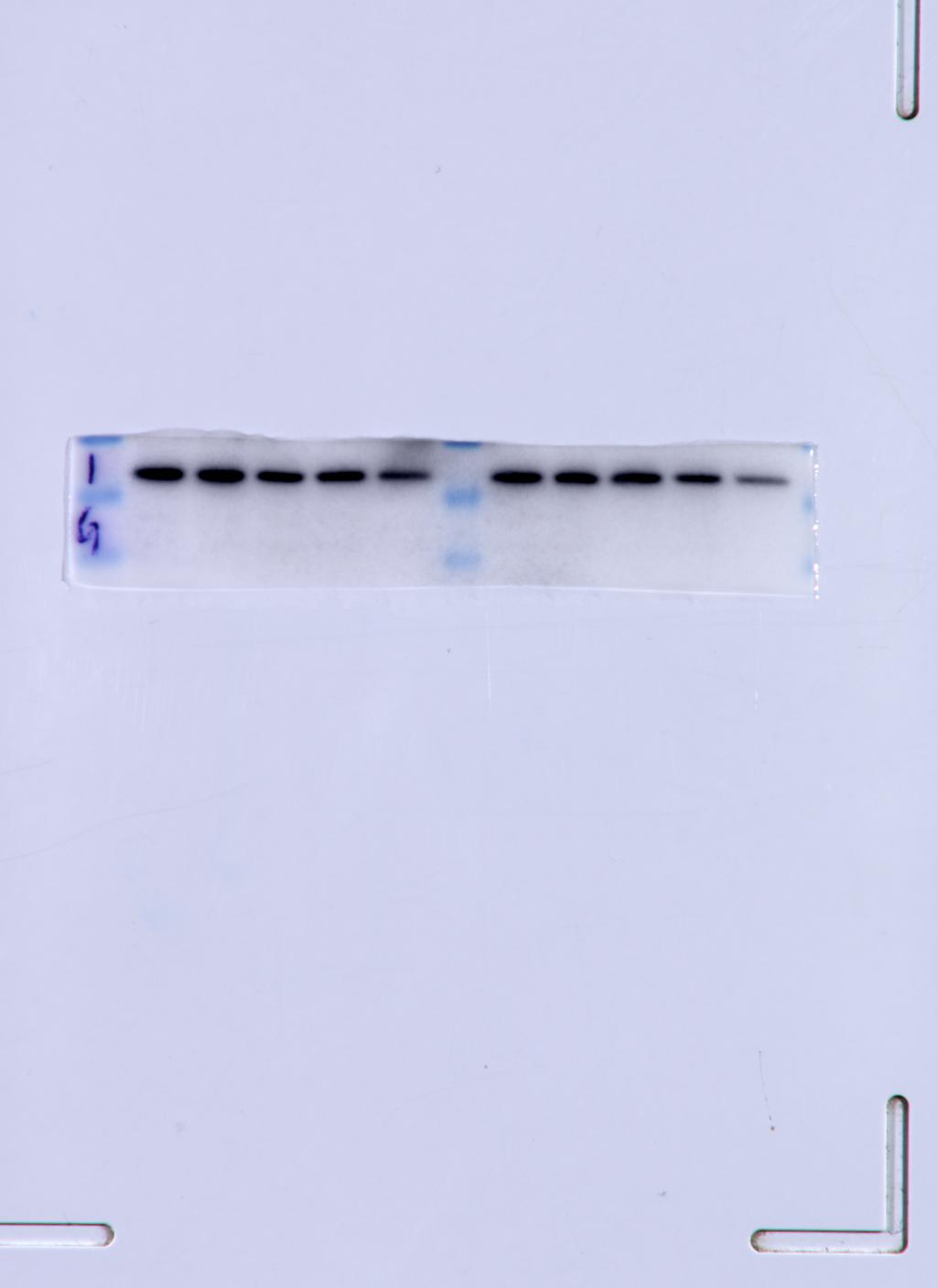

Supplement: Supplementary file 1 [file biology-11-01464-s001.zip › WB FIG/AC/hdac1-nongdu-gap 2022.04.26_13.41.38_Ch+Marker.jpg]

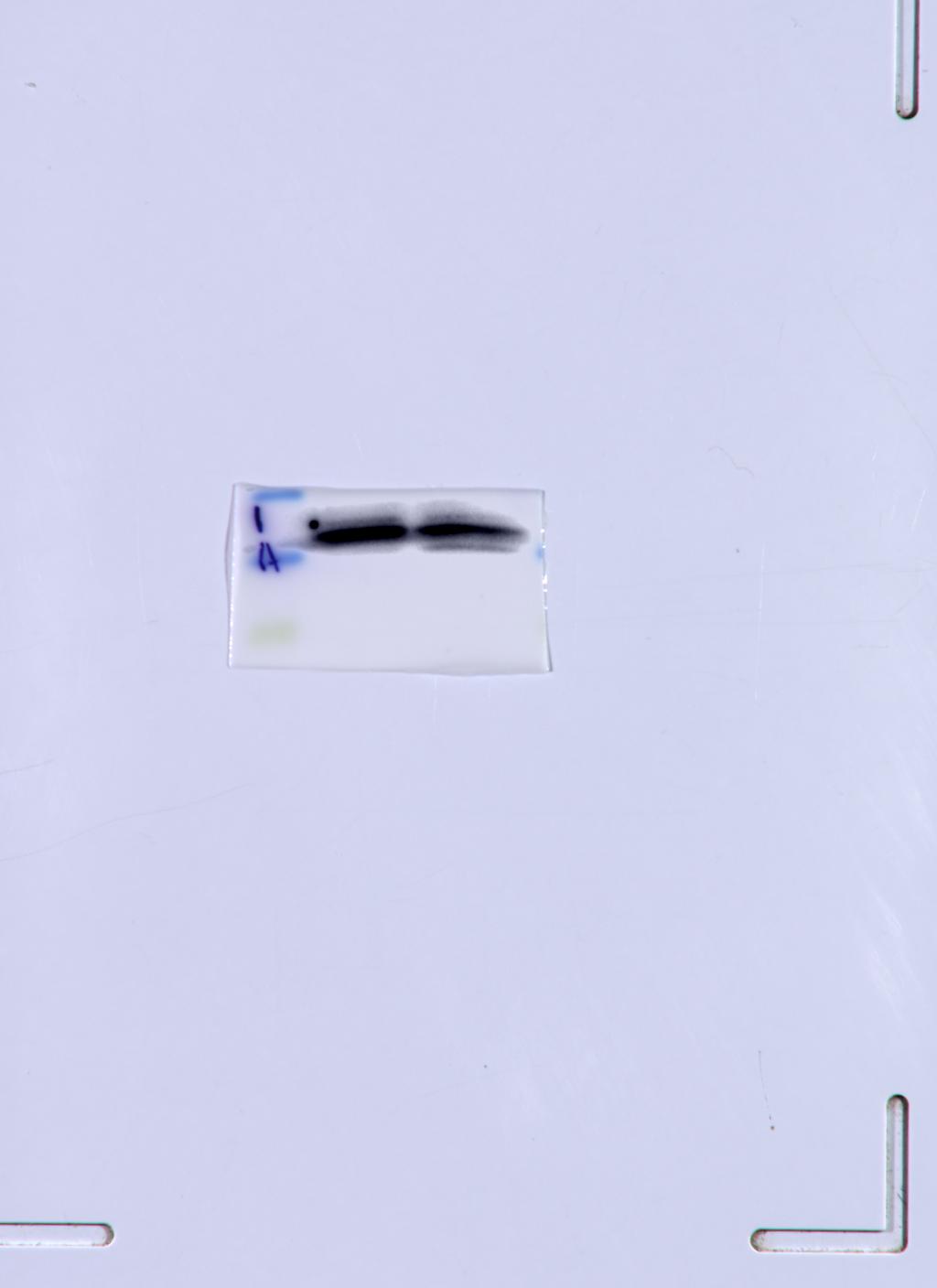

Supplement: Supplementary file 1 [file biology-11-01464-s001.zip › WB FIG/AREG/h1-areg 2022.05.12_20.03.48_Ch+Marker.jpg]

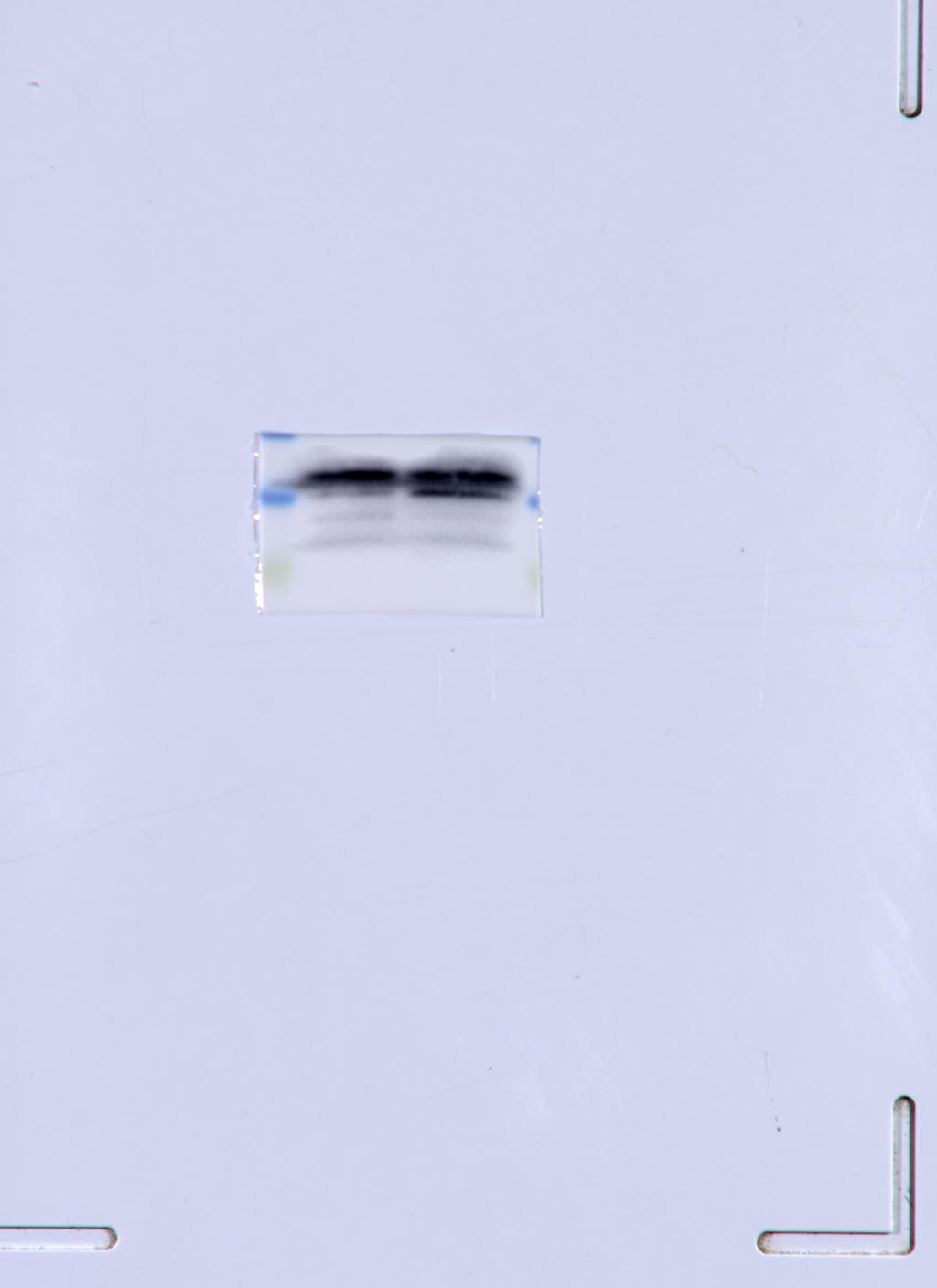

Supplement: Supplementary file 1 [file biology-11-01464-s001.zip › WB FIG/AREG/h1-areg 2022.05.12_20.06.19_Ch+Marker.jpg]

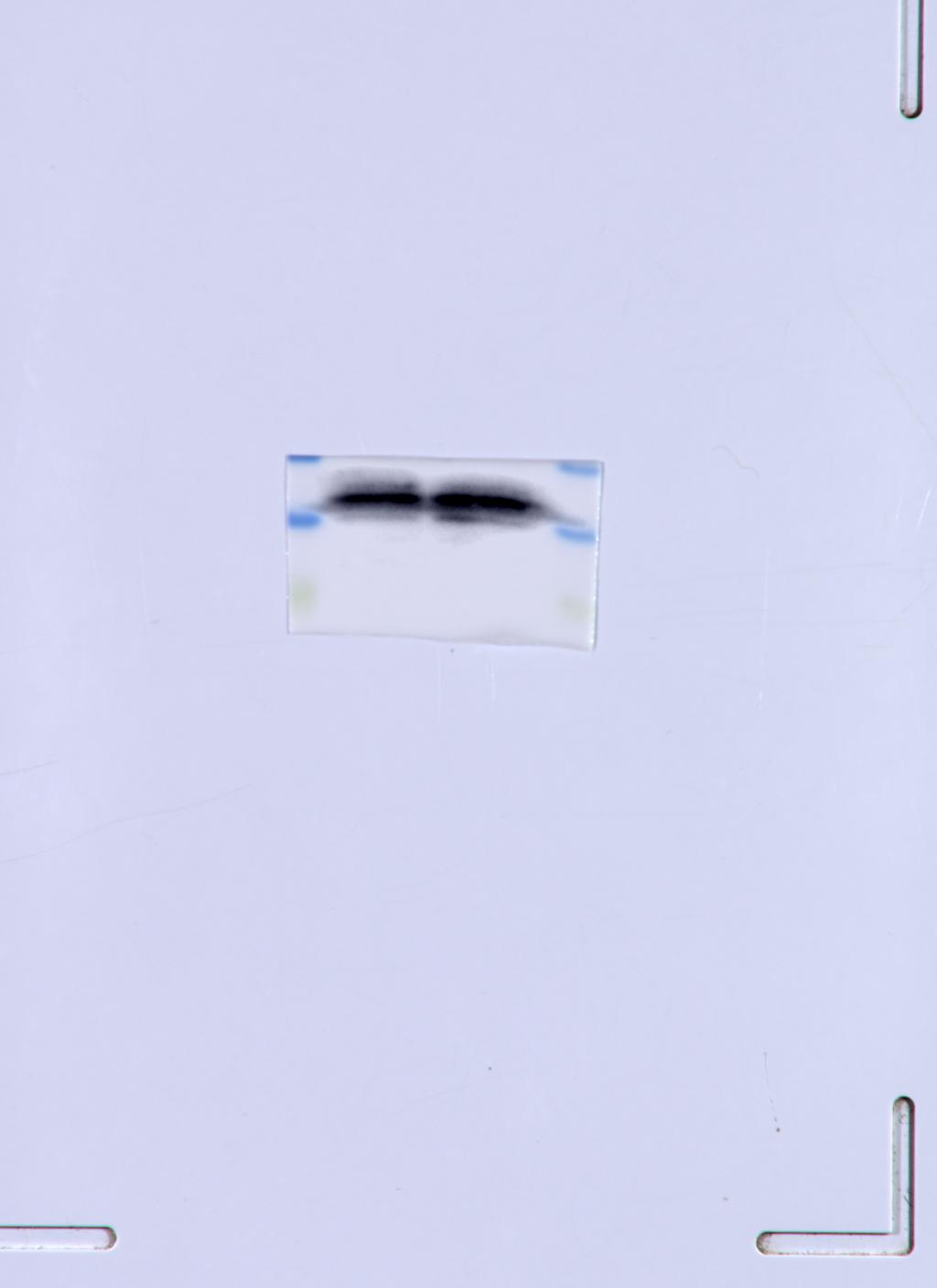

Supplement: Supplementary file 1 [file biology-11-01464-s001.zip › WB FIG/AREG/h1-areg 2022.05.12_20.10.14_Ch+Marker.jpg]

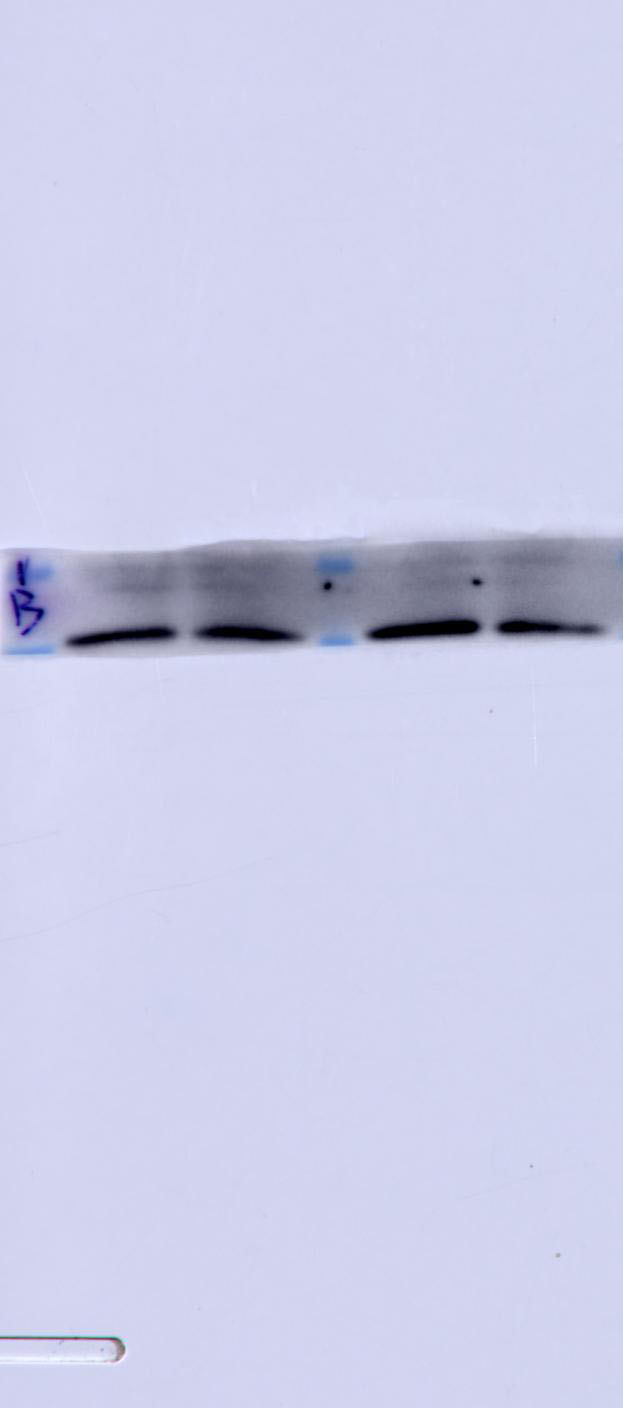

Supplement: Supplementary file 1 [file biology-11-01464-s001.zip › WB FIG/BTC/h1-30ug-btc 2022.04.28_.jpg]

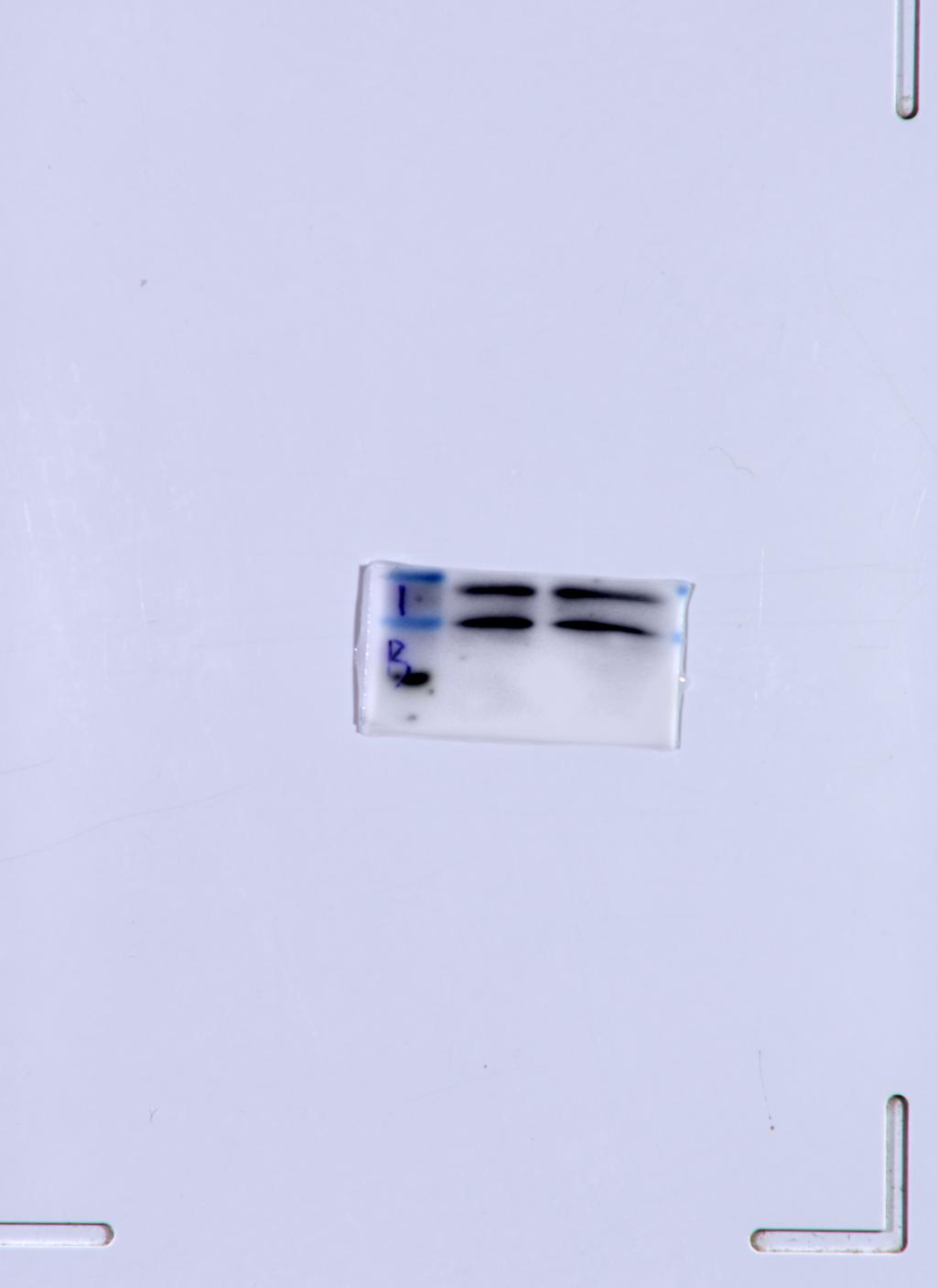

Supplement: Supplementary file 1 [file biology-11-01464-s001.zip › WB FIG/BTC/h1-btc 2022.05.14_18.22.53_Ch+Marker.jpg]

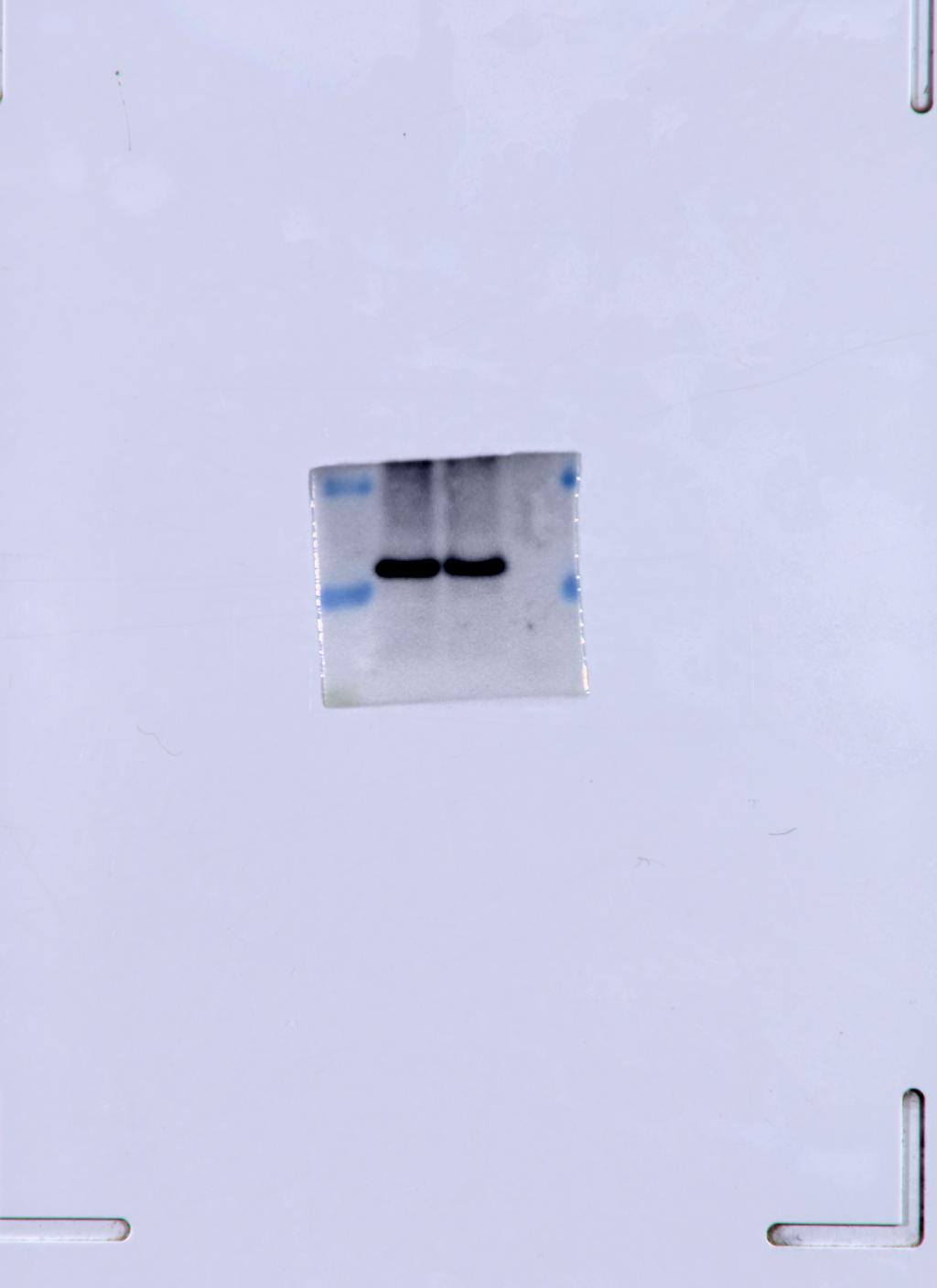

Supplement: Supplementary file 1 [file biology-11-01464-s001.zip › WB FIG/BTC/hdac1.yzj-btc 2021.12.23_13.58.47_Ch+Marker.jpg]

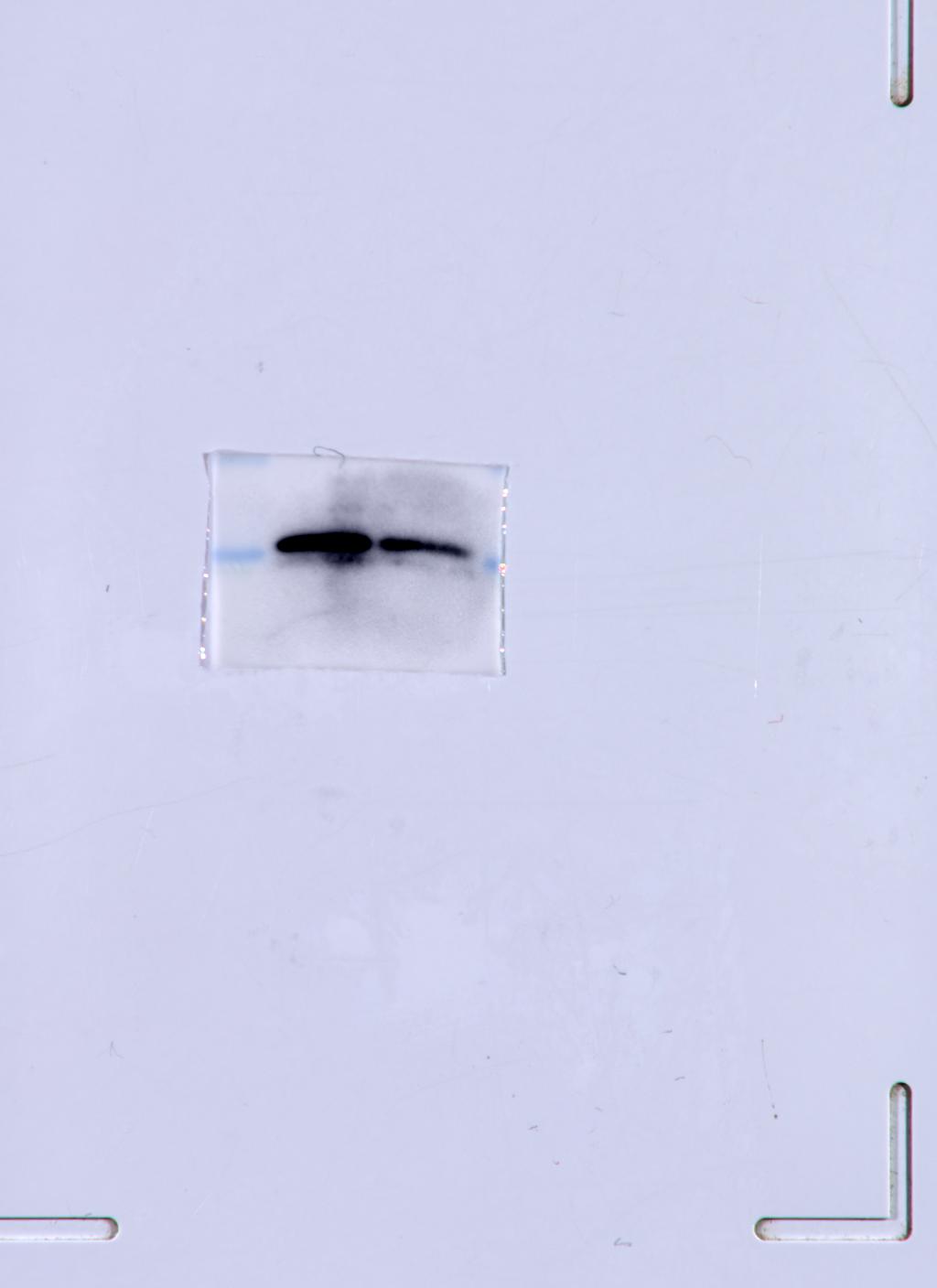

Supplement: Supplementary file 1 [file biology-11-01464-s001.zip › WB FIG/EREG/h1yizhiji-ereg 2022.03.22_20.51.43_Ch+Marker.jpg]

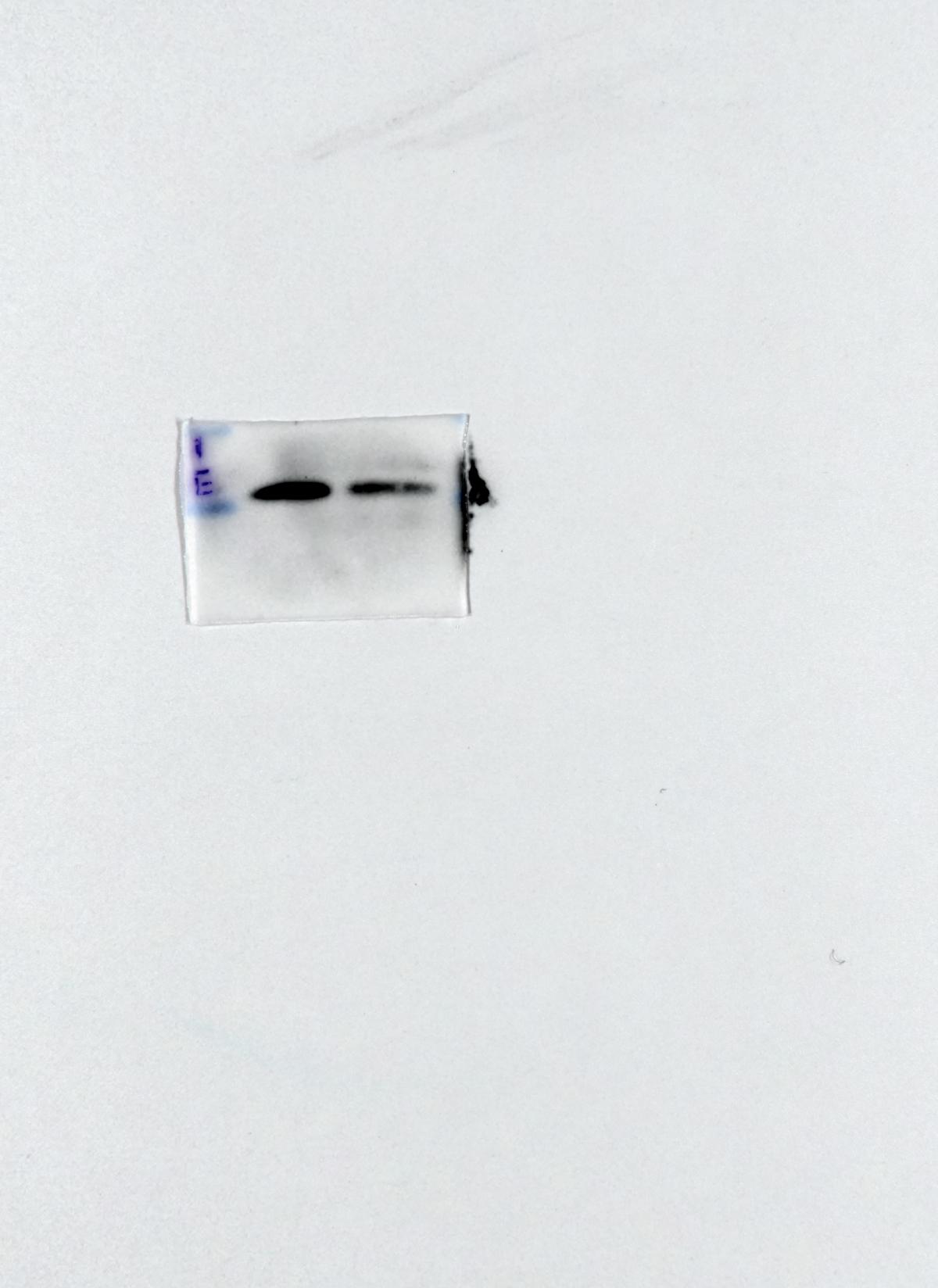

Supplement: Supplementary file 1 [file biology-11-01464-s001.zip › WB FIG/EREG/h1yzj-ereg.1 20220324_142012_Ch_Chemi+Marker.jpg]

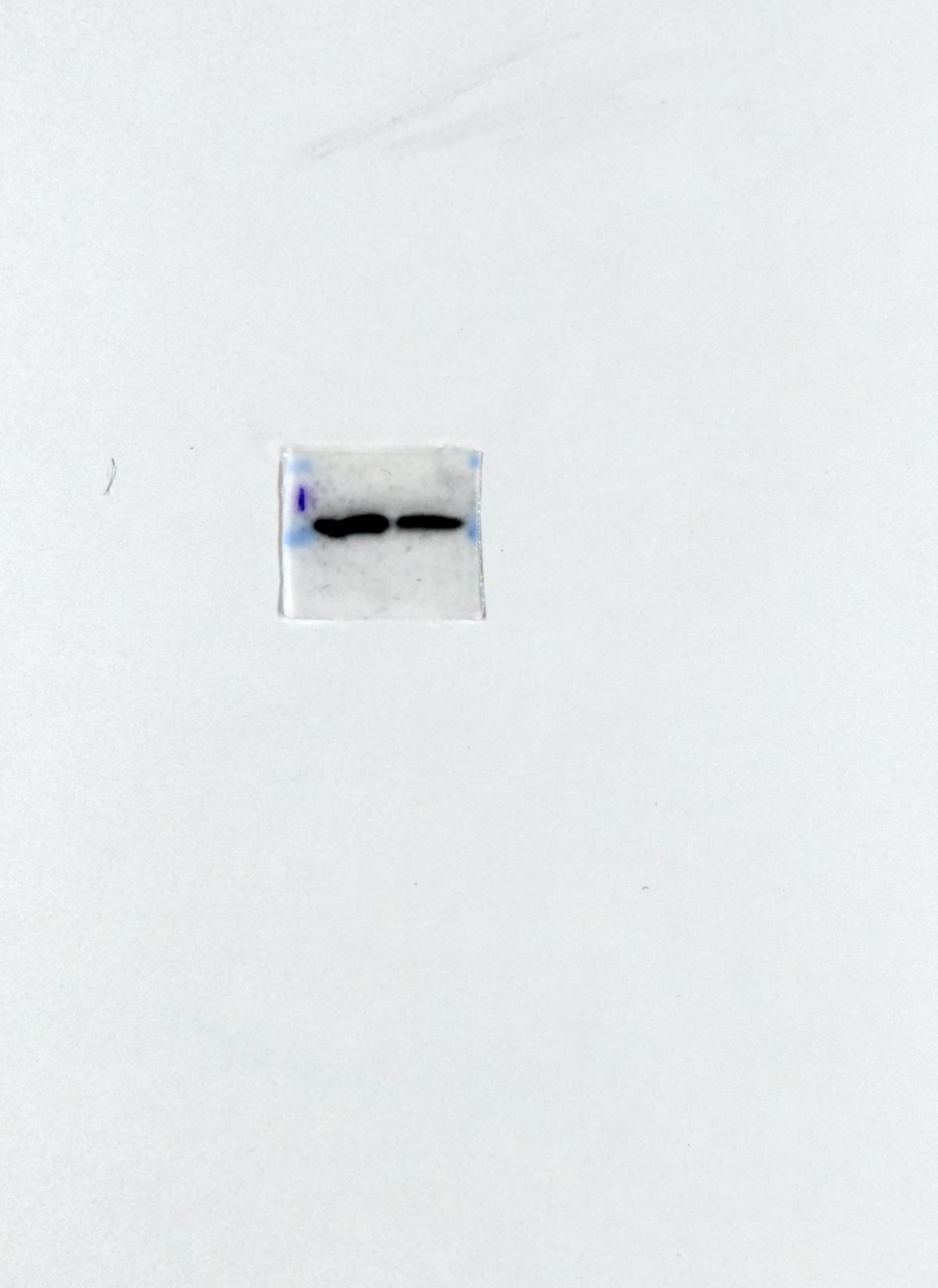

Supplement: Supplementary file 1 [file biology-11-01464-s001.zip › WB FIG/EREG/hdac1yizhiji-ereg.1 20220327_171314_Ch_Chemi+Marker.jpg]

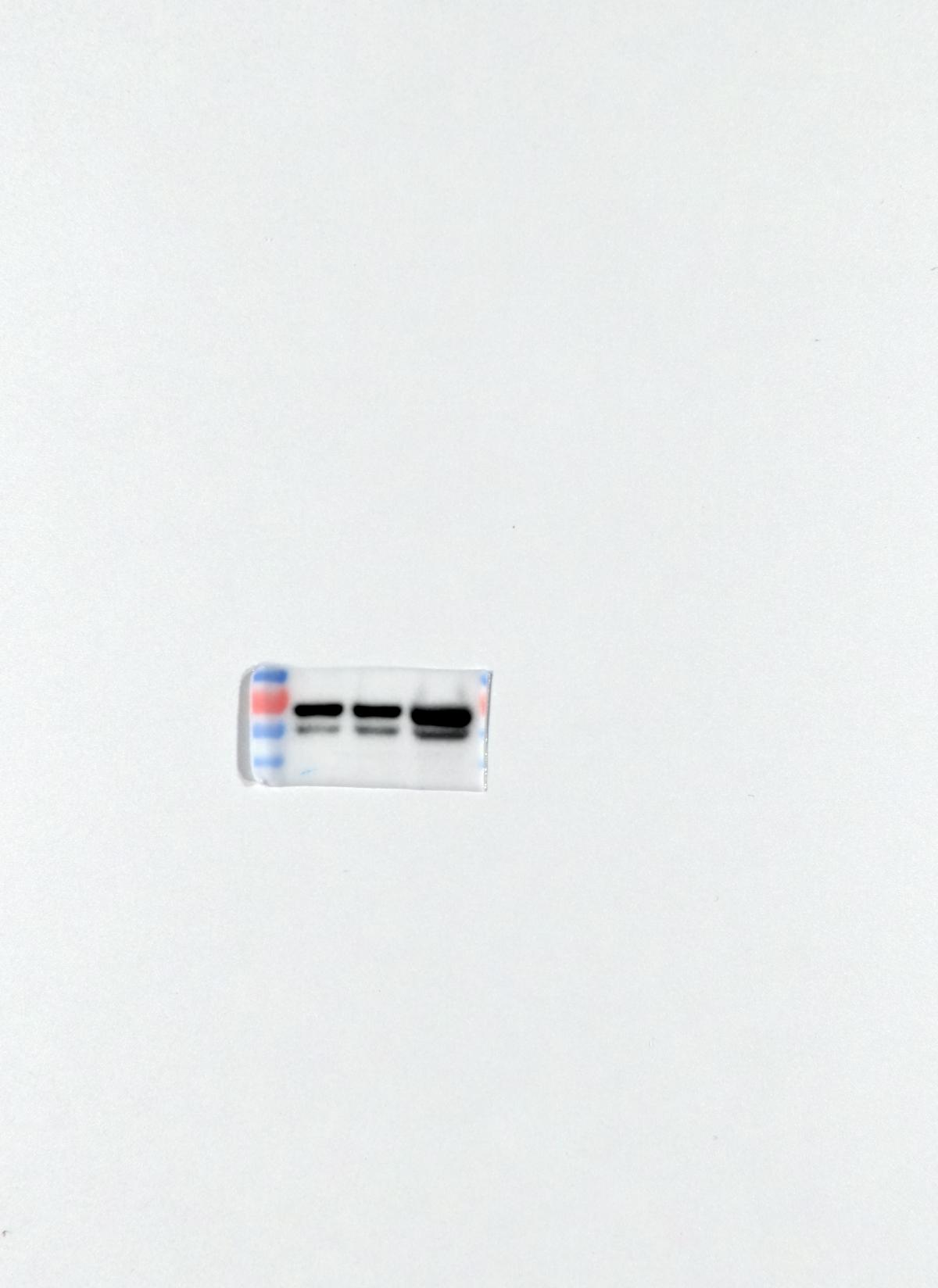

Supplement: Supplementary file 1 [file biology-11-01464-s001.zip › WB FIG/follice/hdac1,luanpao 20211229_134244_Ch_Chemi+Marker.jpg]

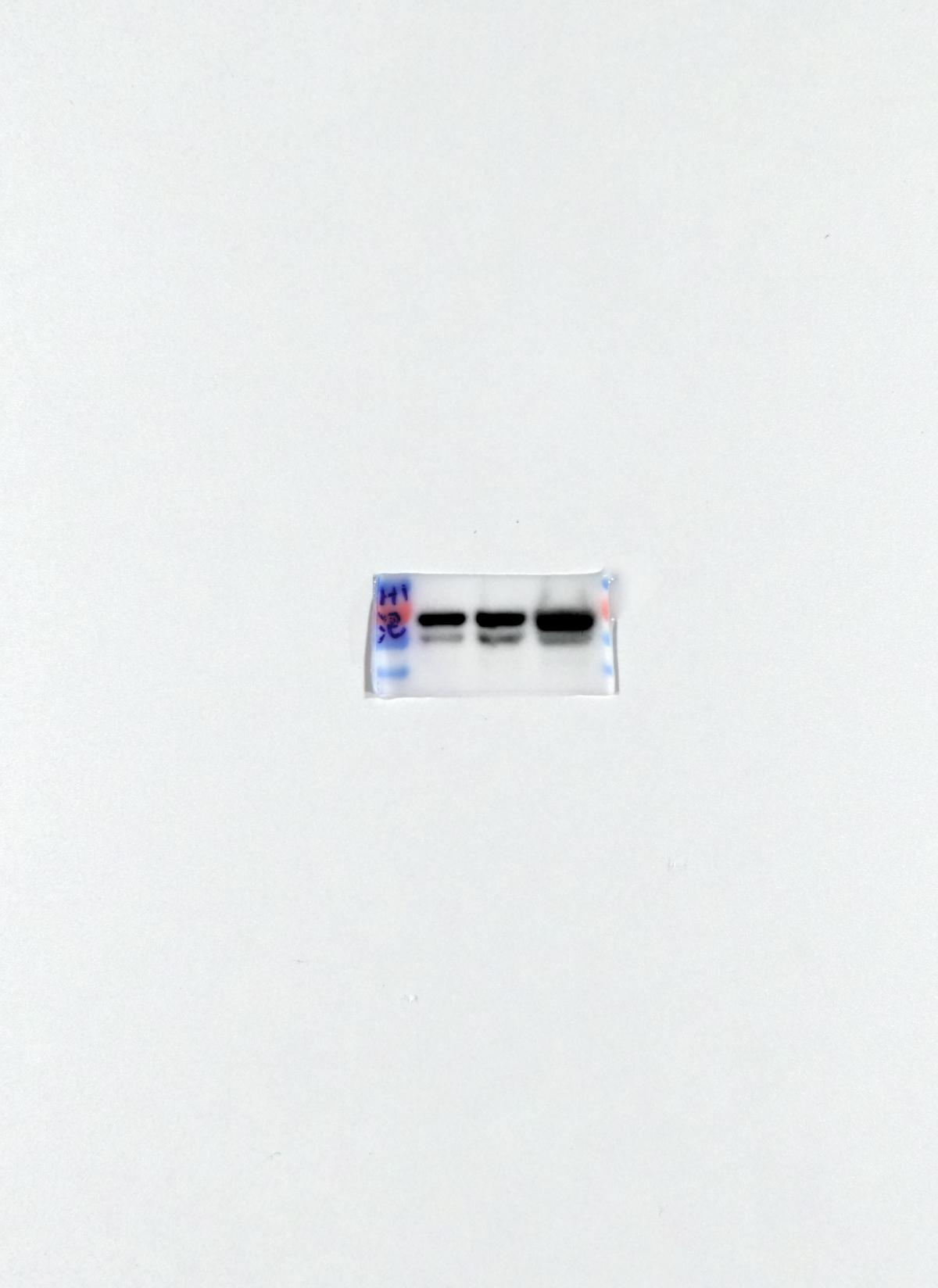

Supplement: Supplementary file 1 [file biology-11-01464-s001.zip › WB FIG/follice/hdac1,luanpao 20211229_134438_Ch_Chemi+Marker.jpg]

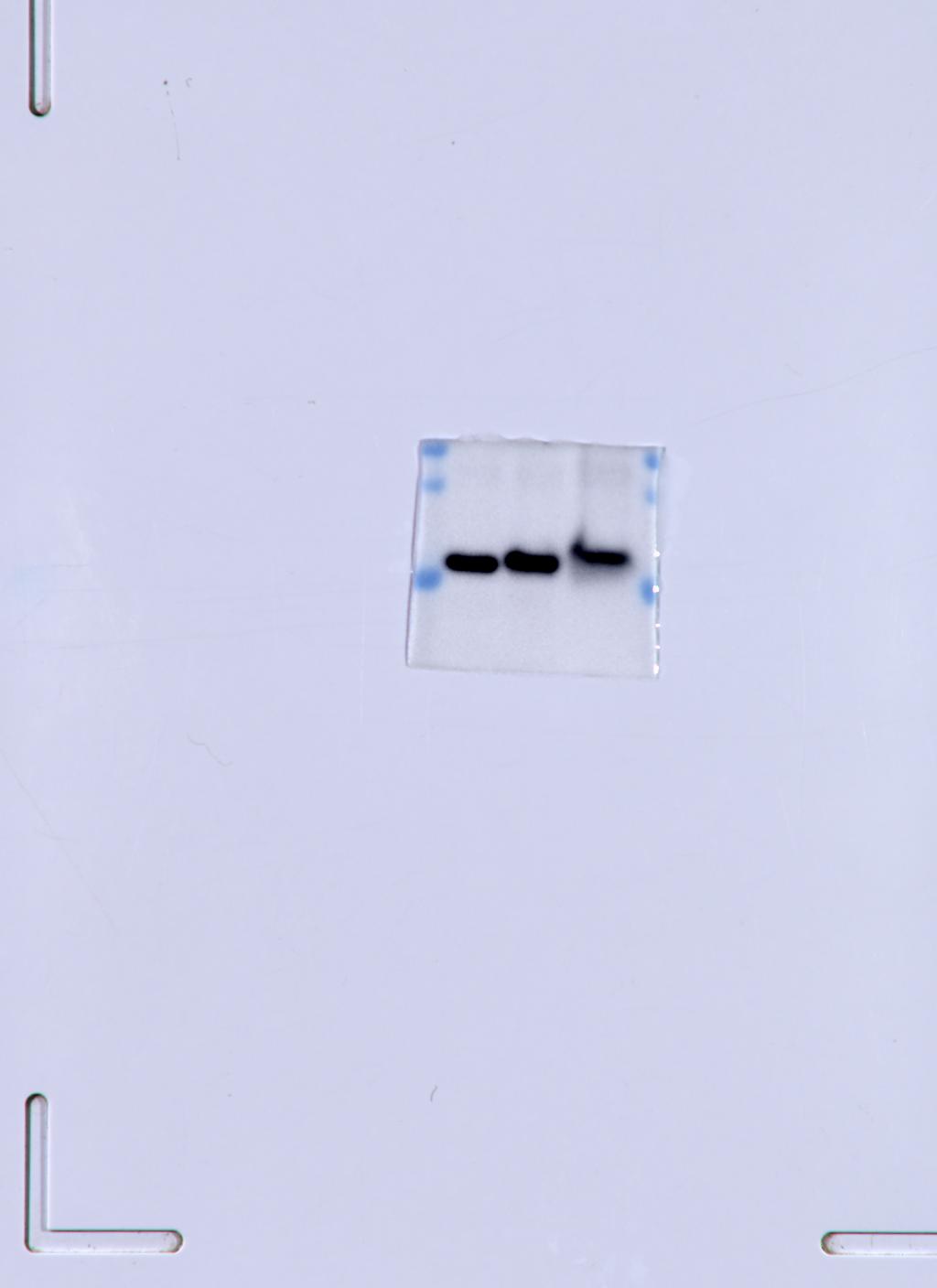

Supplement: Supplementary file 1 [file biology-11-01464-s001.zip › WB FIG/follice/luanpao-h3 2021.12.23_11.09.08_Ch+Marker.jpg]

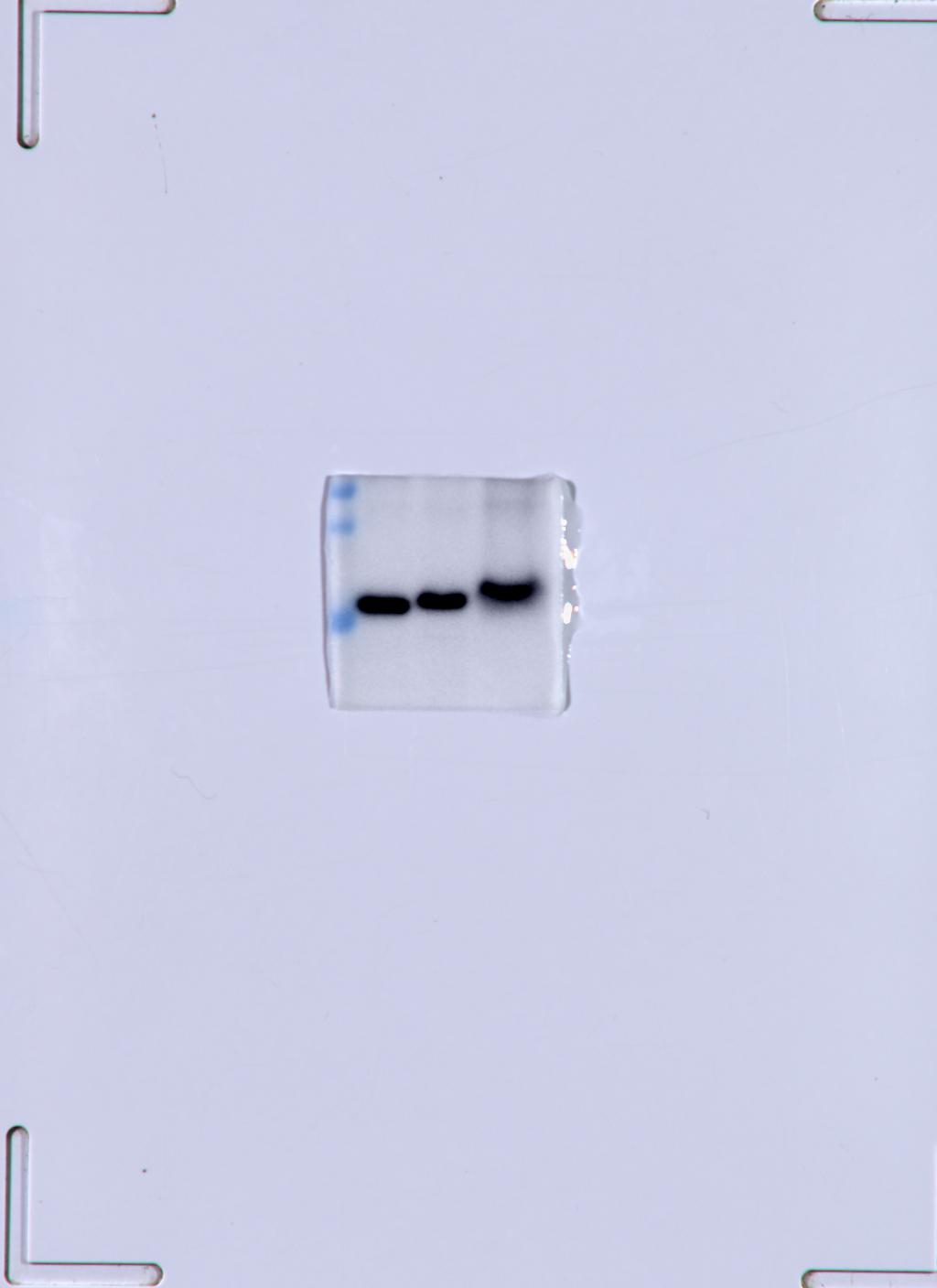

Supplement: Supplementary file 1 [file biology-11-01464-s001.zip › WB FIG/follice/luanpao-h3.1 2021.12.23_11.11.33_Ch+Marker.jpg]

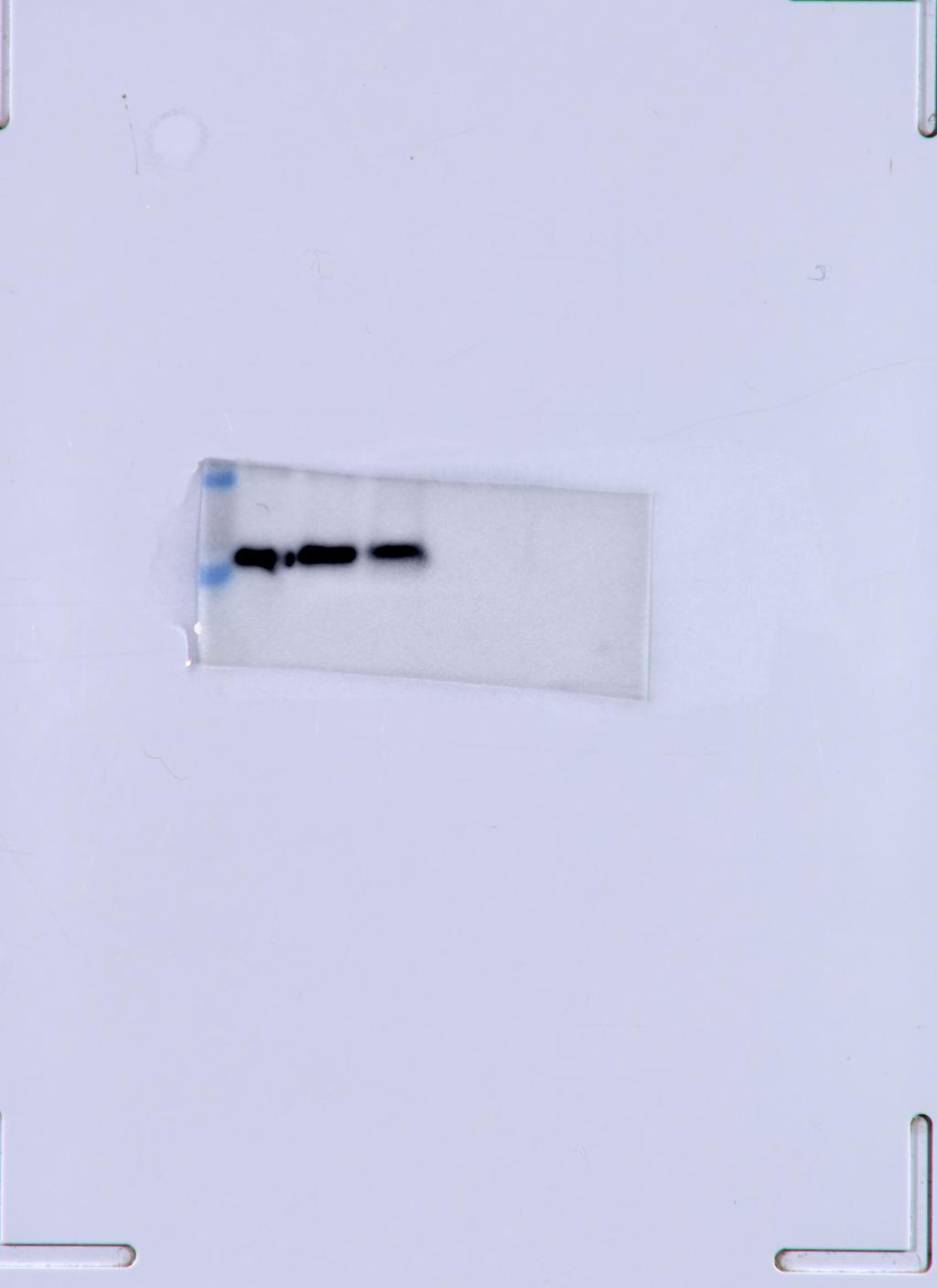

Supplement: Supplementary file 1 [file biology-11-01464-s001.zip › WB FIG/follice/luanpao-h3nc 2021.12.21_17.20.11_Ch+Marker.jpg]

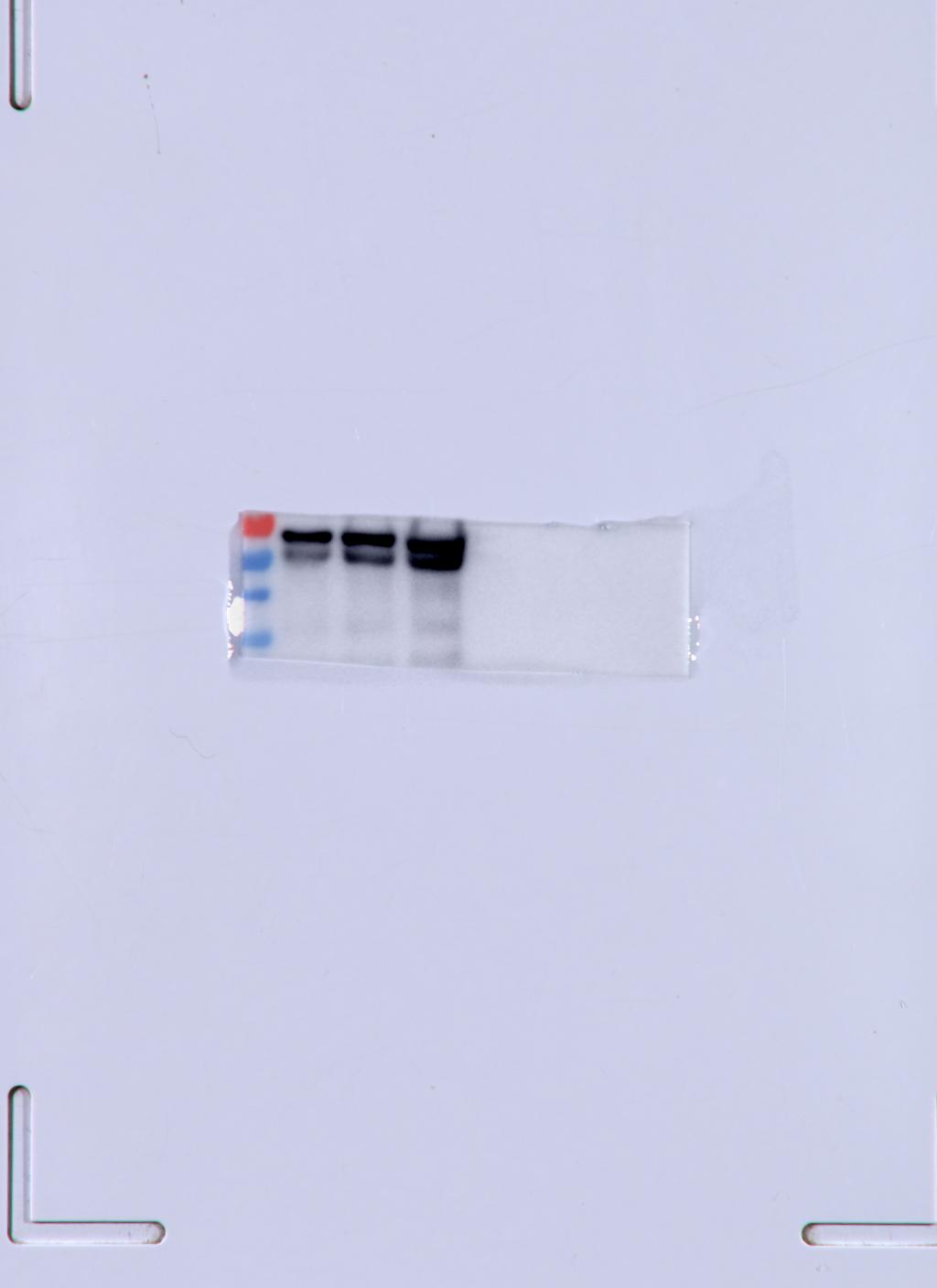

Supplement: Supplementary file 1 [file biology-11-01464-s001.zip › WB FIG/follice/luanpao-hdac1 2021.12.21_17.36.57_Ch+Marker.jpg]

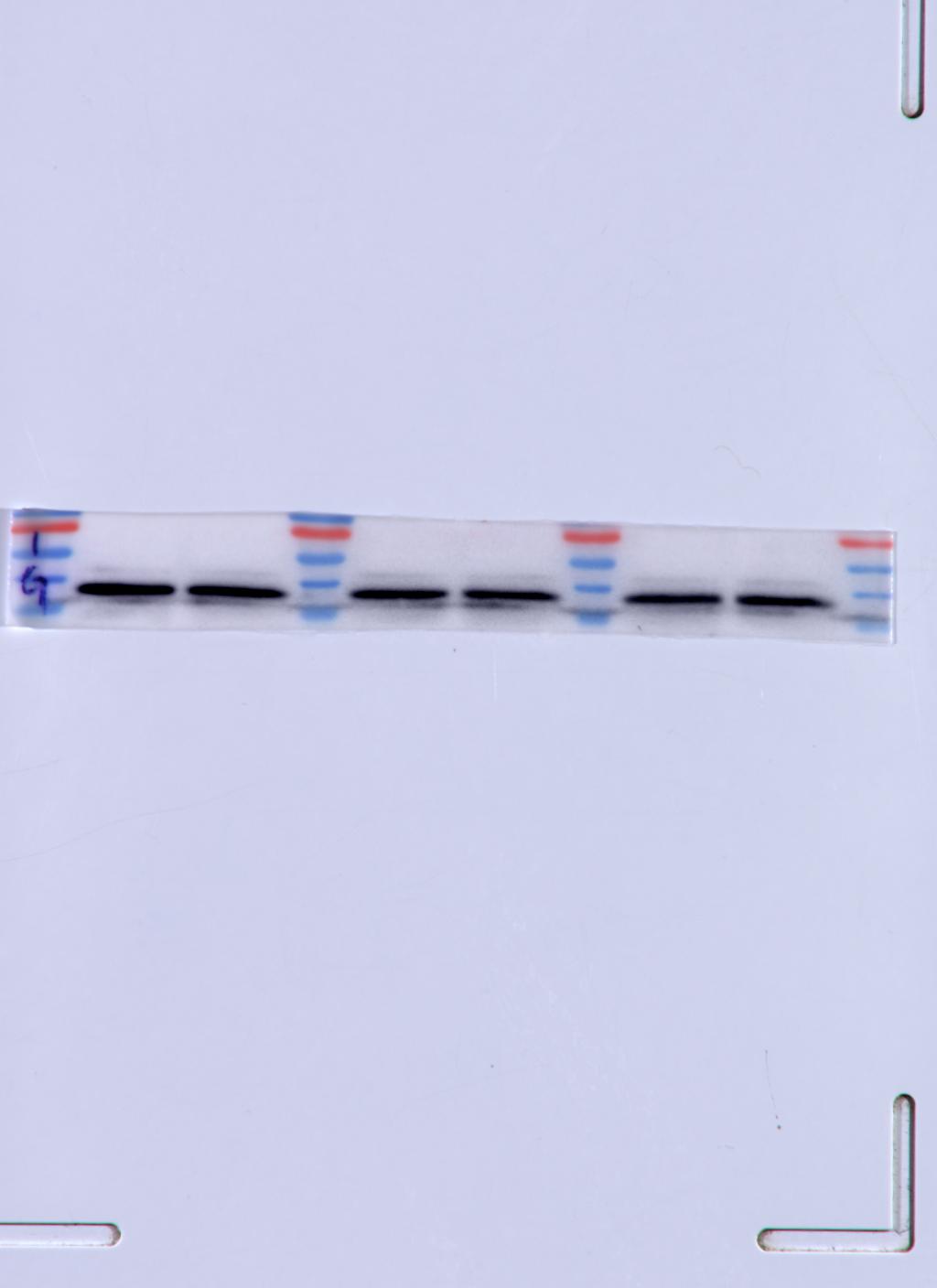

Supplement: Supplementary file 1 [file biology-11-01464-s001.zip › WB FIG/GAP/h1-30ug-gap 2022.04.28_19.43.19_Ch+Marker.jpg]

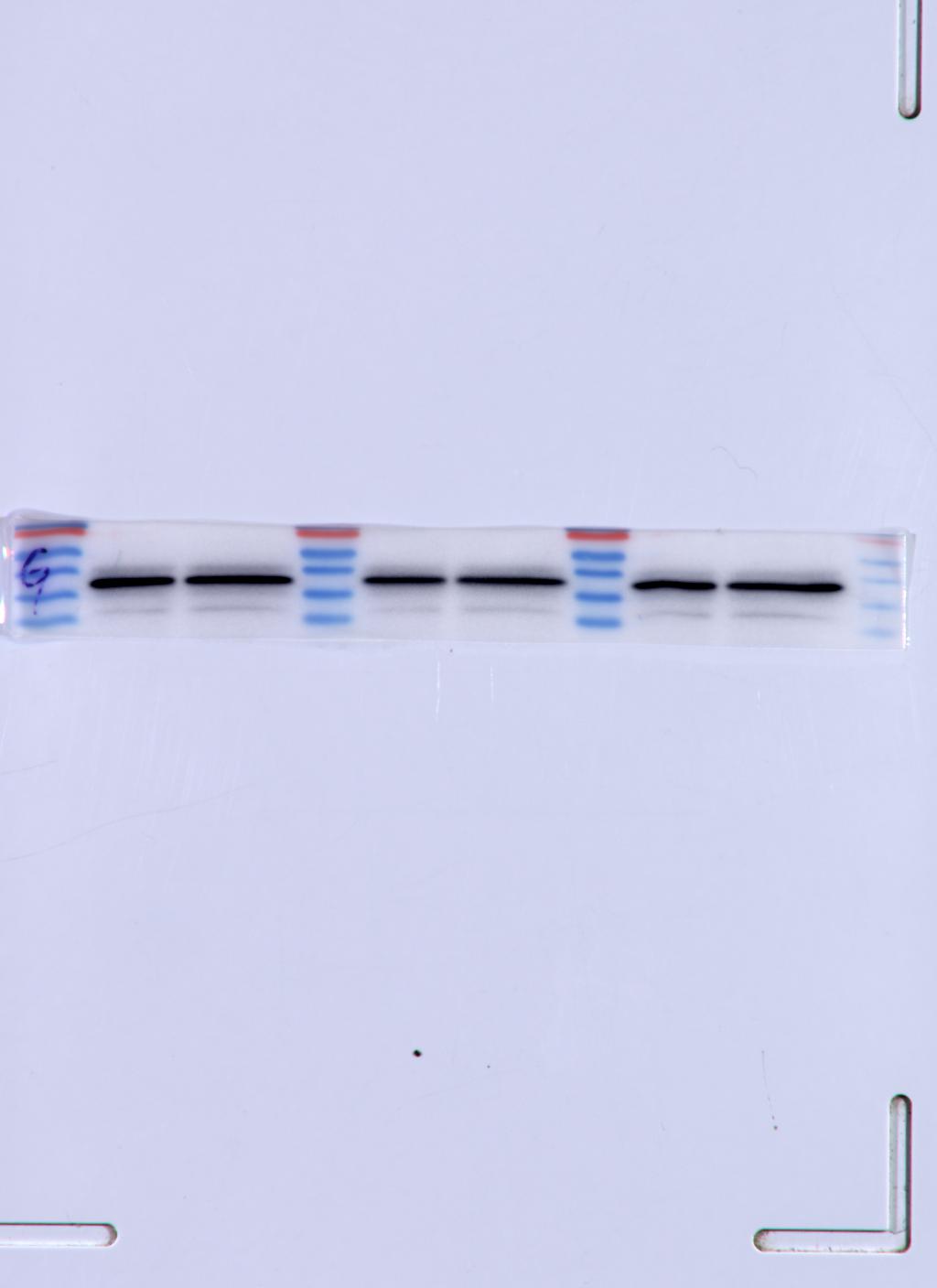

Supplement: Supplementary file 1 [file biology-11-01464-s001.zip › WB FIG/GAP/h1-gap 2022.05.05_17.11.59_Ch+Marker.jpg]

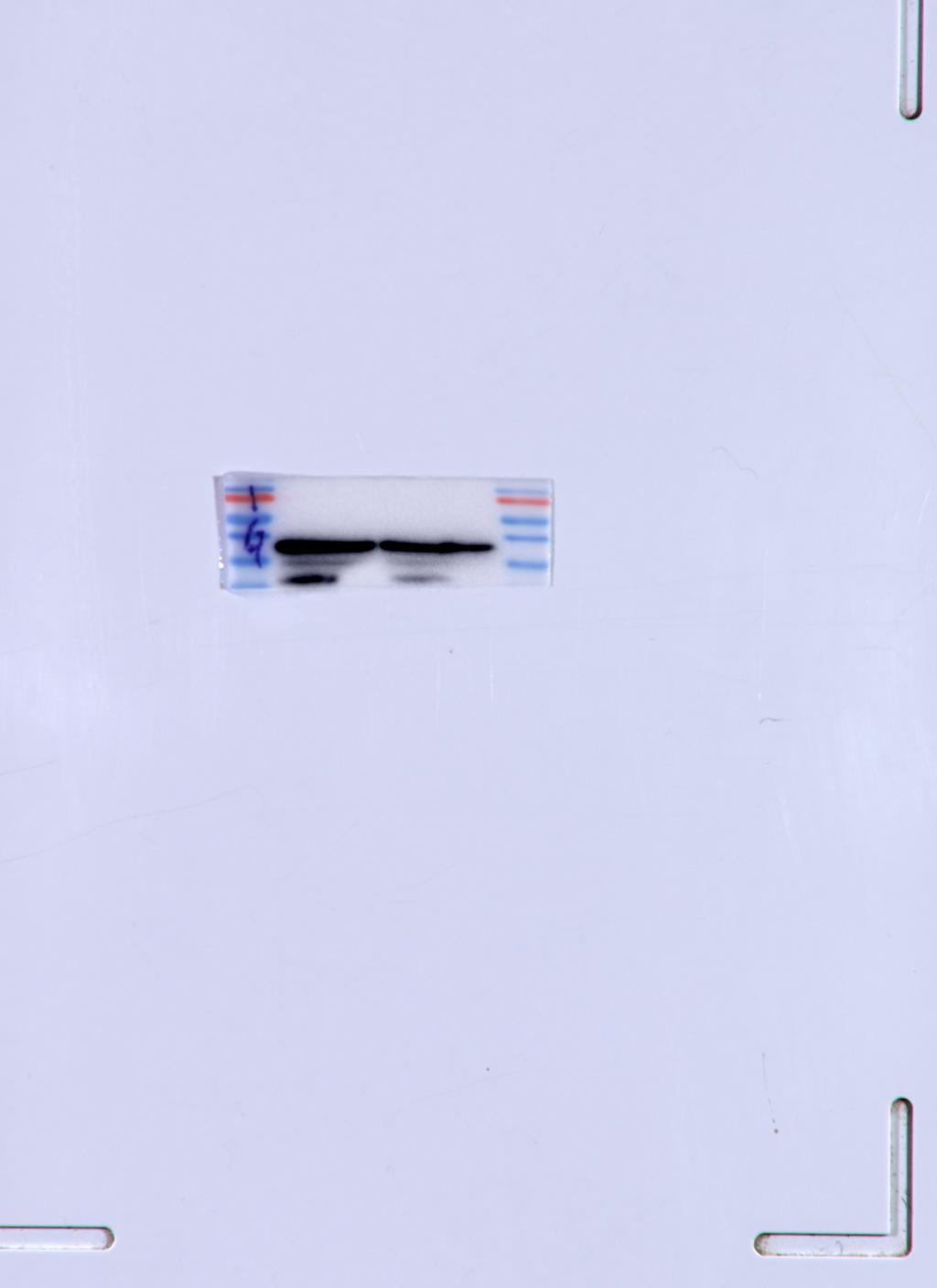

Supplement: Supplementary file 1 [file biology-11-01464-s001.zip › WB FIG/GAP/h1-gap 2022.05.12_19.26.22_Ch+Marker.jpg]

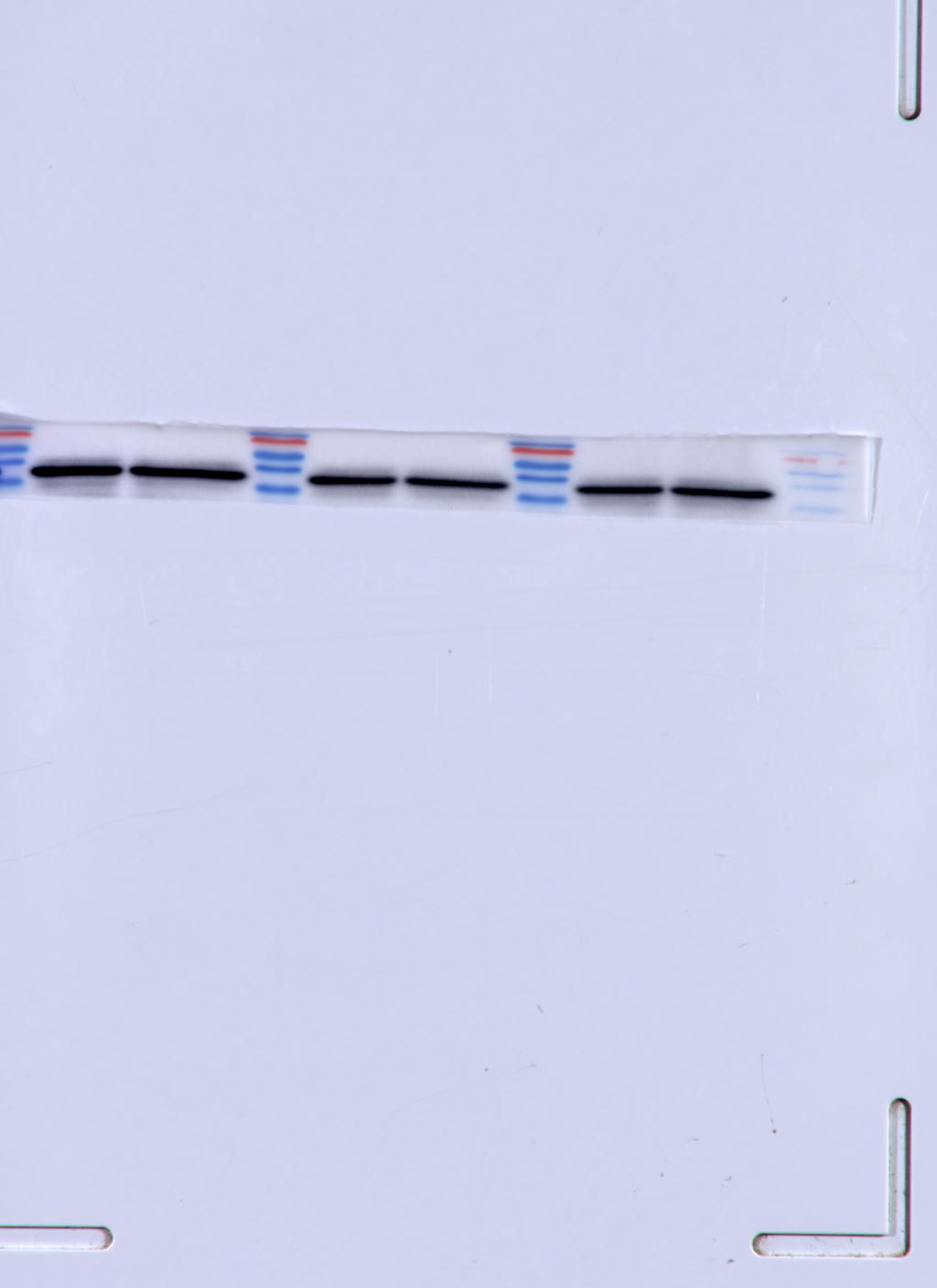

Supplement: Supplementary file 1 [file biology-11-01464-s001.zip › WB FIG/GAP/h1-gap 2022.05.14_17.56.52_Ch+Marker.jpg]

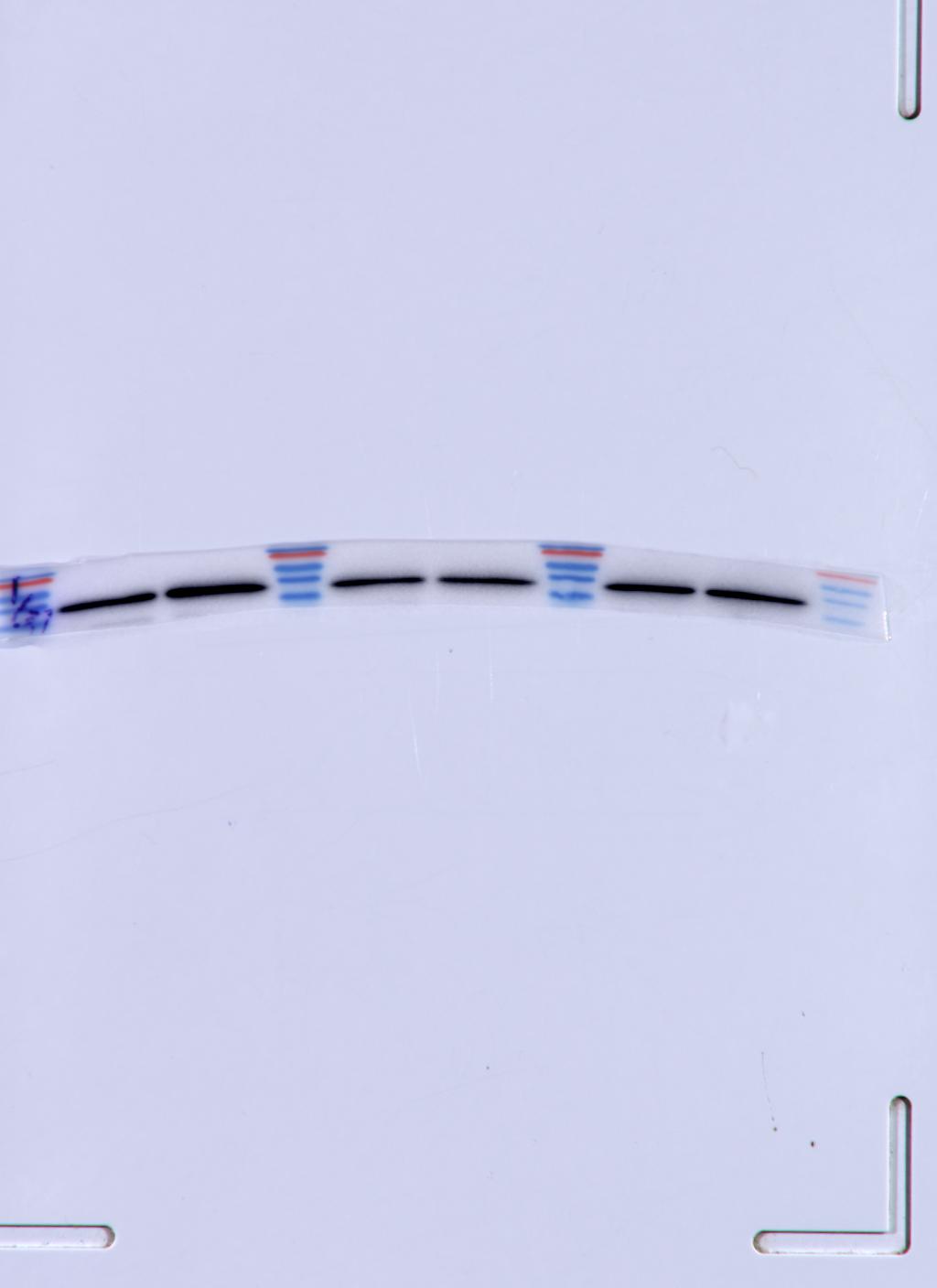

Supplement: Supplementary file 1 [file biology-11-01464-s001.zip › WB FIG/GAP/h1-gap 2022.05.18_17.17.20_Ch+Marker.jpg]

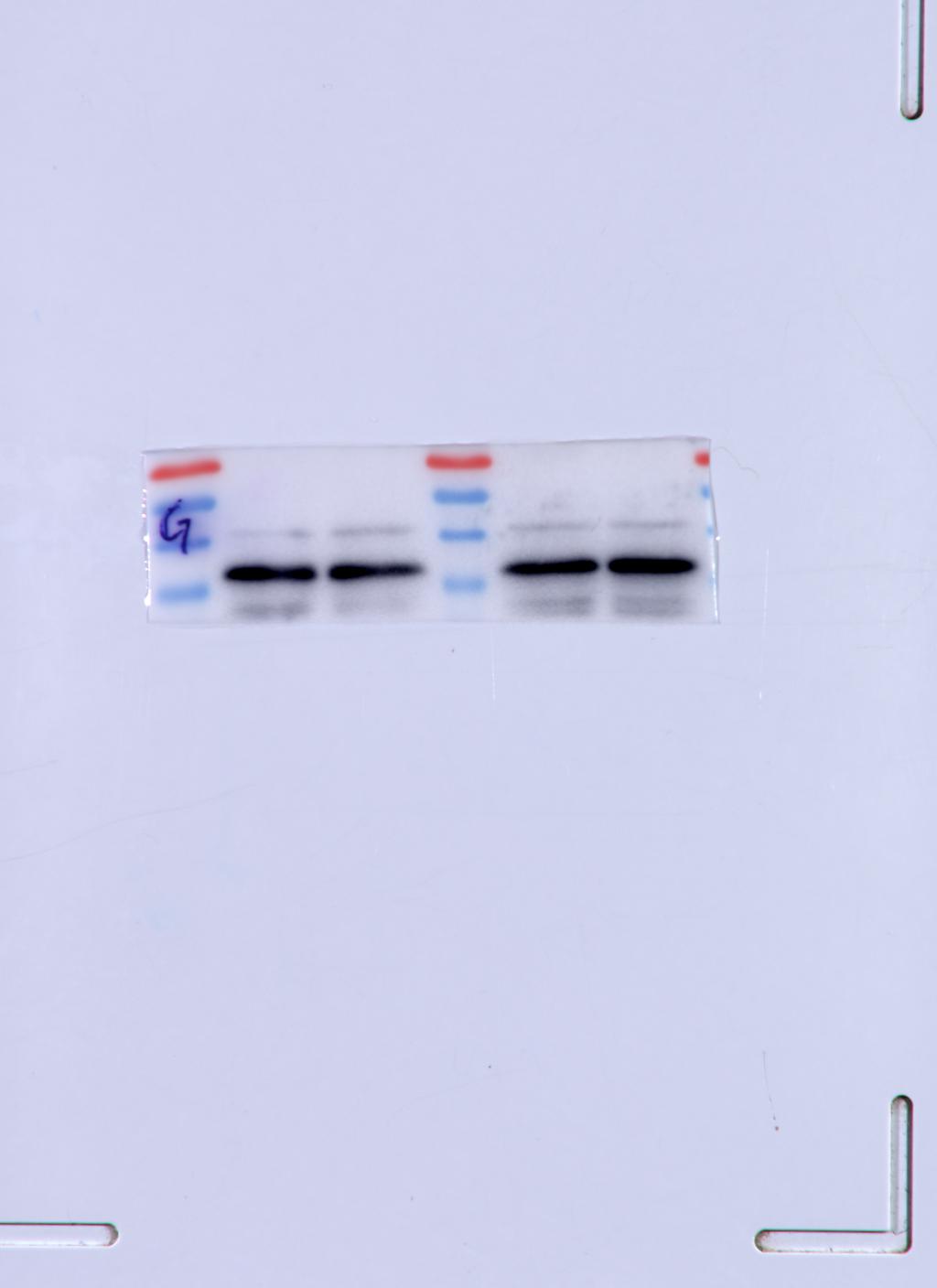

Supplement: Supplementary file 1 [file biology-11-01464-s001.zip › WB FIG/GAP/h1-h1&2-24h-gap 2022.04.26_18.19.54_Ch+Marker.jpg]

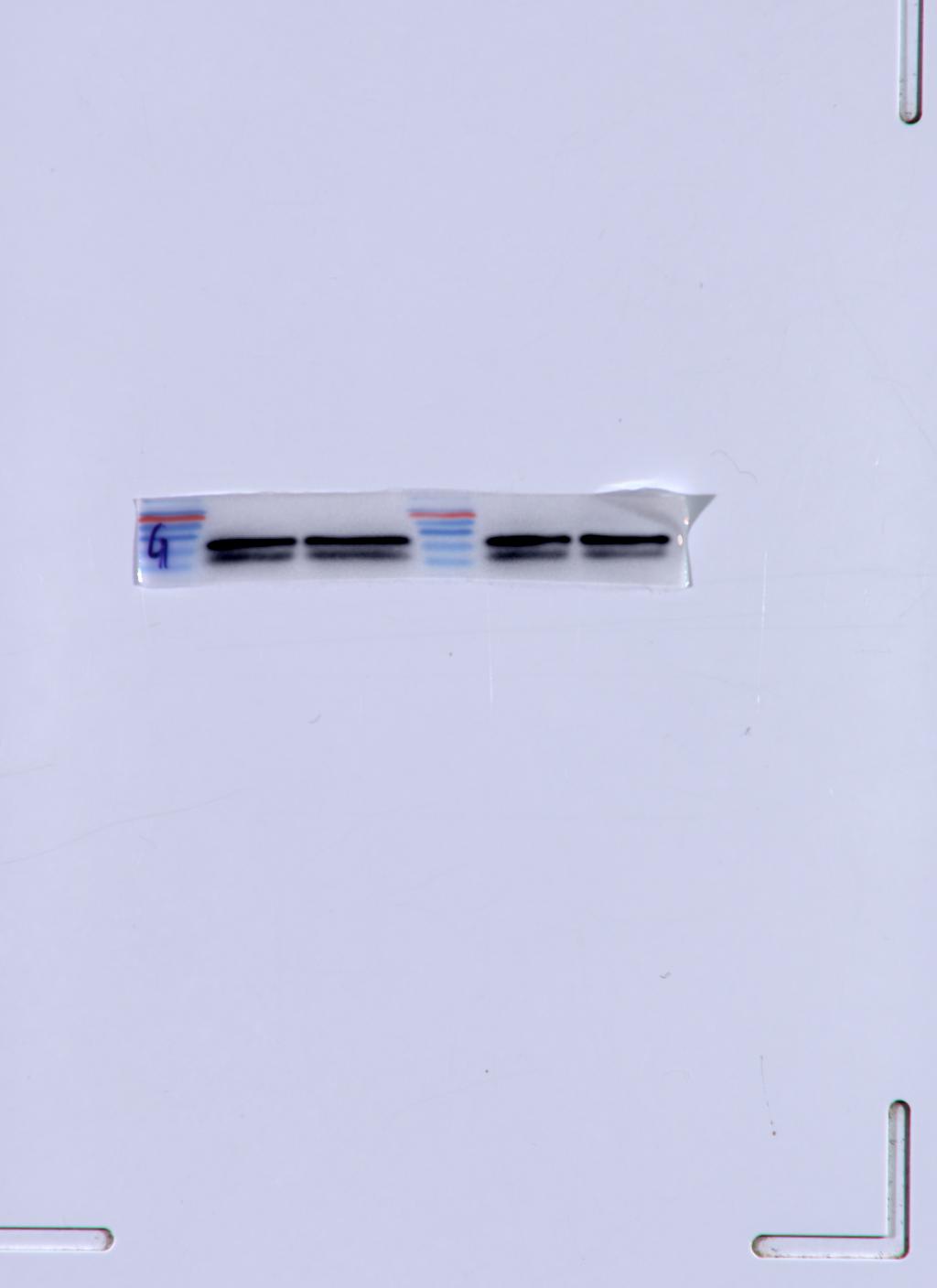

Supplement: Supplementary file 1 [file biology-11-01464-s001.zip › WB FIG/GAP/hdac1yizhiji-gap 2022.04.17_18.01.28_Ch+Marker.jpg]

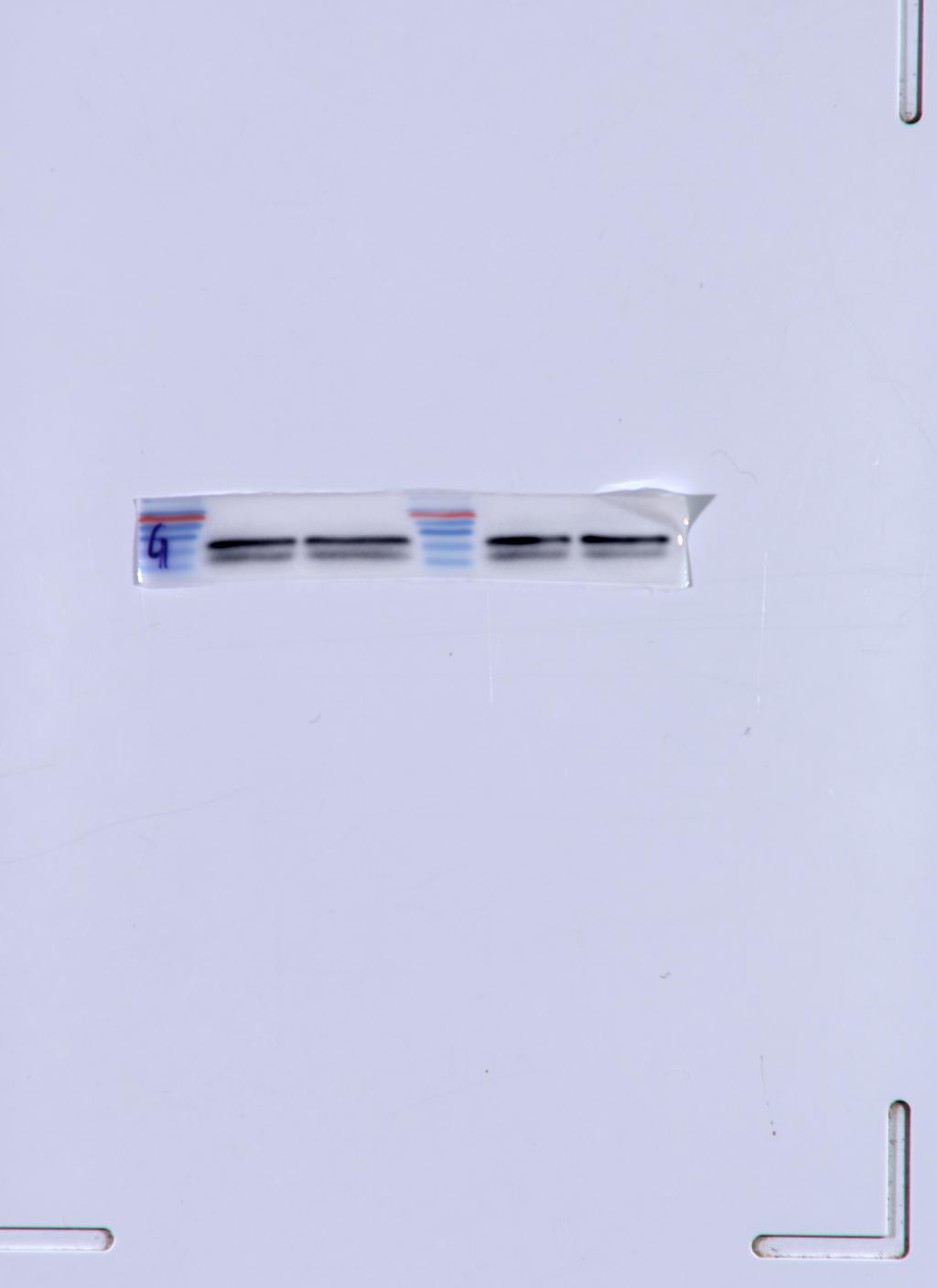

Supplement: Supplementary file 1 [file biology-11-01464-s001.zip › WB FIG/GAP/hdac1yizhiji-gap.1 2022.04.17_18.01.28_Ch+Marker.jpg]

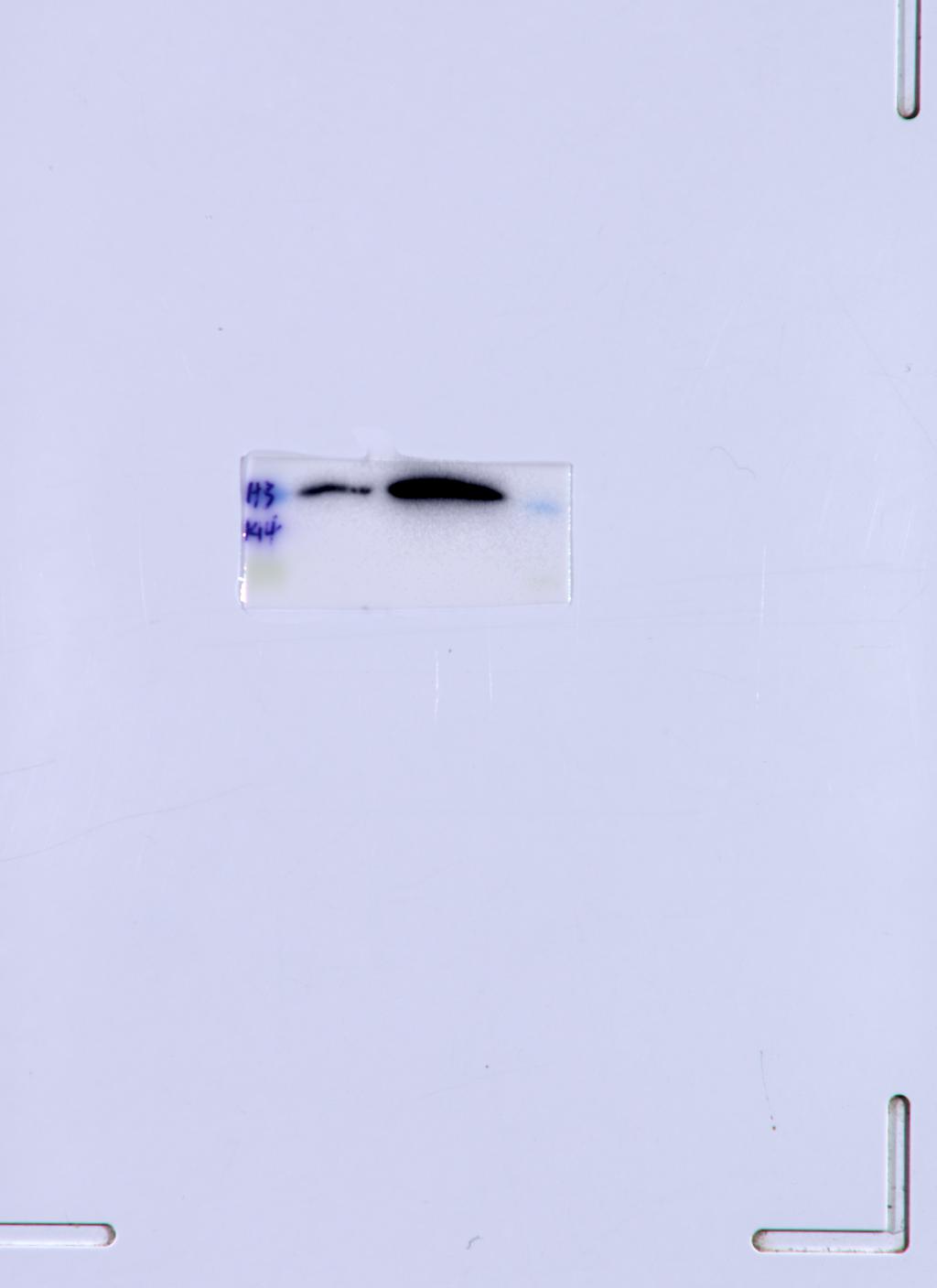

Supplement: Supplementary file 1 [file biology-11-01464-s001.zip › WB FIG/H3K14/h1-h3k14 2022.05.05_16.53.42_Ch+Marker.jpg]

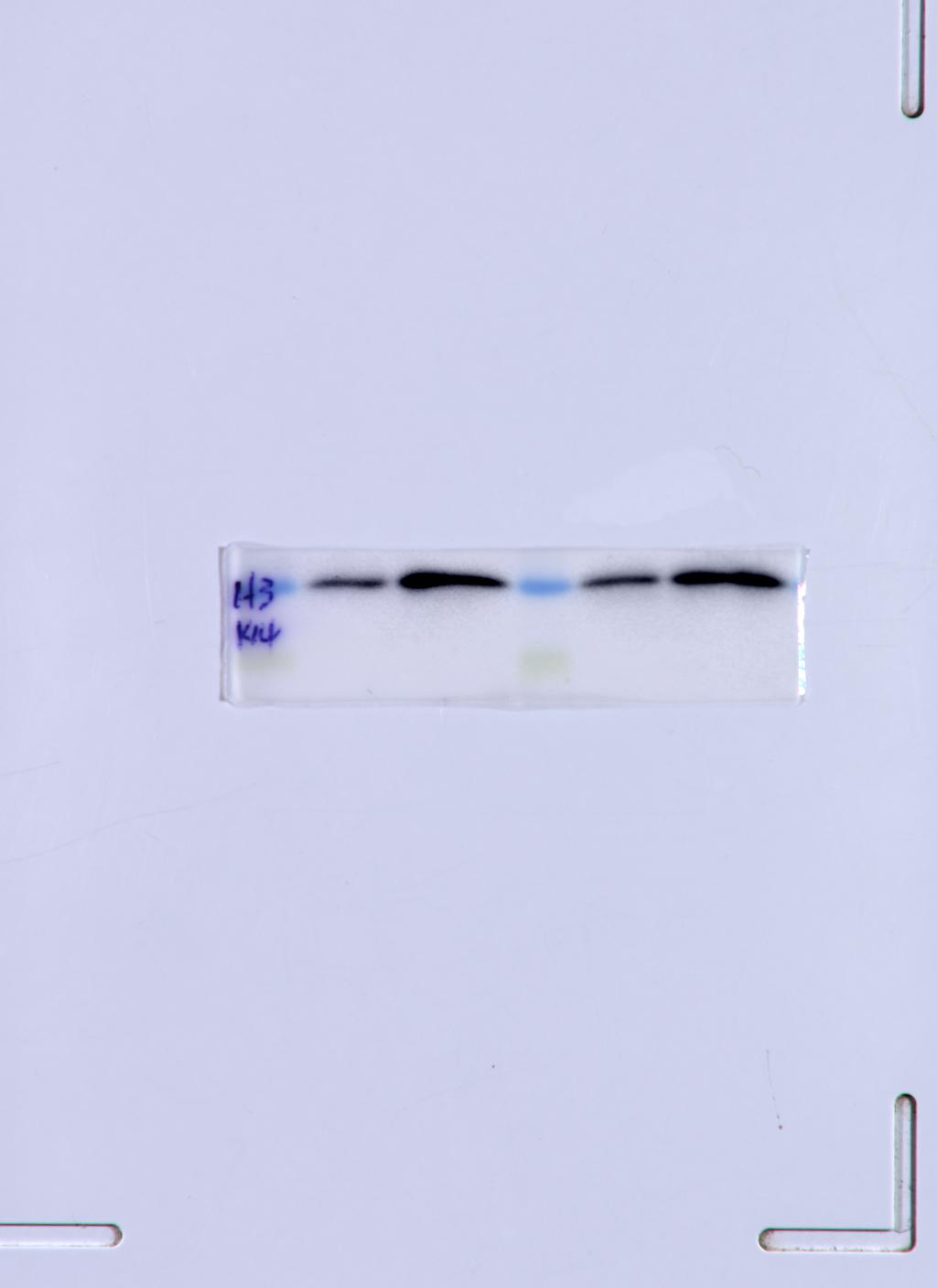

Supplement: Supplementary file 1 [file biology-11-01464-s001.zip › WB FIG/H3K14/h1-h3k14.1 2022.05.05_16.56.41_Ch+Marker.jpg]

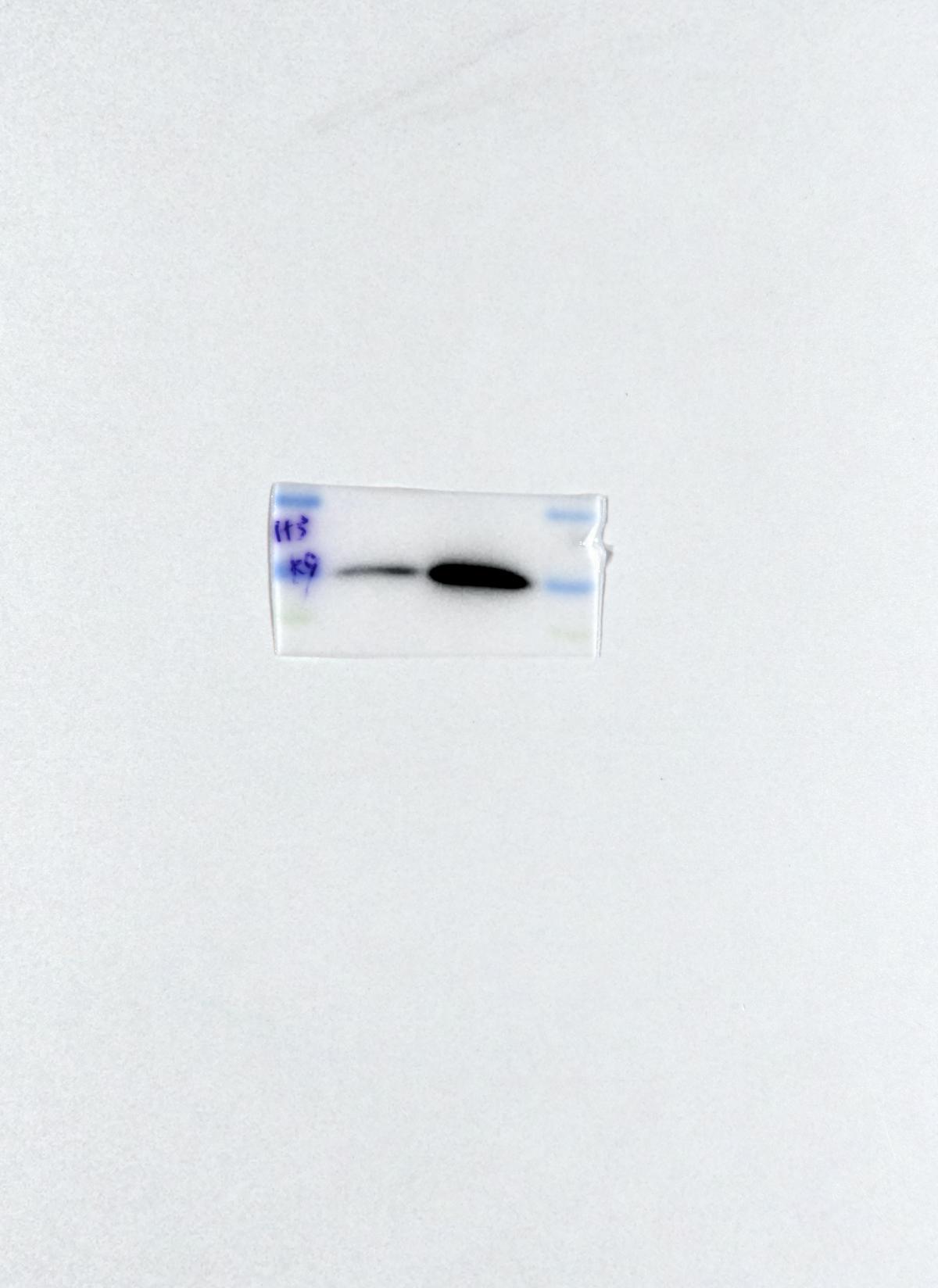

Supplement: Supplementary file 1 [file biology-11-01464-s001.zip › WB FIG/H3K9/hdac1.h3k9 20220607_095331_Ch_Chemi+Marker.jpg]

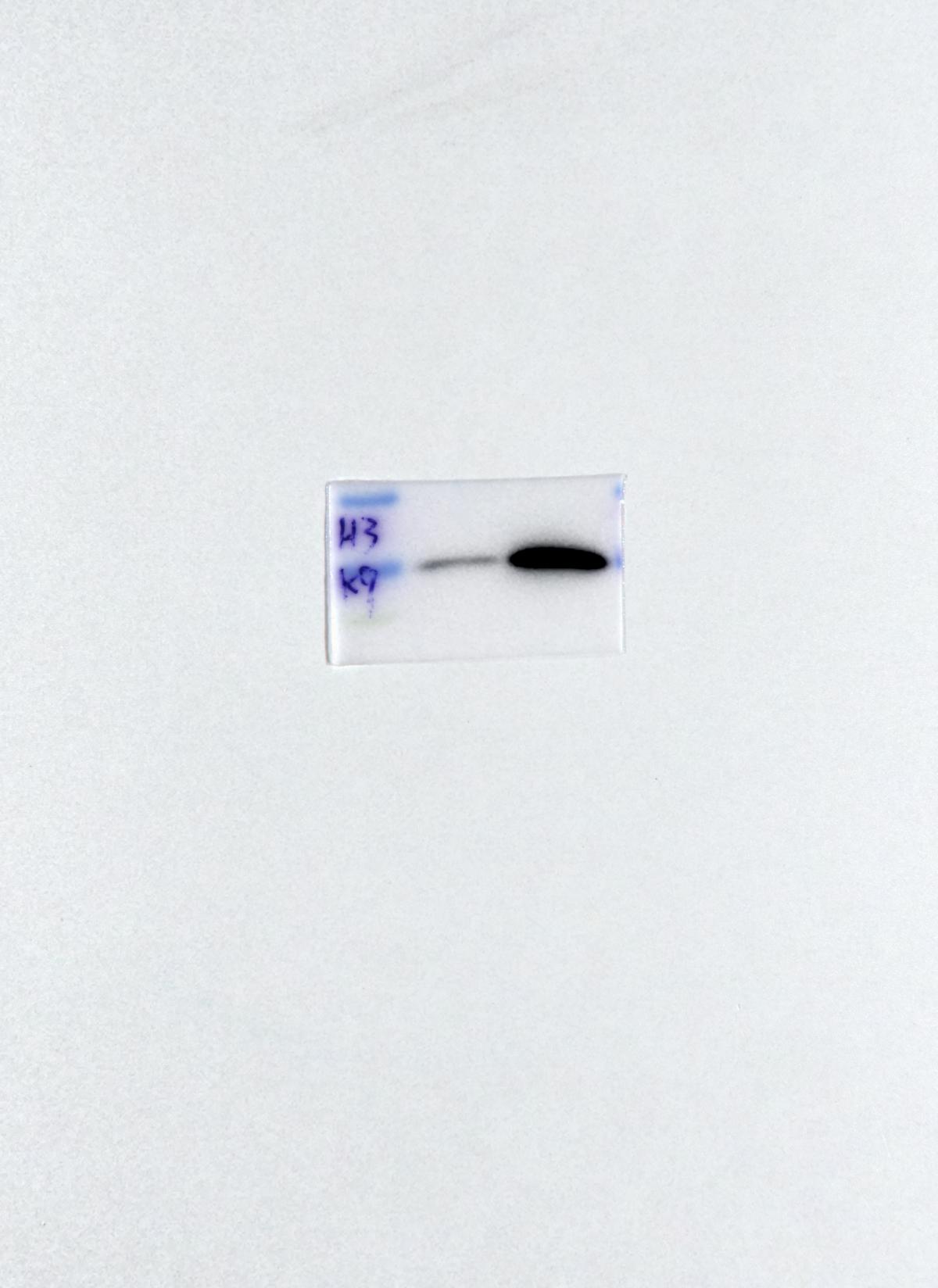

Supplement: Supplementary file 1 [file biology-11-01464-s001.zip › WB FIG/H3K9/hdac1.h3k9 20220607_095551_Ch_Chemi+Marker.jpg]

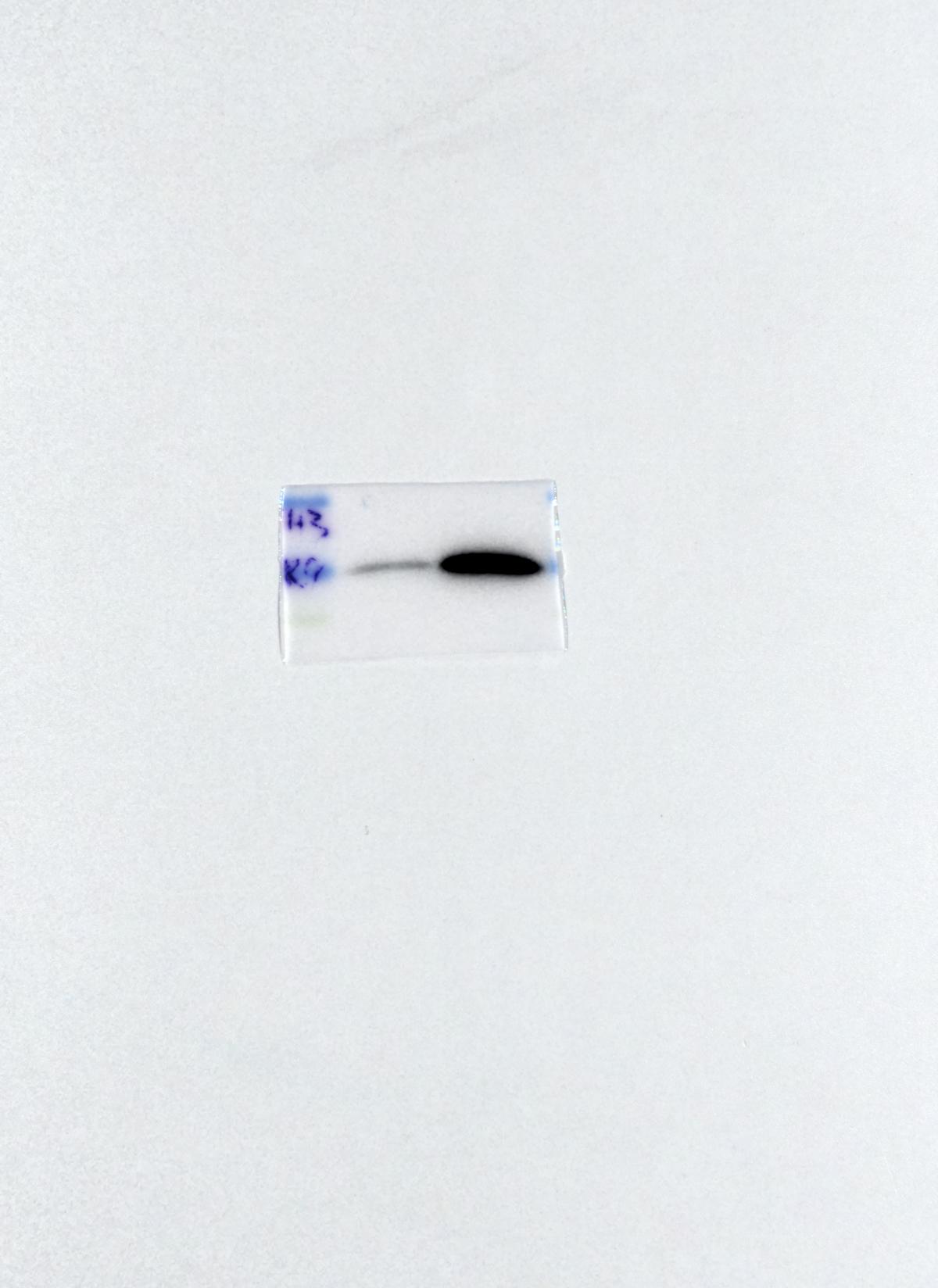

Supplement: Supplementary file 1 [file biology-11-01464-s001.zip › WB FIG/H3K9/hdac1.h3k9 20220607_095749_Ch_Chemi+Marker.jpg]

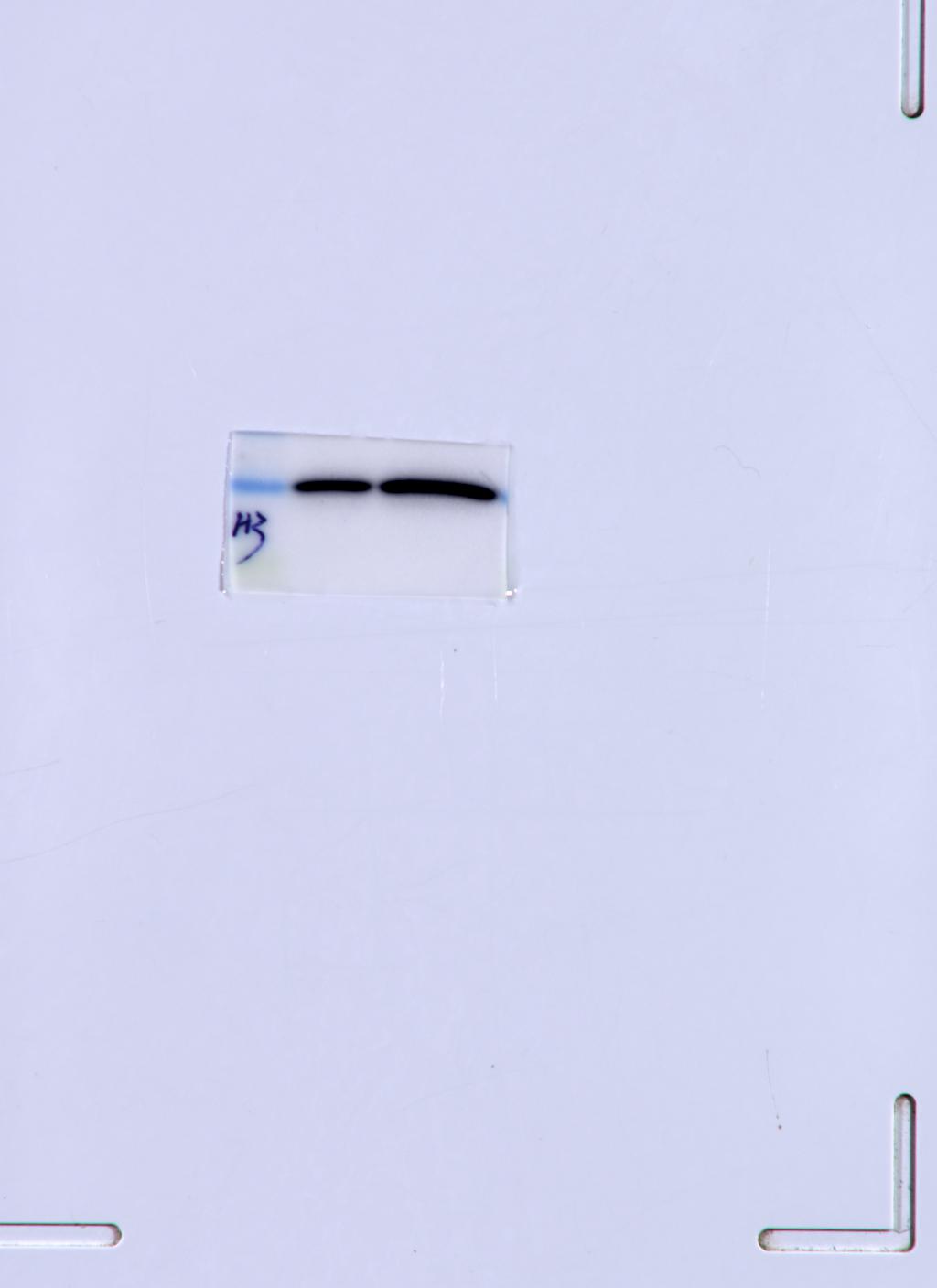

Supplement: Supplementary file 1 [file biology-11-01464-s001.zip › WB FIG/H3/h1-h3 2022.05.05_17.01.15_Ch+Marker.jpg]

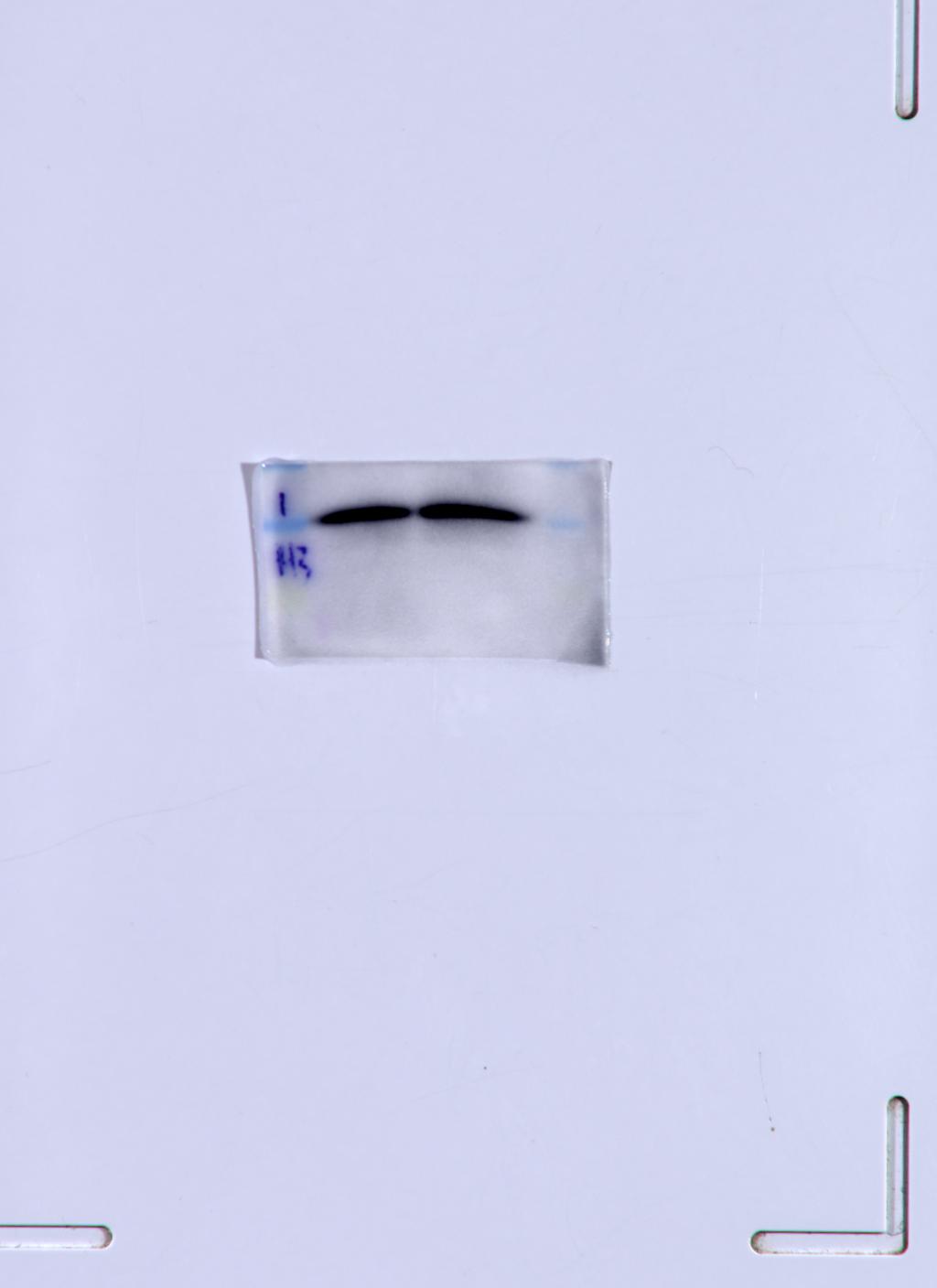

Supplement: Supplementary file 1 [file biology-11-01464-s001.zip › WB FIG/H3/h1-h3 2022.05.10_19.24.59_Ch+Marker.jpg]

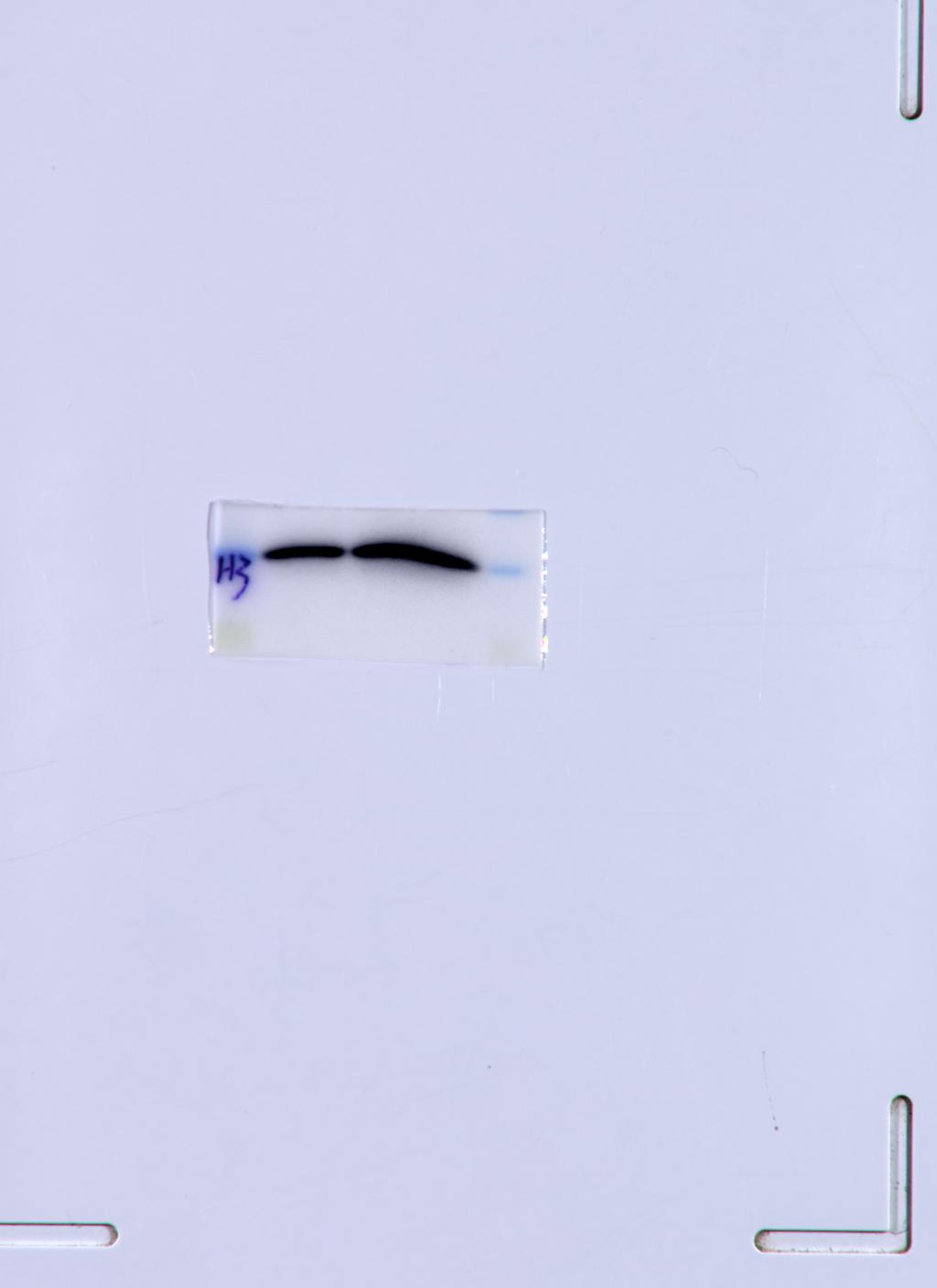

Supplement: Supplementary file 1 [file biology-11-01464-s001.zip › WB FIG/H3/h1-h3.1 2022.05.05_17.03.22_Ch+Marker.jpg]

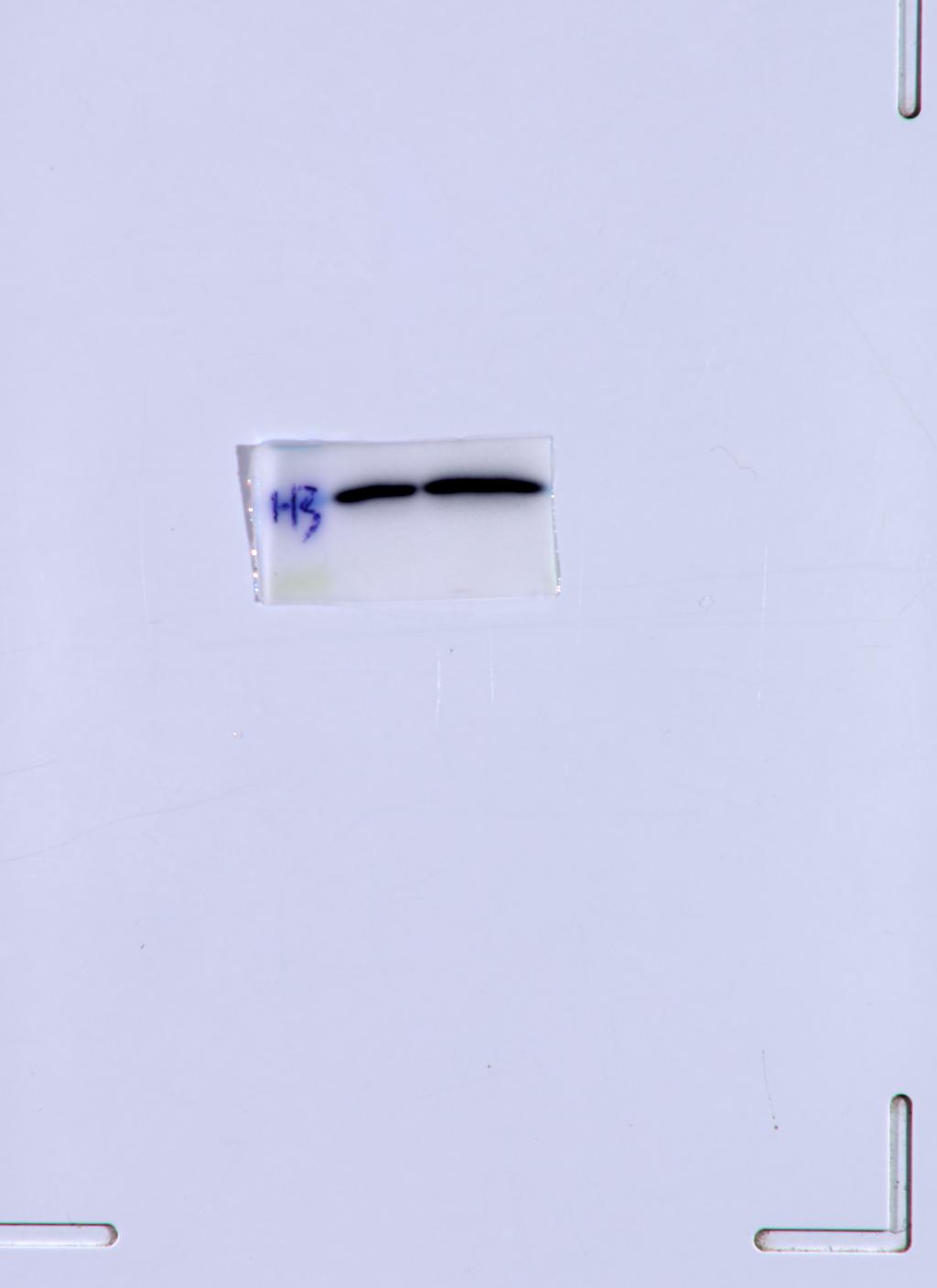

Supplement: Supplementary file 1 [file biology-11-01464-s001.zip › WB FIG/H3/h1-h3.2 2022.05.05_17.05.54_Ch+Marker.jpg]

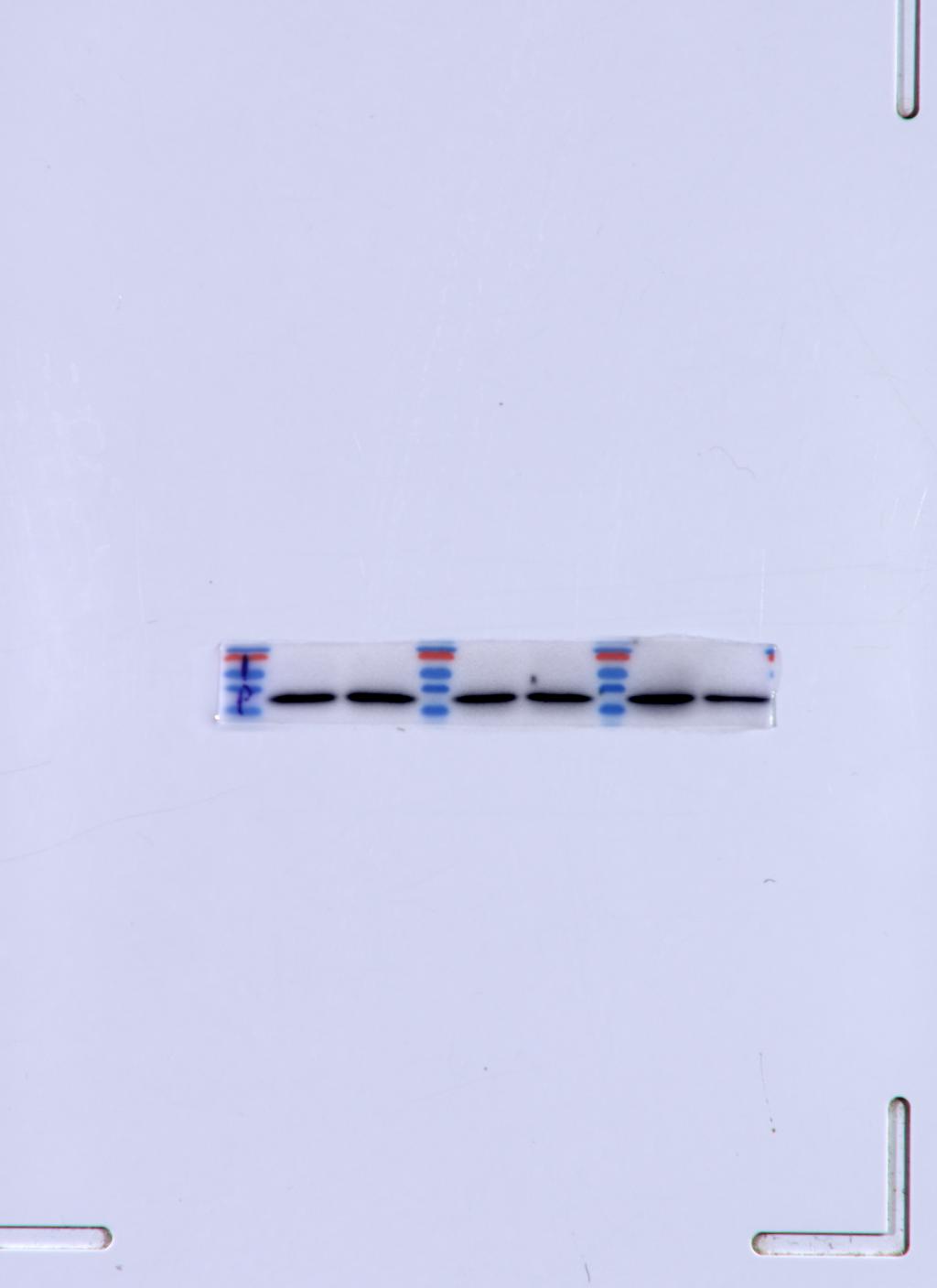

Supplement: Supplementary file 1 [file biology-11-01464-s001.zip › WB FIG/H3/h1-pcna 2022.05.14_18.34.51_Ch+Marker.jpg]

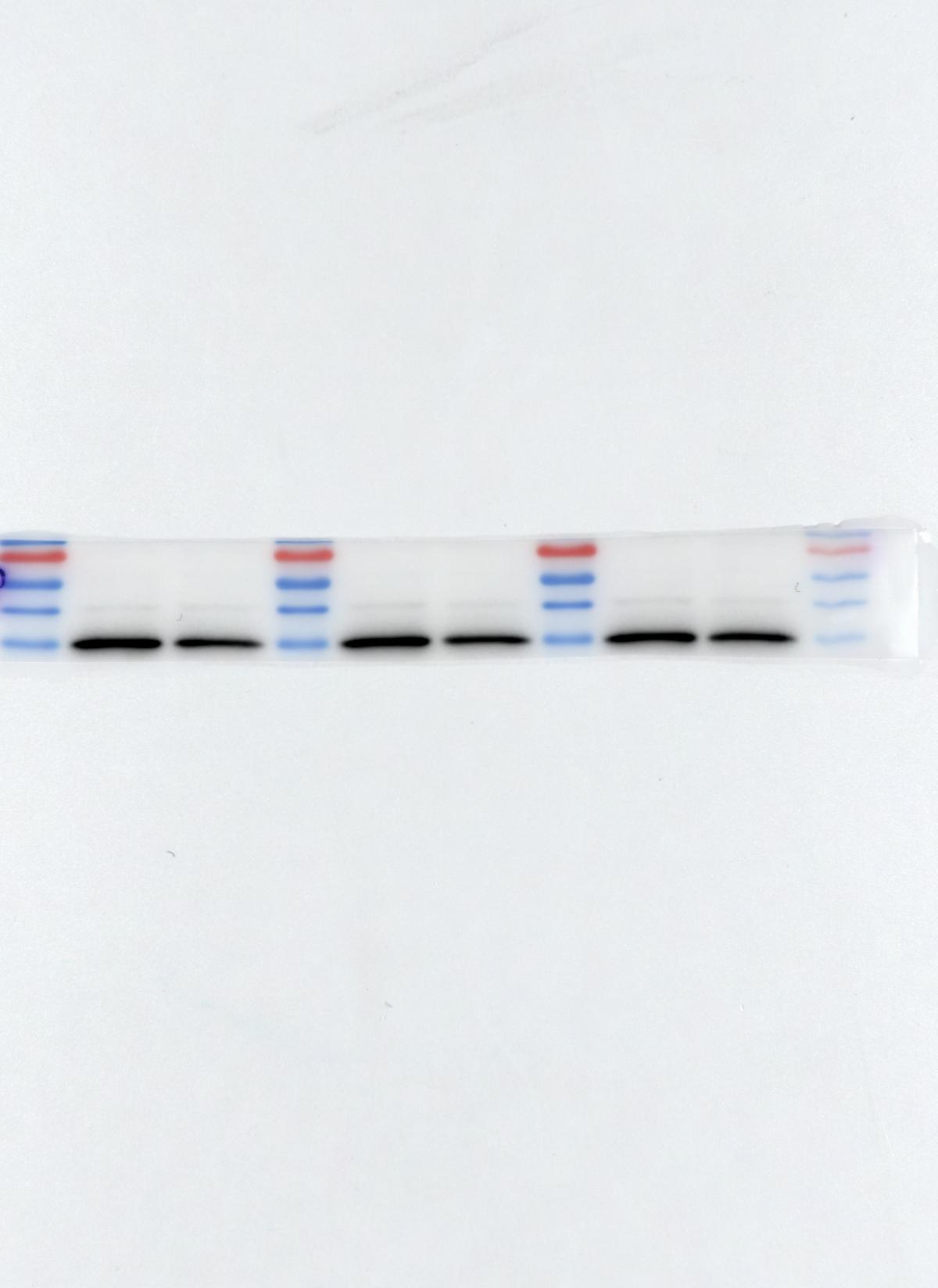

Supplement: Supplementary file 1 [file biology-11-01464-s001.zip › WB FIG/H3/hdac1.pcna 20220607_101336_Ch_Chemi+Marker.jpg]

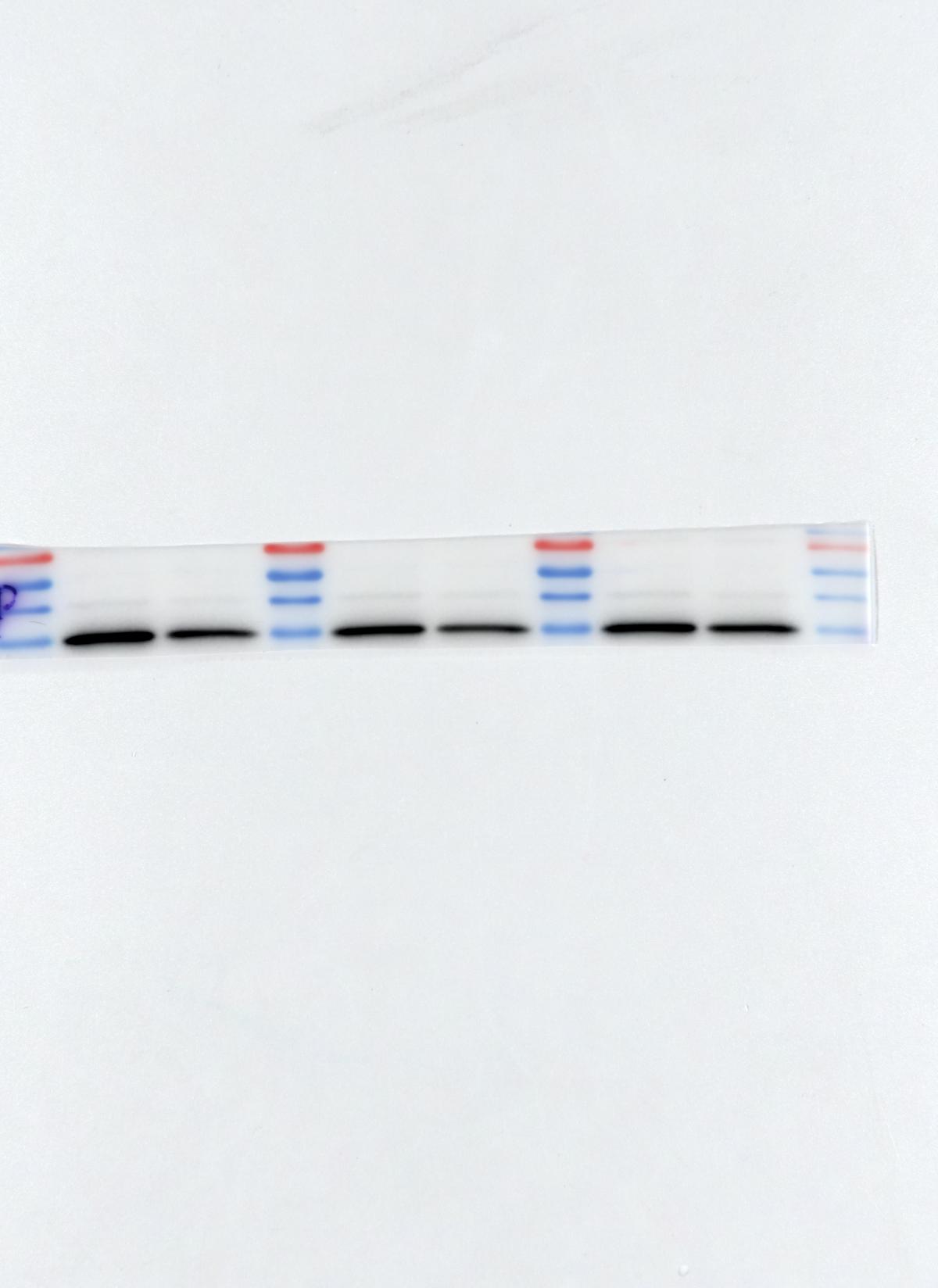

Supplement: Supplementary file 1 [file biology-11-01464-s001.zip › WB FIG/H3/hdac1.pcna 20220607_101539_Ch_Chemi+Marker.jpg]

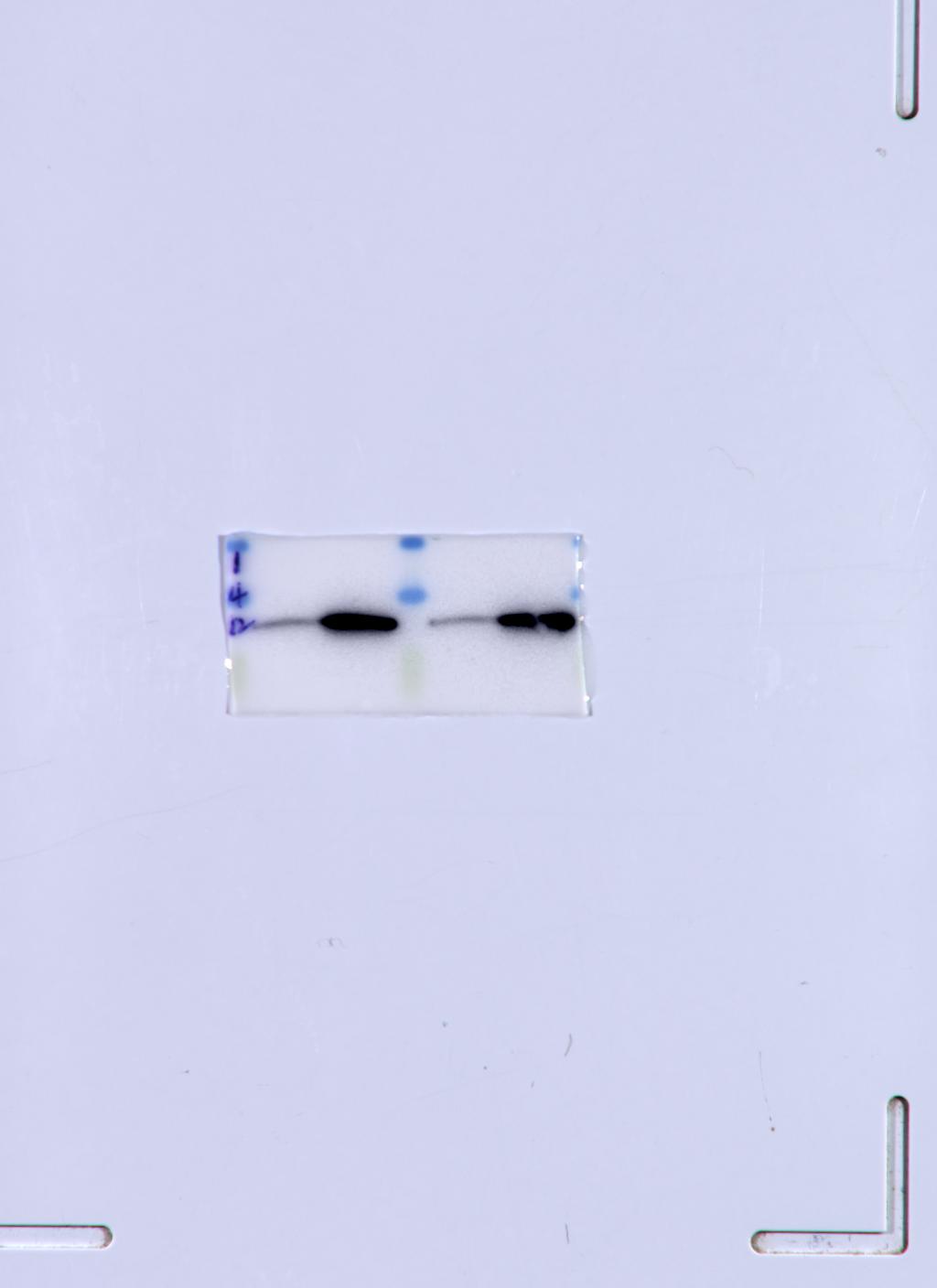

Supplement: Supplementary file 1 [file biology-11-01464-s001.zip › WB FIG/H4K12/h1-h4k12 2022.05.14_18.27.46_Ch+Marker.jpg]

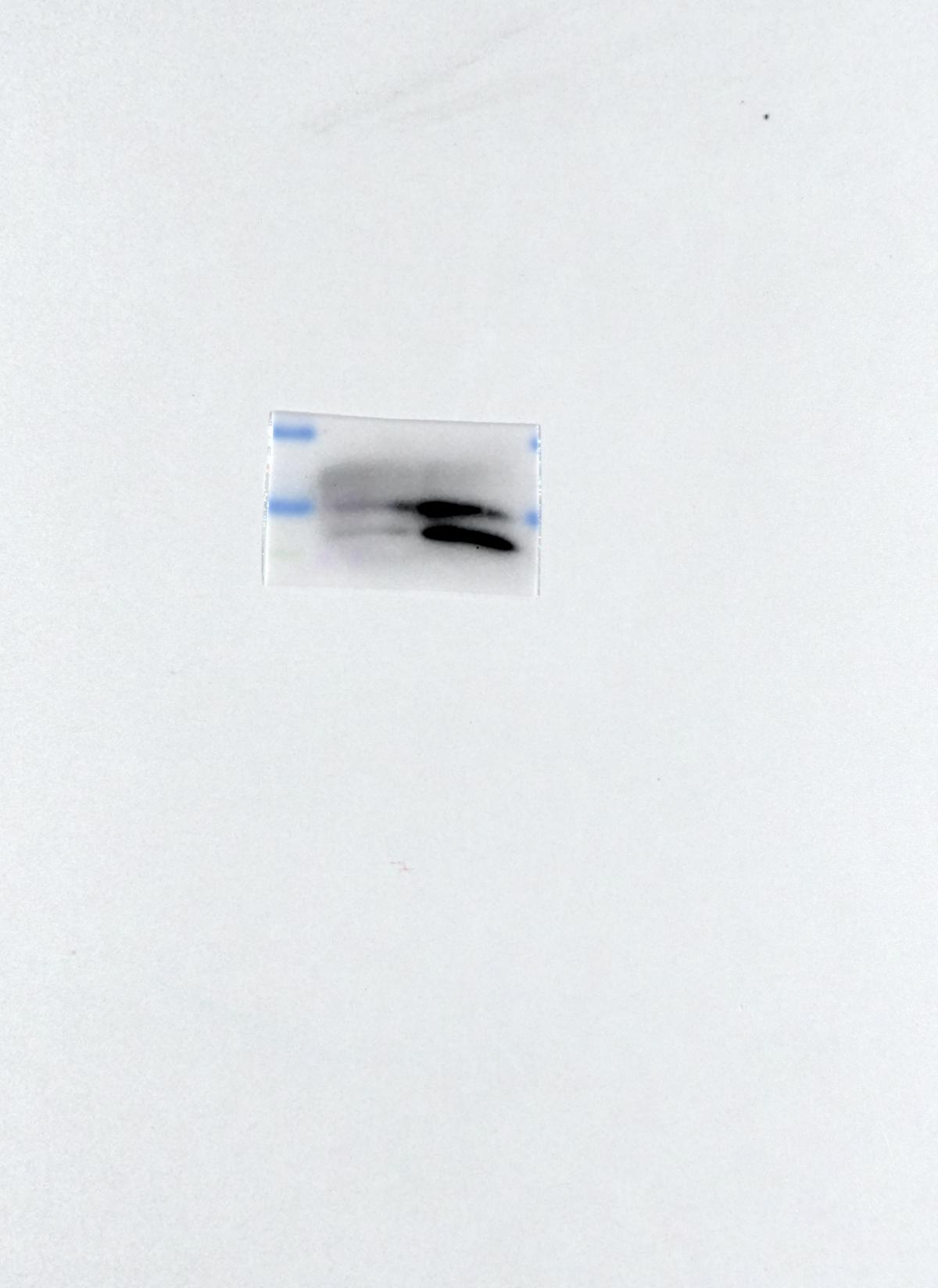

Supplement: Supplementary file 1 [file biology-11-01464-s001.zip › WB FIG/H4K12/hdac1.h4k12 20220607_100603_Ch_Chemi+Marker.jpg]

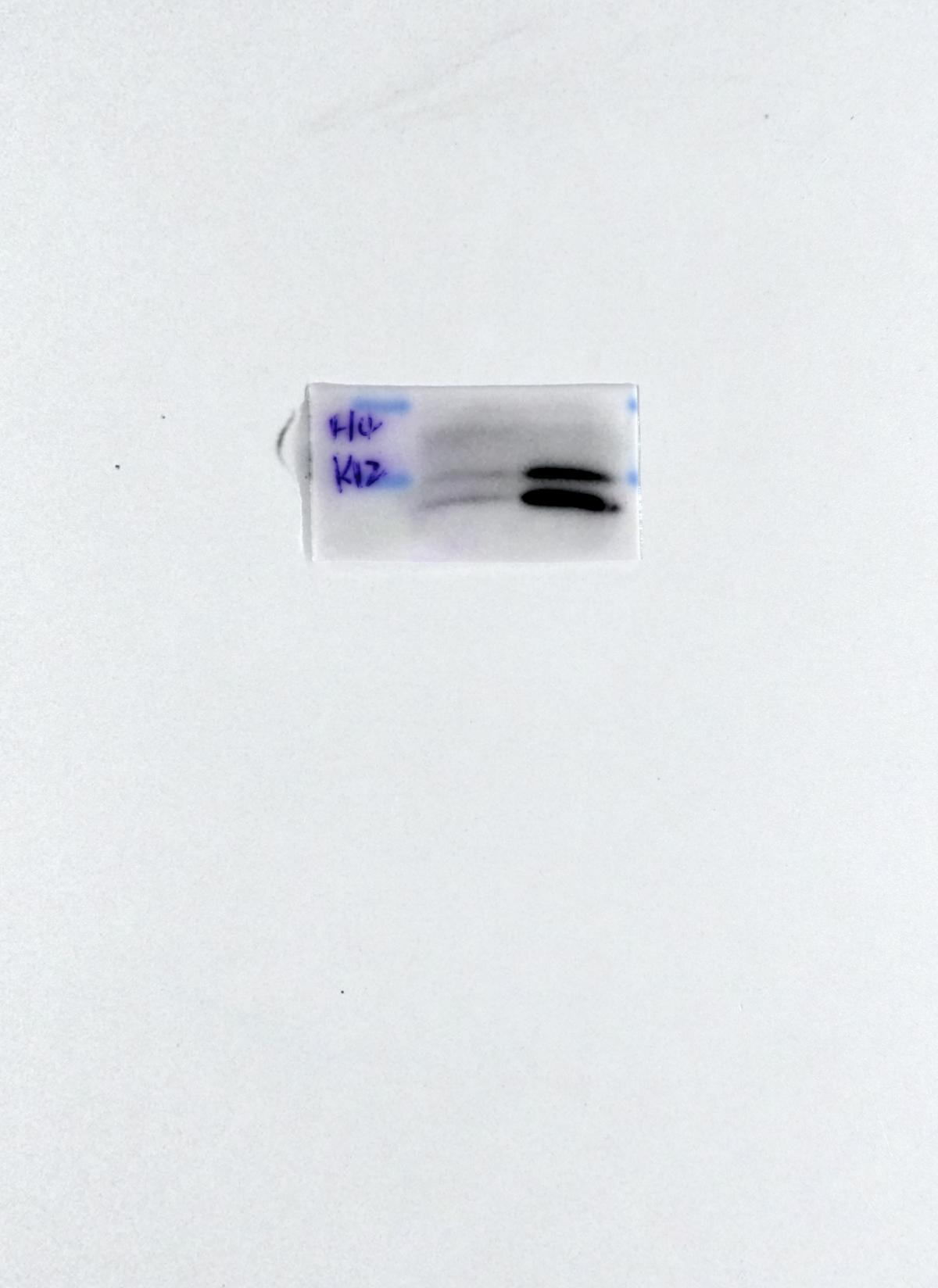

Supplement: Supplementary file 1 [file biology-11-01464-s001.zip › WB FIG/H4K12/hdac1.h4k12 20220607_100947_Ch_Chemi+Marker.jpg]

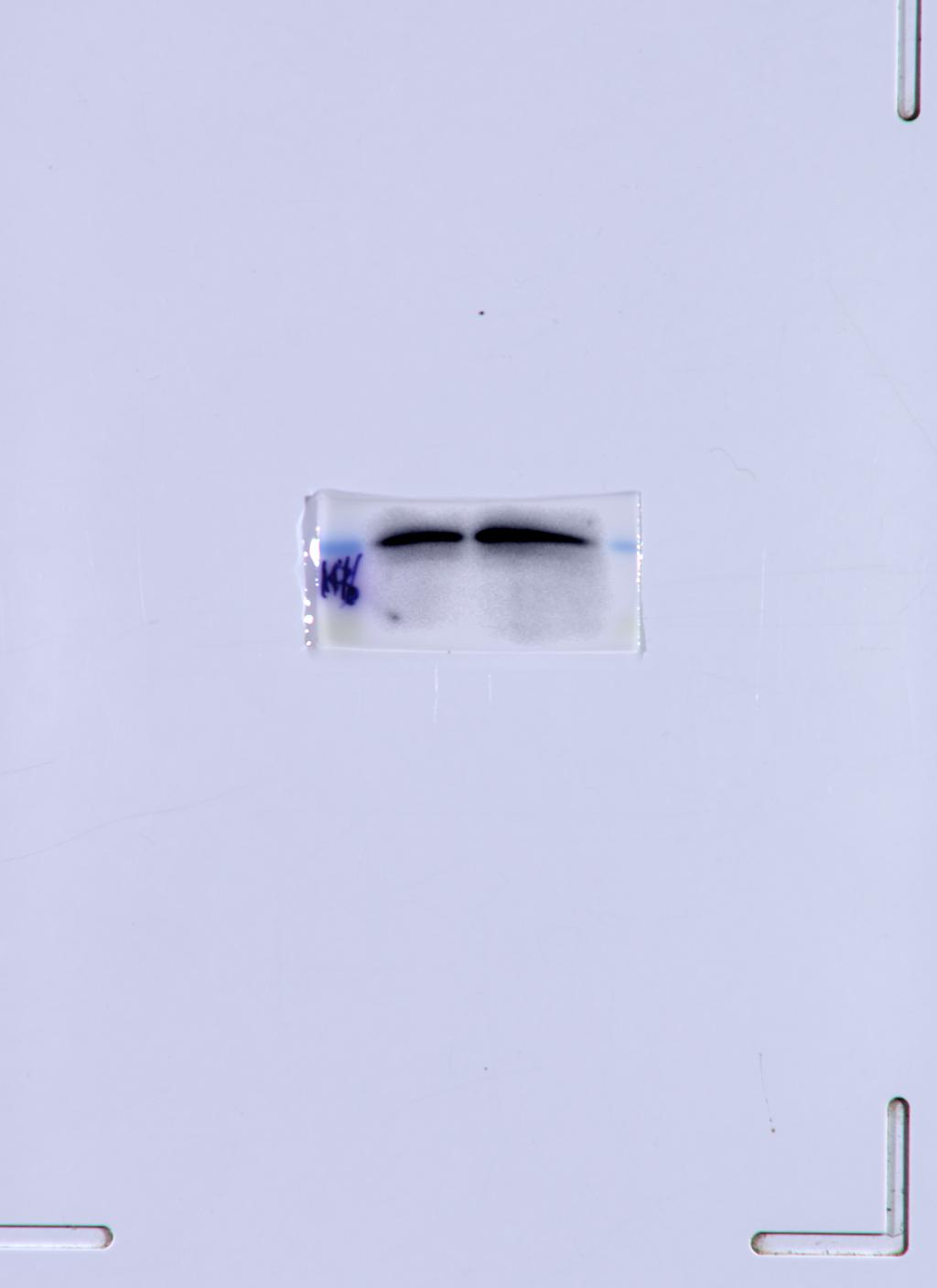

Supplement: Supplementary file 1 [file biology-11-01464-s001.zip › WB FIG/H4K16/h1-h4k16 2022.05.05_16.51.07_Ch+Marker.jpg]

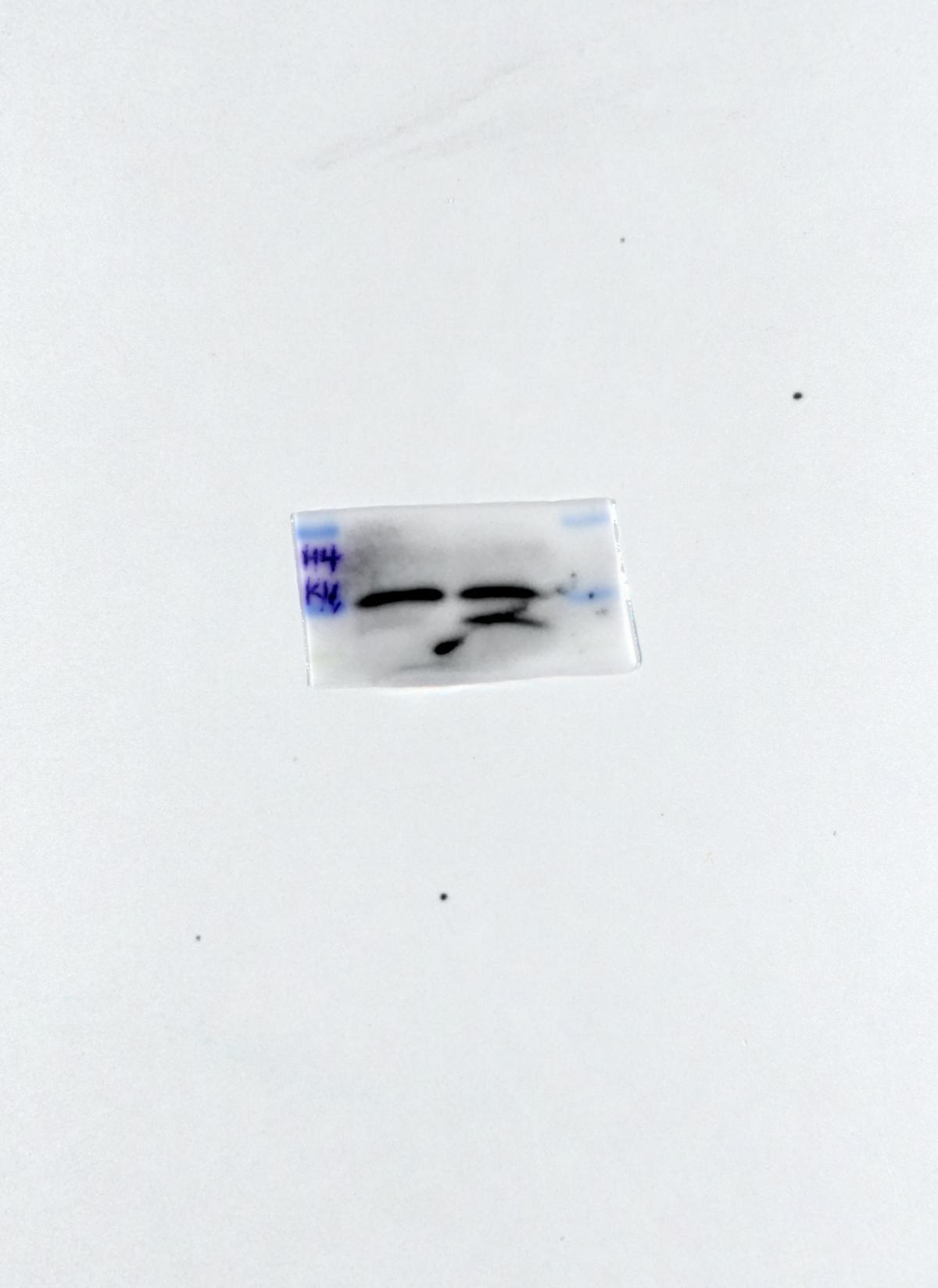

Supplement: Supplementary file 1 [file biology-11-01464-s001.zip › WB FIG/H4K16/hdac1.h4k16 20220607_095927_Ch_Chemi+Marker.jpg]

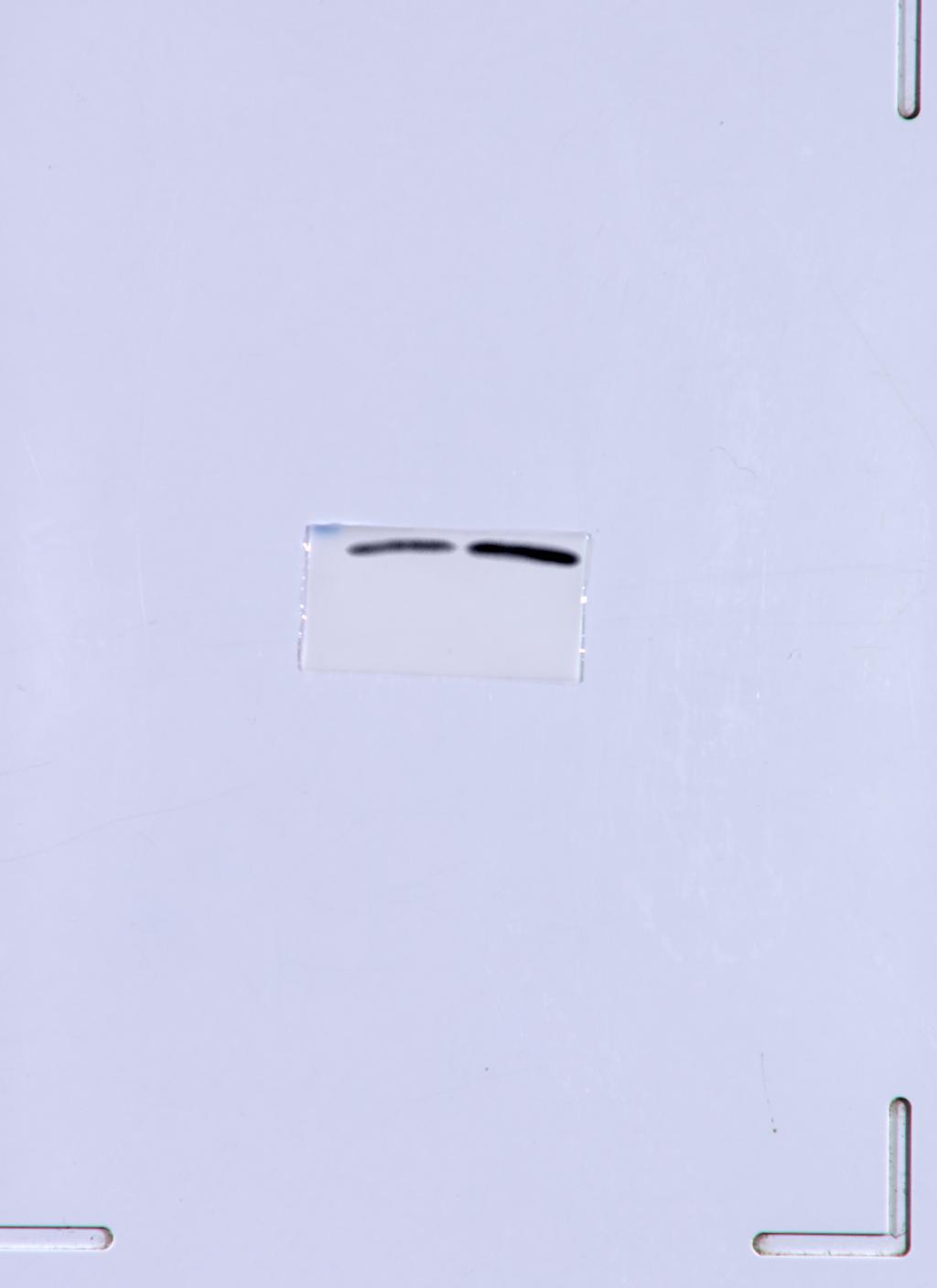

Supplement: Supplementary file 1 [file biology-11-01464-s001.zip › WB FIG/H4K5/h1-30ug-h4k5.2 2022.04.28_19.50.16_Ch+Marker.jpg]

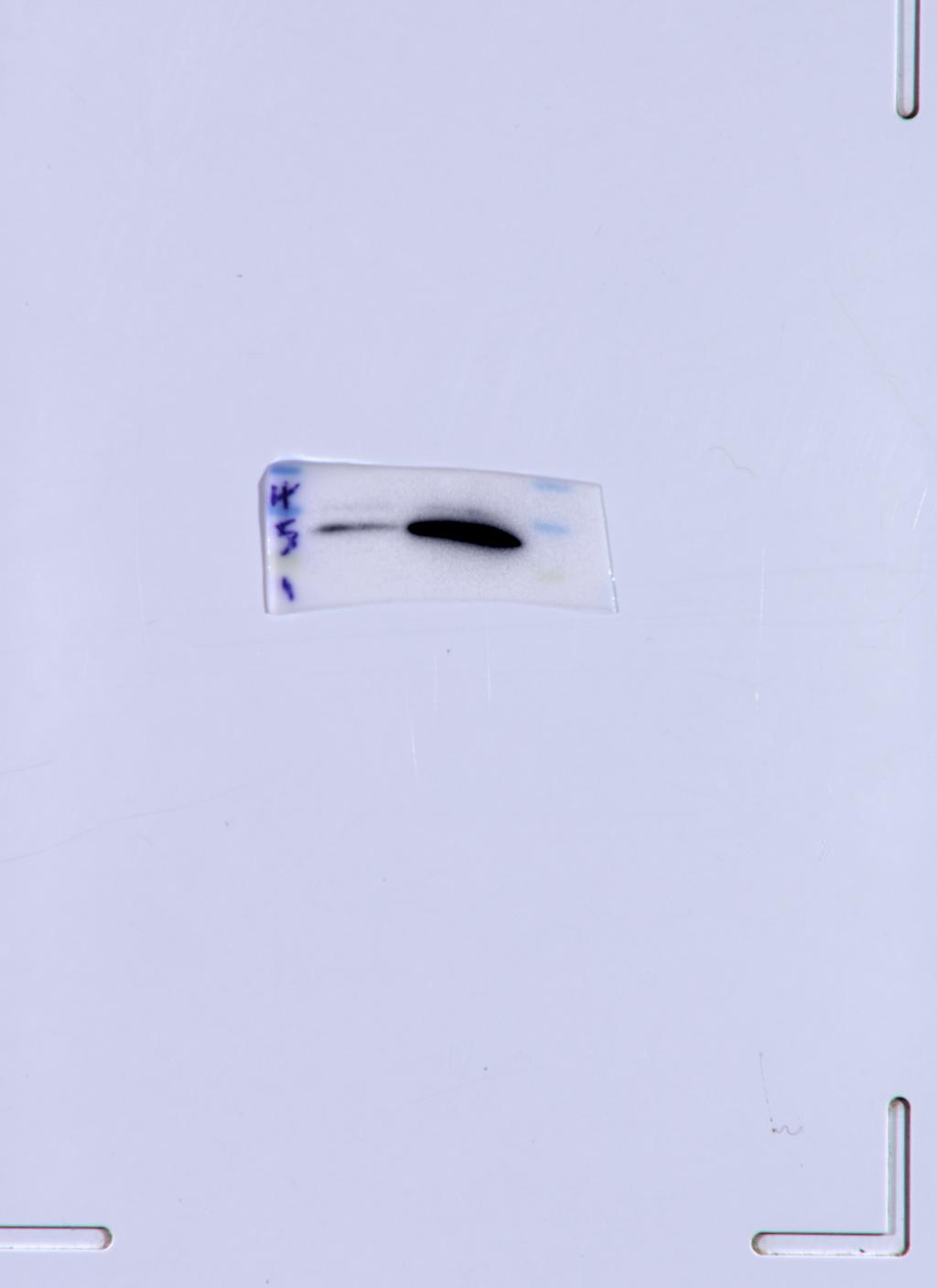

Supplement: Supplementary file 1 [file biology-11-01464-s001.zip › WB FIG/H4K5/h1-h4k5 2022.05.18_17.20.17_Ch+Marker.jpg]

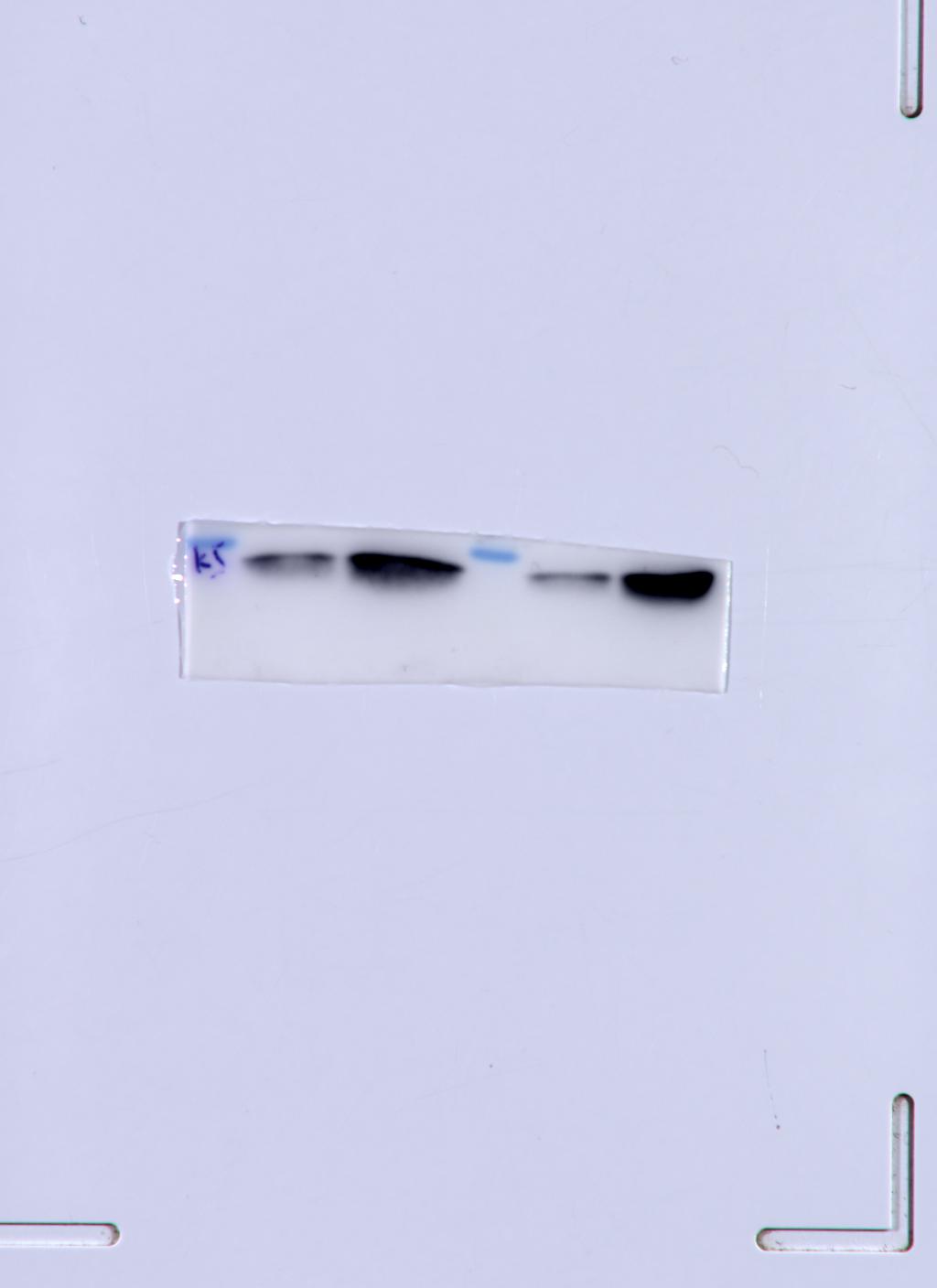

Supplement: Supplementary file 1 [file biology-11-01464-s001.zip › WB FIG/H4K5/hdac1yizhiji-h4k5 2022.04.17_18.08.40_Ch+Marker.jpg]

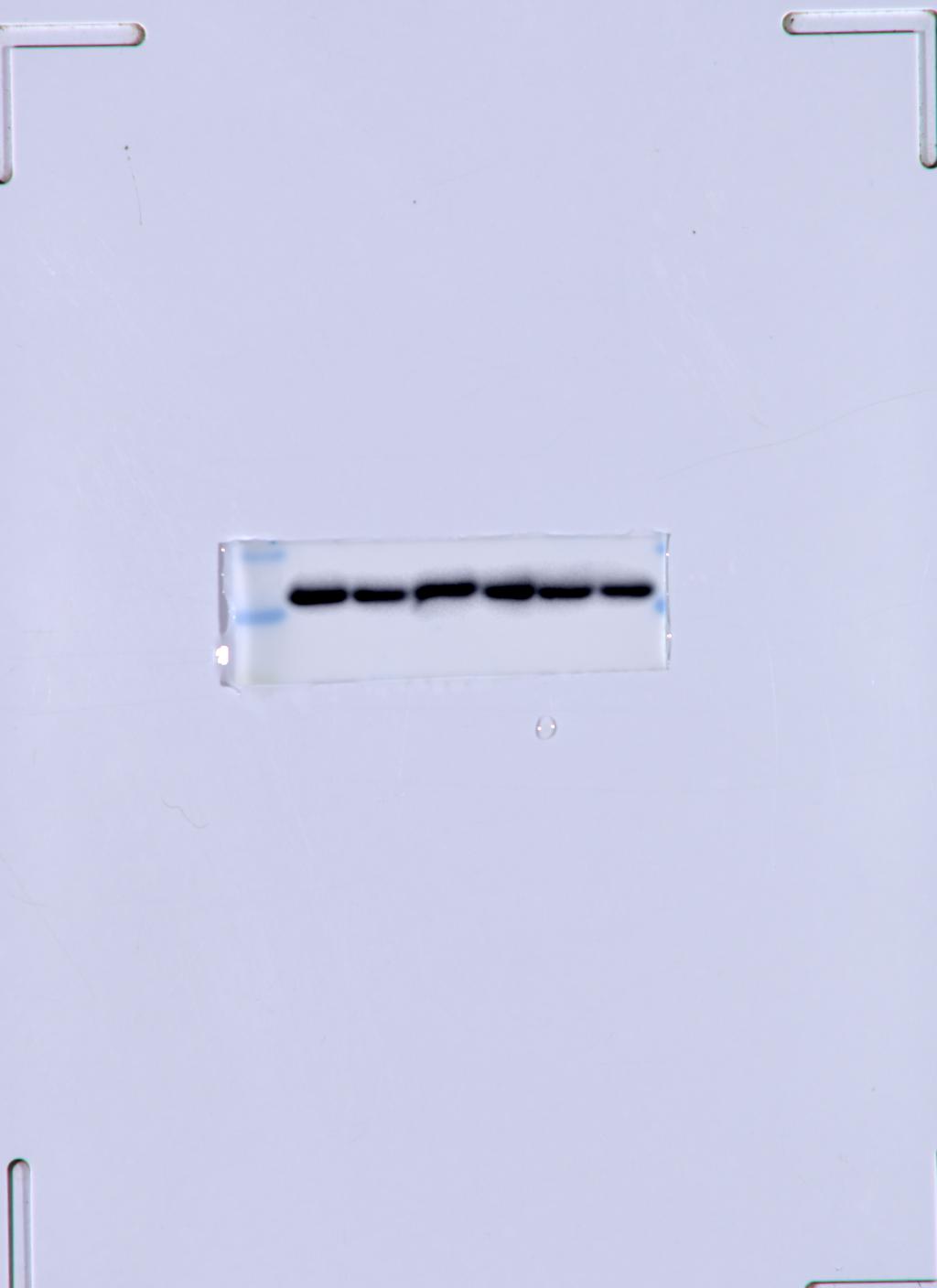

Supplement: Supplementary file 1 [file biology-11-01464-s001.zip › WB FIG/LH/LH-H3 2021.12.19_16.58.49_Ch+Marker.jpg]

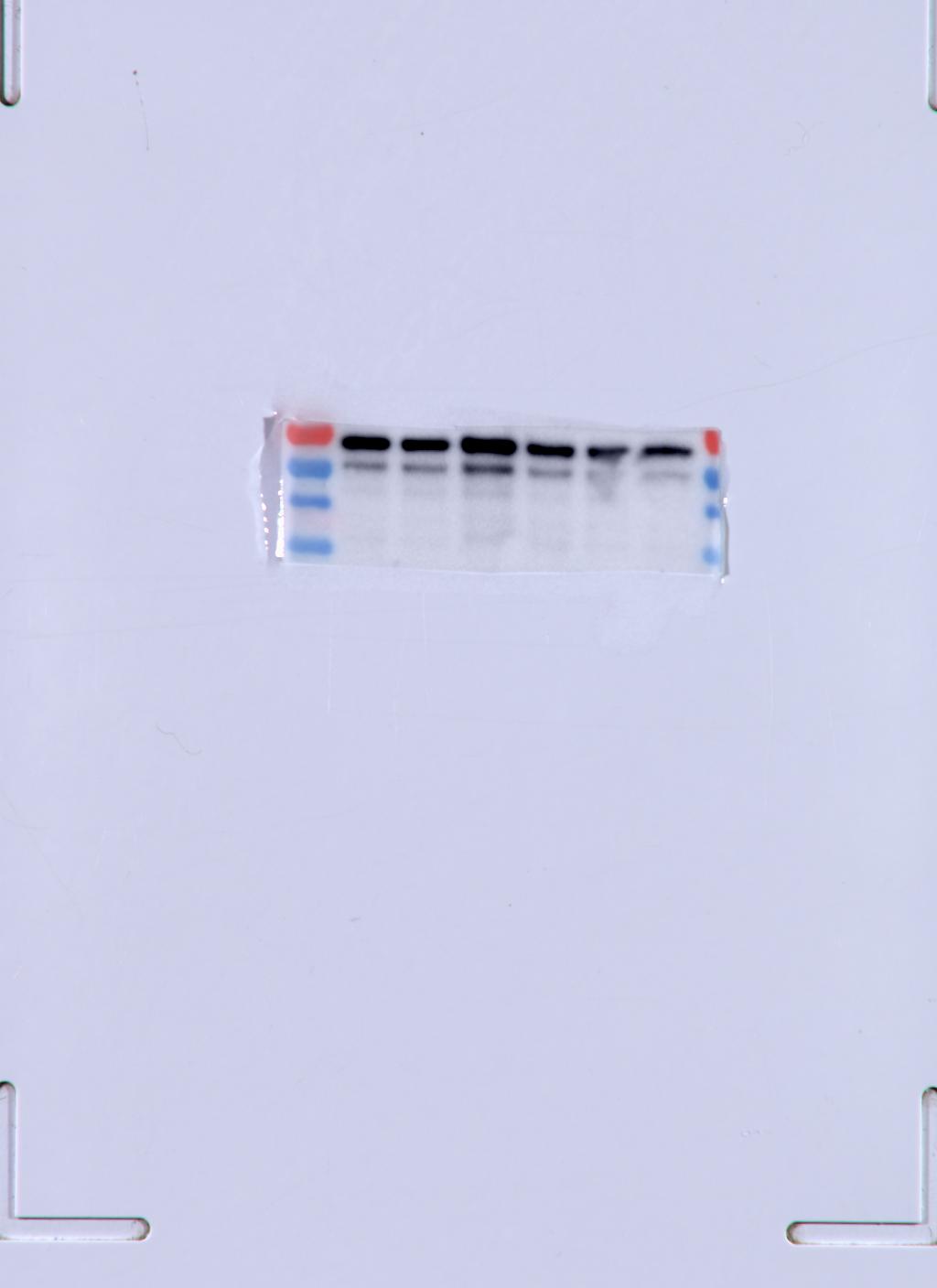

Supplement: Supplementary file 1 [file biology-11-01464-s001.zip › WB FIG/LH/lh1-8-hdac1 2021.12.21_17.26.31_Ch+Marker.jpg]

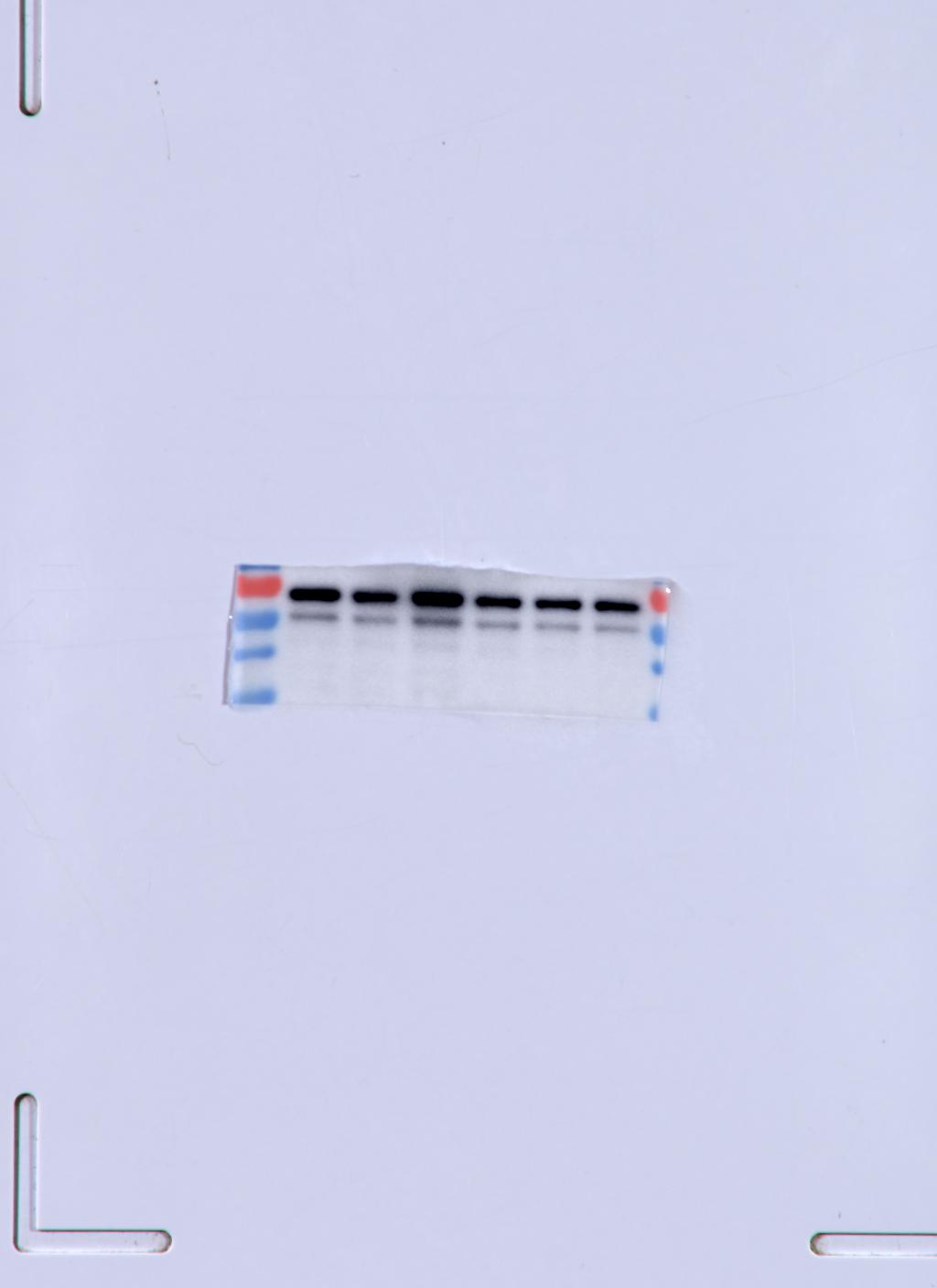

Supplement: Supplementary file 1 [file biology-11-01464-s001.zip › WB FIG/LH/lh1-8-hdac1- 2021.12.21_17.43.46_Ch+Marker.jpg]

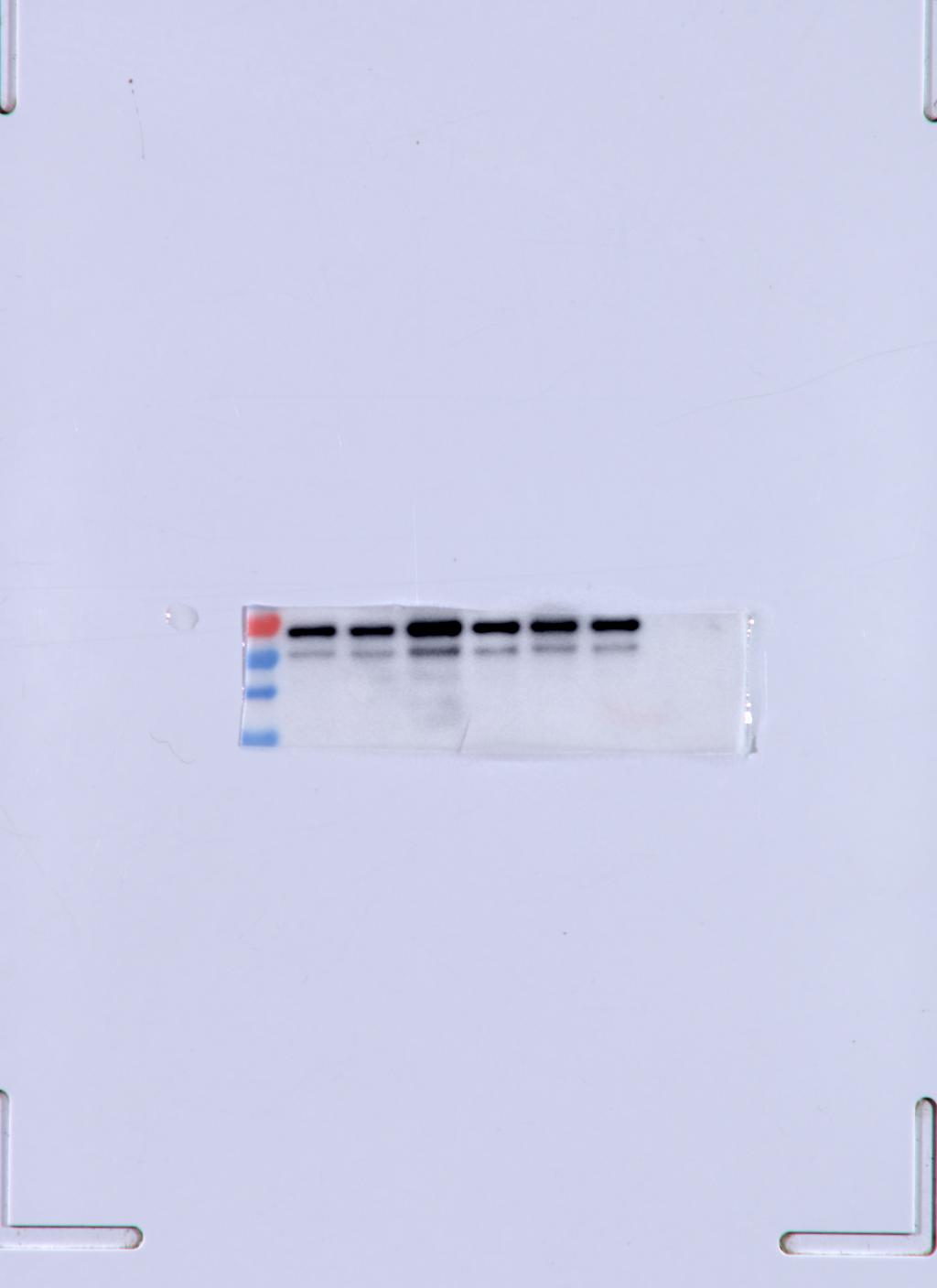

Supplement: Supplementary file 1 [file biology-11-01464-s001.zip › WB FIG/LH/lh1-8-hdac1-- 2021.12.21_17.52.47_Ch+Marker.jpg]

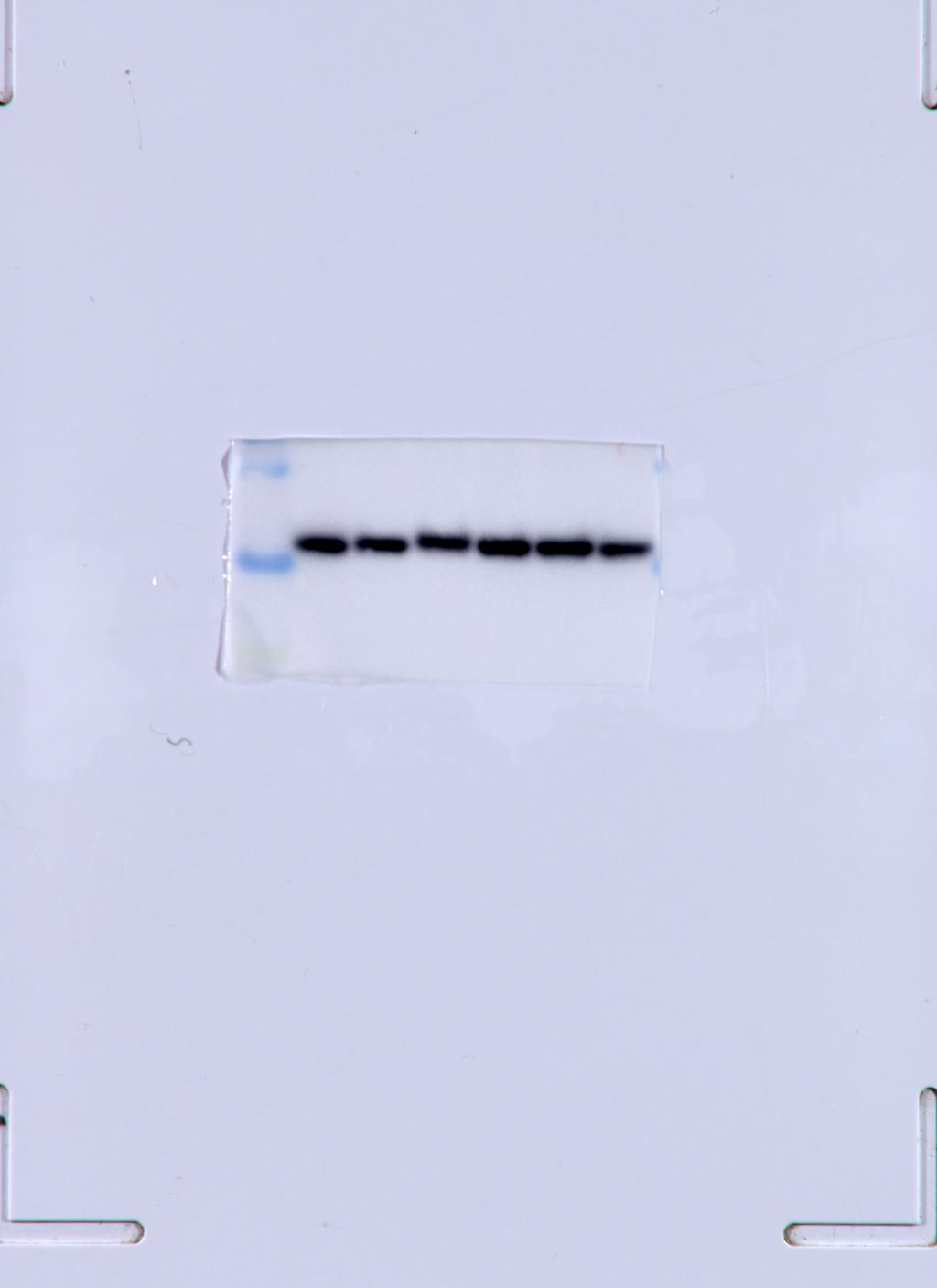

Supplement: Supplementary file 1 [file biology-11-01464-s001.zip › WB FIG/LH/lh1-8h-h3.1 2021.12.23_11.38.28_Ch+Marker.jpg]

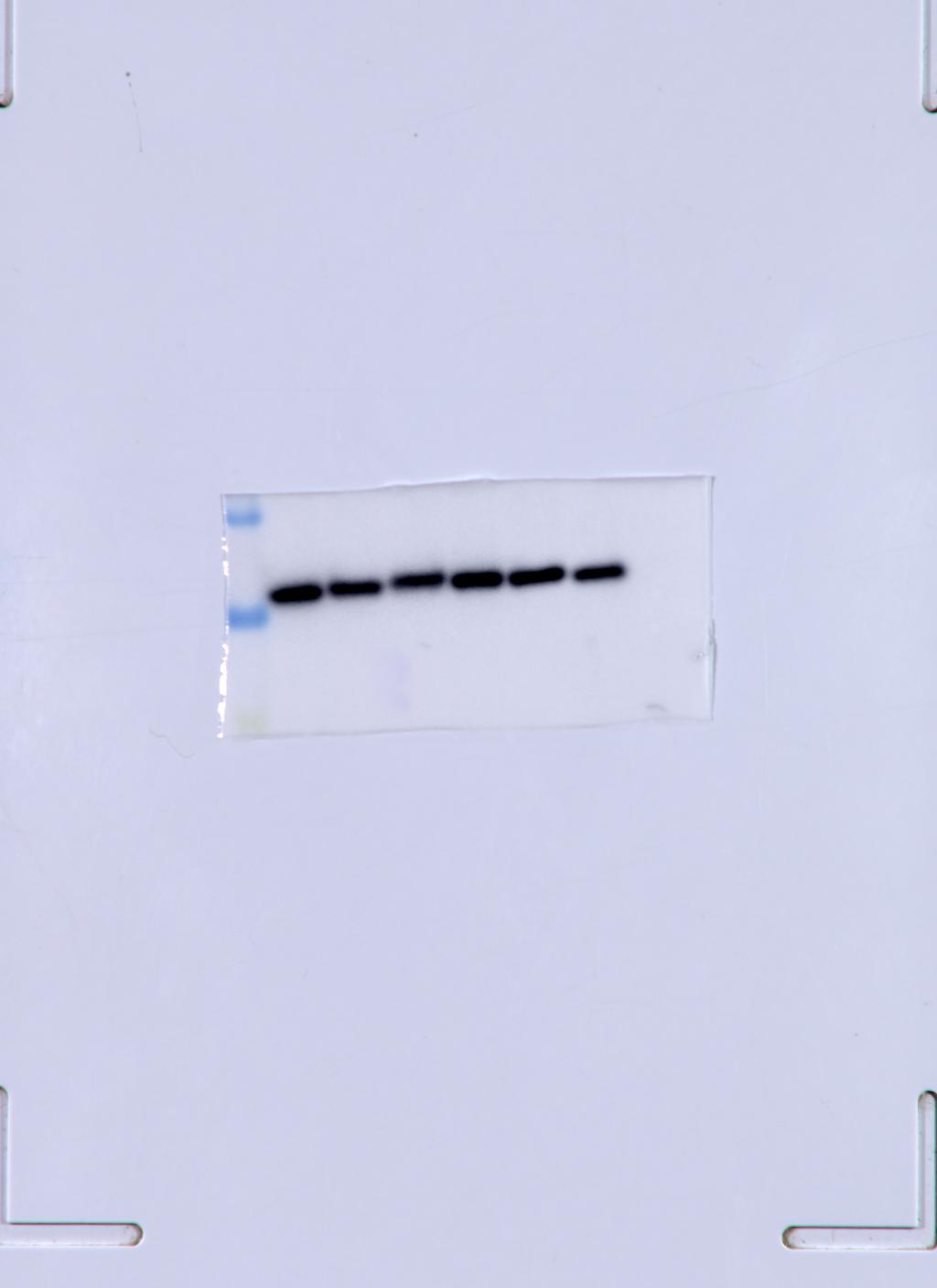

Supplement: Supplementary file 1 [file biology-11-01464-s001.zip › WB FIG/LH/lh1-8h-h3.2 2021.12.23_11.43.32_Ch+Marker.jpg]
